# First Report of the L925I kdr Mutation Associated with Pyrethroid Resistance in Genetically Distinct *Triatoma dimidiata*, Vector of Chagas Disease in Mexico

**DOI:** 10.3390/tropicalmed10070182

**Published:** 2025-06-27

**Authors:** Mario C. Saucedo-Montalvo, Jesus A. Davila-Barboza, Selene M. Gutierrez-Rodriguez, Beatriz Lopez-Monroy, Susana Favela-Lara, Iram P. Rodriguez-Sanchez, Guadalupe del C. Reyes-Solis, Cristina Bobadilla-Utrera, Adriana E. Flores

**Affiliations:** 1 Facultad de Ciencias Biologicas, Universidad Autonoma de Nuevo Leon, Av. Universidad s/n Cd. Universitaria, San Nicolas de los Garza NL 66455, Mexico; mario.saucedomntl@uanl.edu.mx (M.C.S.-M.); selene.gutierrezro@uanl.edu.mx (S.M.G.-R.); beatriz.lopezmr@uanl.edu.mx (B.L.-M.); susana.favelalr@uanl.edu.mx (S.F.-L.); iram.rodriguezsa@uanl.edu.mx (I.P.R.-S.); 2 Centro de Investigaciones Regionales Dr. Hideyo Noguchi, Universidad Autonoma de Yucatan, Merida 97000, Yucatan, Mexico; guadalupe.reyes@correo.uady.mx; 3 Laboratorio Estatal de Salud Publica, Servicios de Salud de Veracruz, Veracruz 91697, Mexico; cristibobadilla@gmail.com

**Keywords:** *Triatoma dimidiata*, genetic structure, mitochondrial markers, knockdown resistance, L925I mutation

## Abstract

*Triatoma dimidiata* is a widely distributed vector of *Trypanosoma cruzi* in Mexico and Central America, found across a range of habitats from sylvatic to domestic. Vector control has relied heavily on indoor residual spraying with pyrethroids; however, reinfestation and emerging resistance have limited its long-term effectiveness. In this study, we analyzed the genetic diversity and population structure of *T. dimidiata* from Veracruz, Oaxaca, and Yucatan using mitochondrial markers (cyt b and ND4) and screened for knockdown resistance (kdr)-type mutations in the voltage-gated sodium channel (VGSC) gene. High haplotype diversity and regional differentiation were observed, with most genetic variation occurring between populations. The ND4 marker provided greater resolution than cyt b, revealing ten haplotypes and supporting evidence of recent population expansion. Haplotype networks showed clear geographic segregation, particularly between populations east and west of the Isthmus of Tehuantepec. The L925I mutation, highly associated with pyrethroid resistance, was detected for the first time in Mexican populations of *T. dimidiata*, albeit at low frequencies. These findings highlight the importance of integrating population genetic data and resistance surveillance into regionally adapted vector control strategies for Chagas disease.

## 1. Introduction

Chagas disease, or American trypanosomiasis, is a potentially life-threatening illness caused by the protozoan parasite *Trypanosoma cruzi* (Chagas, 1909). Globally, an estimated 6 to 7 million people are infected, and approximately 75 million are at risk of acquiring the disease, mainly in 21 endemic countries across Latin America [1]. The vectors are blood-feeding insects of the subfamily Triatominae (Hemiptera: Reduviidae), which excrete the parasite in their feces or urine during or after feeding. Transmission occurs when the parasite enters the human host through mucous membranes or skin lesions contaminated with infected excreta. While vector-borne transmission remains predominant in the Americas, increased human mobility has facilitated the emergence of non-vectorial transmission routes in non-endemic areas, including congenital infection, blood transfusion, and organ transplantation [1,2,3].

A total of 35 triatomine species have been reported in Mexico, of which 19 are associated with human dwellings and considered potential vectors of *T. cruzi* [4]. These species are taxonomically grouped into the *Triatoma phyllosoma*, *Hospesneotomae protracta*, and *T. infestans* complexes, with the *T. phyllosoma* complex further subdivided into the *T. phyllosoma* and *T. dimidiata* subcomplexes [5]. Among them, *Triatoma dimidiata* (Latreille) is recognized as one of the most important vectors of Chagas disease in the Americas due to its wide distribution, its ability to colonize domestic environments, and its proven role in maintaining and transmitting *T. cruzi* to humans [6,7,8,9,10].

Among *Triatoma* species, vectorial capacity for *T. cruzi* transmission varies due to ecological and physiological traits such as the degree of domiciliation and defecation behavior during feeding. For instance, *Rhodnius prolixus*, a fully domiciliated species, tends to defecate soon after feeding, increasing transmission efficiency, while delayed defecation in other species can reduce the likelihood of transmission [11]. *Triatoma dimidiata*, widely distributed from southern Mexico through Central America and into northern South America, exhibits marked behavioral variability, ranging from intrusive to domiciliated forms depending on the region [12,13]. In the Yucatan Peninsula, it acts predominantly as an intrusive vector, showing seasonal house invasion and low colonization rates, yet maintaining a *T. cruzi* infection prevalence of approximately 28.7% in bugs [14] and reported human seroprevalence ranging from 1% to 5% in the region [15,16]. In Guatemala, *T. dimidiata* and *R. prolixus* exhibit comparable natural infection rates (~20%) [15], highlighting the epidemiological relevance of *T. dimidiata* despite its sylvatic, peridomestic, or intrusive behavior compared to the strictly domiciliated *R. prolixus*. While *T. dimidiata* may not always exhibit the high domesticity of other vector species, its broad geographic distribution, seasonal presence in human dwellings, and sustained transmission potential support its recognition as a major vector of *T. cruzi* in Mesoamerica [12,13].

In Mexico, *T. dimidiata* has been shown to be genetically structured into at least three major haplogroups (Hg1, Hg2, and Hg3), with distinct geographic distributions and varying degrees of genetic divergence. Haplogroup 1 is found primarily east of the Tehuantepec Isthmus, including the Yucatan Peninsula and northeastern Chiapas; haplogroup 2 has a wider distribution across central and southeastern Mexico, and haplogroup 3 is mostly restricted to Chiapas and further south into Central and South America [17]. These haplogroups exhibit significant genetic divergence, with differences of up to 14% in mitochondrial markers such as ND4, as well as distinct associations with *T. cruzi*, suggesting potential differences in their vectorial capacity [7,18,19]. The divergence of the *T. dimidiata* complex is estimated to have occurred around 0.85 to 0.97 million years ago, with subsequent diversification likely shaped by ecological and anthropogenic pressures. This level of genetic differentiation has led some authors to propose taxonomic revisions of the complex [17,20] and highlights the importance of considering genetic background when evaluating vector control strategies and insecticide resistance.

The use of pyrethroid insecticides remains one of the most widely implemented strategies for vector control in Mexico [21]. However, control failures have been reported, often attributed to the dispersal capacity of triatomine vectors and their ability to recolonize treated areas by moving between sylvatic, peridomestic, and domestic ecotopes. These reinfestations contribute to maintaining gene flow between populations, which may promote local adaptation and differences in insecticide susceptibility [22].

Resistance to pyrethroids in triatomines can result from multiple mechanisms, including metabolic detoxification through increased activity of esterases, glutathione S-transferases (GSTs), and cytochrome P450 monooxygenases [23], as well as target-site insensitivity caused by point mutations in the voltage-gated sodium channel (VGSC) gene, commonly known as knockdown resistance (kdr) [24,25]. These kdr mutations reduce the sensitivity of the insecticide target site, conferring resistance to both DDT and pyrethroids [26,27,28].

Although *T. dimidiata* has not been previously studied for the presence of kdr mutations in Mexico, several mutations have already been reported in other Mexican triatomine species. For instance, L1014F and L1014S were detected in natural populations of *T. pallidipennis* and *T. picturata* [29], while A943V and K964R were found in *T. mazzottii* and *T. longipennis*, respectively [30]. These findings provide molecular evidence that kdr mutations are circulating in Mexican triatomines and may represent an emerging resistance mechanism, particularly in species with known epidemiological relevance. Identifying the presence of kdr mutations in *T. dimidiata* is important for evaluating the emergence and potential spread of insecticide resistance in this key vector species.

The aim of this study was to analyze the genetic diversity and structure of *T. dimidiata* from the states of Veracruz, Yucatan, and Oaxaca in Mexico, using mitochondrial sequences from cytochrome b (cyt b) and NADH dehydrogenase subunit 4 (ND4). In addition, we screened knockdown resistance (kdr)-type mutations in the voltage-gated sodium channel (VGSC) gene, which are known molecular markers of pyrethroid resistance. This integrative approach analyzes the genetic structure and selective pressures linked to the emergence and spread of insecticide resistance in *T. dimidiata* from different regions of Mexico.

## 2. Materials and Methods

### 2.1. Biological Material

**Figure 1:**
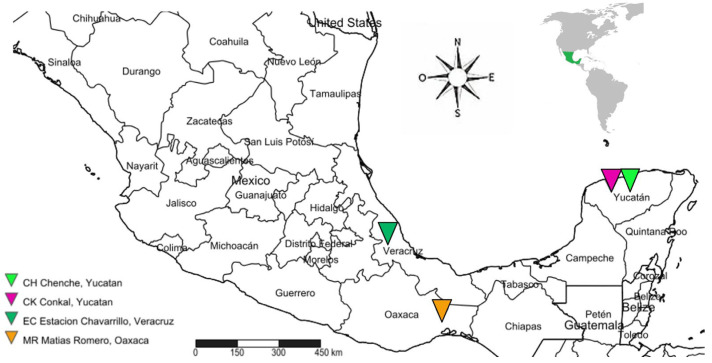
Sampling sites of *Triatoma dimidiata* in the states of Veracruz, Oaxaca, and Yucatan, Mexico.

Field populations of *T. dimidiata* were collected between 2019 and 2020 through active searches conducted in both indoor and outdoor environments in three Mexican states. In Veracruz, collections took place in Estacion Chavarrillo (EC), municipality of Emiliano Zapata (19°25′41.8800″ N, 96°48′33.8400″ W); in Yucatan, two locations were sampled: Chenche de las Torres (CH), municipality of Temax (21°07′47″ N, 88°58′53″ W), and the central area of Conkal (CK) municipality (21°4′25.14″ N, 89°31′11.82″ W); and in Oaxaca, collections were made in the central area of Matias Romero (MR) municipality (16°52′44.4000″ N, 95°2′21.8400″ W) (Figure 1). Searches were conducted in dwellings constructed from adobe and/or wood, as well as in peridomestic structures such as wood piles near chicken coops and areas where domestic animals were kept. All specimens were collected and taxonomically identified [31].

### 2.2. DNA Extraction

Genomic DNA was extracted using a modified salt-extraction protocol [32]. DNA was obtained from two legs of each triatomine specimen, with 20 individuals processed per locality. The legs from each specimen were placed together in a single 1.5 mL Eppendorf tube and processed individually. Samples were macerated in 50 µL of grinding buffer (0.1 M NaCl, 0.2 M sucrose, 0.1 M Tris-HCl pH 9.1, 0.05 M EDTA, and 0.5% sodium dodecyl sulfate), followed by the addition of another 50 µL of the same buffer to reach a final volume of 100 µL. Tubes were centrifuged at 13,000 rpm for 5 min, incubated at 65 °C for 30 min, and then 15 µL of 8 M potassium acetate was added. Samples were vortexed to homogenize the contents, incubated at −20 °C for 40 min, and centrifuged again at 13,000 rpm for 15 min. A total of 80 µL of the resulting supernatant was carefully transferred to new tubes. DNA was then precipitated with ethanol and centrifuged, and the pellets were washed sequentially with 70% and 100% ethanol, followed by centrifugation at 13,300 rpm. After ethanol removal, the pellets were dried in a dry bath incubator (Thermo Fisher Scientific, Pittsburgh, PA, USA) at 65 °C for 1 to 3 min and resuspended in 55 µL of molecular-grade water MilliQ (MilliporeSigma, St. Louis, MO, USA). DNA quality and purity were assessed spectrophotometrically using a NanoDrop 2000 (Thermo Scientific, Pittsburgh, PA, USA), and all samples were stored at −20 °C until further use in PCR.

### 2.3. Amplification and Sequencing of cyt b and ND4

The mitochondrial markers cytochrome b (cyt b) and NADH dehydrogenase subunit 4 (ND4) were amplified using primers previously reported [17,33] (Table S1). PCR conditions followed the protocols described in those studies (Table S2).

Each PCR reaction was carried out in a final volume of 25 µL, consisting of 5 µL of genomic DNA, 12.5 µL of GoTaq polymerase, 1 µL of each primer (forward and reverse), and 5.5 µL of sterile H_2_O. Amplifications were performed in a T100 Thermal Cycler (Bio-Rad, Hercules, CA, USA).

PCR products were separated by molecular weight using horizontal electrophoresis on 2% agarose gels prepared with SB buffer (0.044 M Tris, 0.044 M boric acid, 1.0 mM EDTA, pH 8.3). Electrophoresis was conducted at 100 V for 1 h and 10 min. Gels were visualized and photographed under UV light using a MultiDoc-It™ Imaging System (UVP, Upland, CA, USA). Verified PCR products were sent to Psomagen, Inc. (Rockville, MD, USA) for purification and standard Sanger sequencing.

Sequences for ND4 and cyt b were visualized, edited, and aligned using CodonCode Aligner v7.0.1 (CodonCode Corporation, Centerville, MA, USA) to evaluate the quality of forward reads and to obtain the corresponding reverse complement sequences. Subsequently, all sequences for each marker were aligned in MEGA version 11 [34]. The ND4 sequences were aligned against *T. dimidiata* reference sequences reported by Pech-May et al. [17] (ND4B: S1 File, https://doi.org/10.1371/journal.pntd.0007044.s004, accessed on 18 September 2024), which are available as supplementary material but were not deposited in GenBank. The ND4 sequences generated in the present study are available in Supplementary File S1. In contrast, the cyt b sequences were aligned using the *T. dimidiata* reference sequence AY859417, retrieved from the GenBank database. The cyt b sequences obtained in this study are available in Supplementary File S2.

Although 80 individuals were initially processed for DNA extraction, only 75 cyt b and 77 ND4 sequences passed quality control filters and were included in the final analyses. The discrepancy is due to variation in DNA quality and concentration across some samples, which affected PCR amplification and sequencing success.

### 2.4. Genetic Diversity and Population Differentiation

Genetic diversity and population differentiation were analyzed separately for the cyt b and ND4 genes. For each gene, we calculated the number of mutations (η), segregating sites (S), unique sites (Su), mean number of pairwise differences (k), number of haplotypes (Nh), haplotype diversity (Hd), nucleotide diversity (π), and the nucleotide polymorphism index (θ). Neutrality tests, including Tajima’s D and Fu’s Fs, were also conducted based on segregating sites. All analyses were performed using DnaSP v.6.12.03 [35].

Genetic differentiation among populations was assessed through pairwise F_ST_ values for each gene. The pairwise F_ST_ was calculated in Arlequin v.3.5 to estimate population differentiation based on differences in haplotype frequencies [36].

Genetic structure was evaluated using analysis of molecular variance (AMOVA) in PopArt v1.7 [37]. Separate analyses were conducted for the ND4 and cyt b genes to examine how genetic variation is distributed among and within populations. Statistical significance was assessed with 10,000 permutations.

### 2.5. Haplotype Network Construction

Sequences obtained for the cyt b, ND4, and VGSC genes were aligned using MEGA 11 [34], generating files in MEGA, FASTA, and NEXUS formats. Nucleotide composition and the number of haplotypes were analyzed using DnaSP v6.12.03 [35]. Haplotype frequencies for each population were then integrated into a NEXUS file and exported to Excel for haplotype characterization. Finally, haplotype networks were constructed in POPART 1.7 [37] using the Median-Joining algorithm to visualize evolutionary relationships among the identified haplotypes.

### 2.6. Screening for kdr-Type Mutations in the VGSC (Para) Gene

A fragment corresponding to domain II of the para gene, which encodes the voltage-gated sodium channel (VGSC), was amplified by PCR in a 25 µL reaction containing 5 µL of genomic DNA, 12.5 µL of GoTaq DNA polymerase (Promega, Madison, WI, USA), 1 µL of each primer, and 5.5 µL of sterile water. The primers used for *T. dimidiata* amplification were those previously reported [24,30] (Table S1), which target the IIS4–IIS6 region of domain II. This fragment includes codons where kdr-type mutations have been documented in triatomines, including L925I, L1014F/S, K964R, and A943V [24,25,29,30]. PCR amplifications were carried out in a T100 thermal cycler (Bio-Rad, CA, USA) using the conditions described in Table S2. Verified PCR products were sent to Psomagen, Inc. (Rockville, MD, USA) for purification and sequencing.

PCR products were separated by molecular weight through horizontal electrophoresis on 2% agarose gels prepared with SB buffer (0.044 M Tris, 0.044 M boric acid, 1.0 mM EDTA, pH 8.3). Electrophoresis was conducted at 100 V for 1 h 10 min. Gels were visualized and documented under UV light using a UV transilluminator (UVITEC, Cambridge, UK).

Sequence alignment and editing of the para gene sequences obtained from each specimen were carried out using MEGA v.11. Reference sequences included a *T. infestans* specimen carrying the L925I mutation (GenBank Accession No. KF179339) [25], a susceptible *T. infestans* strain (GenBank Accession No. KF179338), and a susceptible *T. dimidiata* strain [38]. The objective was to identify nucleotide substitutions potentially associated with pyrethroid resistance, including mutations previously reported in triatomines. The VGSC sequences generated in the present study are available in Supplementary File S3.

## 3. Results

### 3.1. Genetic Diversity and Population Structure

Seventy-five specimens of *T. dimidiata* were sequenced from four sites in Mexico using the 257 bp fragment of the cyt b gene (Table 1). Detailed metadata for all individuals analyzed, including sampling locality, year, habitat, haplotype or genotype, and population code for each marker (cyt b, ND4, and VGSC), is provided in Table S3.

This fragment showed 27 polymorphic sites (10.50%) and one unique site. A total of eight haplotypes were identified, ranging from one to five per locality. Globally, haplotype diversity was high (Hd ± SD = 0.799 ± 0.023), with the highest values in CK (0.732 ± 0.064) and MR (0.521 ± 0.042), while no haplotypic diversity was detected in EC. Nucleotide diversity and the polymorphism index were moderate to high (π ± SD = 0.044 and θ ± SD = 0.022, respectively). The overall neutrality test was highly significant for Fu’s Fs (Fs = 14.407, *p* < 0.0001) and also in CH (Fs = 8.772, *p* < 0.01), suggesting population expansion or directional selection. Tajima’s D was significantly positive in MR (D = 2.266, *p* < 0.05) and in the overall sample (D = 3.196, *p* < 0.01), suggesting the presence of population structure.

Seventy-seven sequences were analyzed using the 192 bp fragment of the ND4 gene. Thirteen polymorphic sites (6.77%) were detected, with no unique sites. Ten haplotypes were identified, ranging from two to four per site. Globally, haplotype diversity was high (Hd ± SD = 0.889 ± 0.012), with MR showing the highest local value (0.784 ± 0.035), followed by CH (0.608 ± 0.070). Nucleotide diversity and the polymorphism index were low to moderate (π ± SD = 0.025 and θ ± SD = 0.014, respectively). Neutrality tests showed mostly positive but non-significant values, except for Tajima’s D, which was significantly positive in MR (D = 2.642, *p* < 0.01) and in the overall sample (D = 2.258, *p* < 0.05), consistent with the presence of population structure.

Genetic differentiation between populations was high and significant for both fragments (Table 2). For cyt b, the greatest difference was observed between CK and MR (F_ST_ = 0.939, *p* < 0.01), while for ND4, the highest F_ST_ value was between CK and EC (F_ST_ = 0.797, *p* < 0.01).

AMOVA results showed consistent patterns of genetic structure across both mitochondrial markers. For the cyt b fragment, most of the variation (88.458%) was found among populations, with only 11.542% within populations. Similarly, for the ND4 fragment, 82.331% of the variation was attributed to differences among populations and 17.670% within populations (Table 3).

### 3.2. Mitochondrial Haplotype Networks

**Figure 2:**
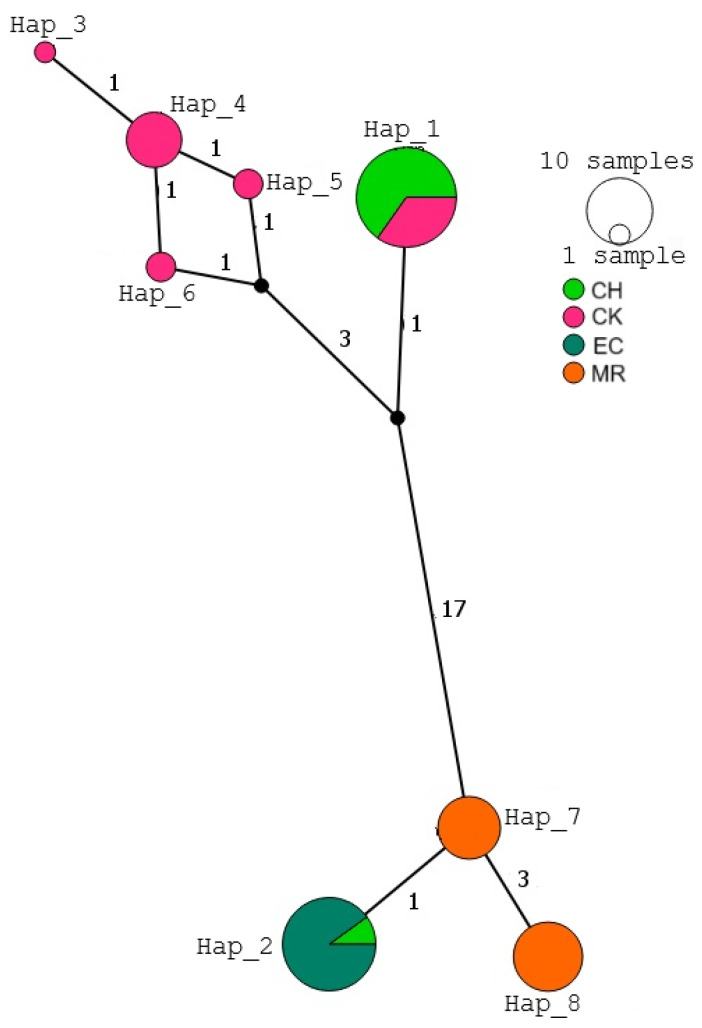
Median-joining haplotype network for *T. dimidiata* from Veracruz, Yucatan, and Oaxaca, Mexico, based on mitochondrial cyt b sequences. Each circle represents a unique haplotype, with its size proportional to the number of individuals sharing it. Colors indicate the geographic origin of each individual. Numbers in parentheses along the branches represent the number of mutational steps between haplotypes. CH (Chenche, Yucatan), CK (Conkal, Yucatan), EC (Estacion Chavarrillo, Veracruz), MR (Matias Romero, Oaxaca) (PopArt v1.7).

A total of eight haplotypes were identified for the cyt b fragment across the four sampling sites (Figure 2). Hap_1 was the most frequent (30.7%) and was shared by individuals from both Yucatan populations, while Hap_5, Hap_6, Hap_4, and Hap_3 were exclusive to Yucatan (CK). Hap_7 and Hap_8 were observed only in individuals from Oaxaca, and Hap_2 was found mainly in individuals from Veracruz, with one occurrence in Yucatan (CH). The network revealed two clearly separated haplogroups: one formed by closely related haplotypes from Yucatan and another more divergent group associated with Oaxaca and Veracruz.

**Figure 3:**
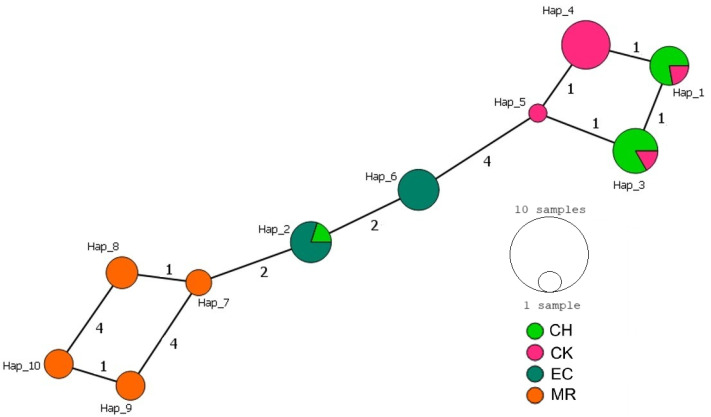
Median-joining haplotype network for *T. dimidiata* from Veracruz, Yucatan, and Oaxaca, Mexico, based on mitochondrial ND4 sequences. Each circle represents a unique haplotype, with its size proportional to the number of individuals sharing it. Colors indicate the geographic origin of the individuals. Numbers in parentheses along the branches represent the number of mutational steps between haplotypes. CH (Chenche, Yucatan), CK (Conkal, Yucatan), EC (Estacion Chavarrillo, Veracruz), MR (Matias Romero, Oaxaca) (PopArt v1.7).

A total of ten haplotypes were identified for the ND4 fragment across the four sampling sites (Figure 3). Hap_1 and Hap_3 were shared by individuals from both Yucatan populations, while Hap_4, which was the most frequent (18.2%), and Hap_5 were exclusive to Yucatan (CK). Hap_2 and Hap_6 were found in individuals from Veracruz, with Hap_2 also present in a sample from Yucatan (CH). Haplotypes Hap_7 to Hap_10 were observed exclusively in individuals from Oaxaca. The central portion of the network included haplotypes from Yucatan and Veracruz, while the most divergent and peripheral haplotypes formed a distinct cluster associated with Oaxaca.

Although both networks revealed geographic structuring among populations, the cyt b network exhibited a clearer separation between haplogroups, with a distinct divergence between Yucatan and Oaxaca. In contrast, the ND4 network showed a more continuous pattern of haplotype connectivity, suggesting more gradual genetic differentiation and broader sharing of haplotypes across regions.

### 3.3. Analysis of VGSC Mutations

**Figure 4:**
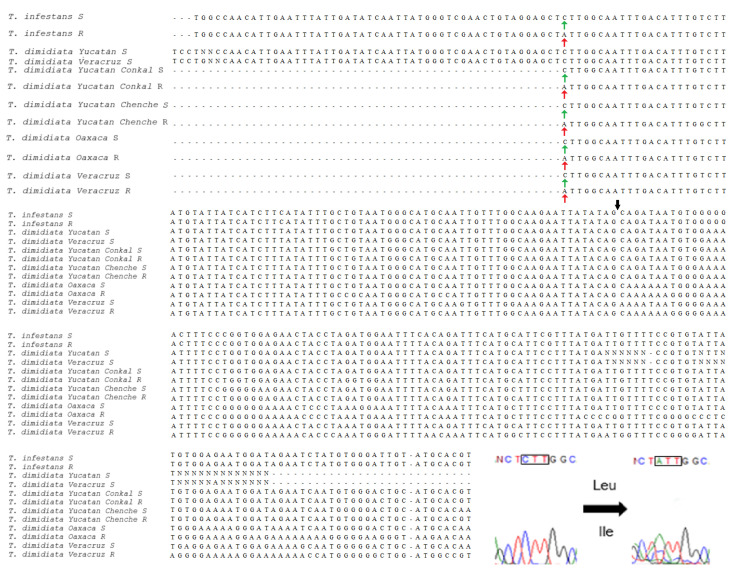
Alignment of partial *para* gene sequences from *T. dimidiata* populations in Mexico. Alignment includes reference sequences from *T. infestans* (susceptible and resistant) and both susceptible (S) and resistant (R) *T. dimidiata* individuals from laboratory and field-collected populations. The alignment highlights the L925I mutation site (black arrow), which corresponds to a CTT (Leu) to ATT (Ile) nucleotide substitution. Green arrows indicate the wild-type (susceptible) allele (CTT, Leu), while the red arrow indicates the mutated (resistant) allele (ATT, Ile). The intron–exon boundary is marked with a black arrow. The electropherogram at the bottom right confirms the point mutation at codon 925, showing the amino acid change from leucine to isoleucine.

The analysis of the partial para gene sequences from *T. dimidiata* revealed the presence of the L925I mutation in four individuals, each from a different population (Figure 4). This mutation was first reported in *T. infestans* from the Gran Chaco region and represents the earliest documented case of knockdown resistance in triatomines [25]. The L1014F mutation described by Fabro et al. [24] was not detected, nor were any of the substitutions previously reported for Mexican triatomines [29,30].

**Figure 5:**
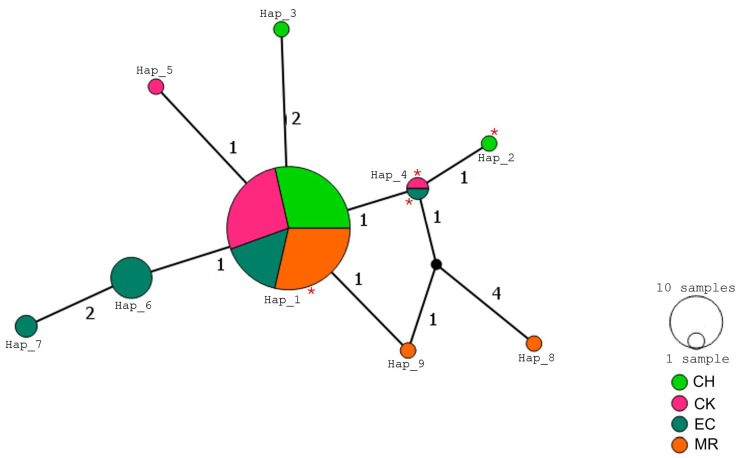
Median-joining haplotype network for *T. dimidiata* from Veracruz, Yucatan, and Oaxaca, Mexico, based on VGSC sequences. Each circle represents a unique haplotype, with its size proportional to the number of individuals sharing it. Colors indicate the geographic origin of the individuals. Numbers in parentheses along the branches represent the number of mutational steps between haplotypes. Red asterisks indicate haplotypes in which individuals carrying the L925I mutation were detected. CH (Chenche, Yucatan), CK (Conkal, Yucatan), EC (Estacion Chavarrillo, Veracruz), MR (Matias Romero, Oaxaca) (PopArt v1.7).

A total of ten haplotypes were identified from the partial sequence of the VGSC gene (Figure 5). Hap_1 was the most frequent (79.7%) and included individuals from all four populations. Most haplotypes were restricted to a single locality, and the network displayed a compact topology with limited geographic structuring. Hap_8 and Hap_9, both exclusive to Oaxaca, occupied peripheral positions in the network. The L925I mutation was detected in four individuals distributed across three haplotypes: one individual from Oaxaca in Hap_1, one from Yucatan (CH) in Hap_2, and two individuals, one from Veracruz and one from Yucatan (CK), in Hap_4.

## 4. Discussion

Understanding the genetic structure and insecticide resistance mechanisms of *T. dimidiata* is essential for developing effective and sustainable vector control strategies. Our study combined mitochondrial sequence analysis with the detection of kdr-type mutations to explore patterns of population differentiation and potential early signs of resistance in Mexican populations. The results provide relevant information regarding the distribution of genetic diversity and resistance-associated mutations, both of which have implications for the design of locally adapted control measures.

### 4.1. Genetic Diversity and Population Structure

The high haplotype diversity observed in both the cyt b and ND4 markers, particularly in Yucatan and Oaxaca, is consistent with previous studies reporting considerable genetic divergence among *T. dimidiata* populations throughout Mesoamerica [7,18,19].

The significant Fu’s Fs values and the positive Tajima’s D detected in MR and in the overall dataset suggest either population expansion or the existence of population structure. These patterns are further supported by AMOVA results, which indicated that most of the genetic variation is distributed among populations rather than within them. Consistent with findings from Guatemala and El Salvador, where gene flow was shown to be limited by geographic and ecological barriers [39], our results reveal clear genetic structuring among populations from Veracruz, Oaxaca, and Yucatan. This likely reflects historical isolation and restricted gene flow. Moreover, the absence of haplotype diversity in EC may be linked to anthropogenic disturbance, a factor previously associated with reduced genetic diversity in *T. dimidiata* populations [17].

The estimated divergence of the *T. dimidiata* complex, occurring approximately 0.97 million years ago (MYA) with a 95% highest posterior density (HPD) interval of 0.55–1.53 MYA, is consistent with the deep mitochondrial differentiation observed among the populations analyzed here [17]. These findings further support the existence of historically isolated lineages that may exhibit distinct ecological or epidemiological traits.

Our results also reinforce the utility of ND4 as a particularly informative mitochondrial marker for assessing genetic structure in *T. dimidiata*. Studies from Colombia have previously demonstrated that ND4 provides greater resolution than cyt b for detecting population structure [40,41]. Similarly, in our results, ND4 produced a higher number of haplotypes and greater haplotype diversity (Hd = 0.889) compared to cyt b, confirming its greater discriminatory power. Although nucleotide diversity and the polymorphism index were low to moderate (π = 0.0249; θ = 0.0138), these values, alongside the high haplotype diversity, are consistent with a pattern of recent population expansion, in line with neutrality tests and prior findings in southern Mexican populations [17].

### 4.2. Haplotype Networks and Geographic Differentiation

The median-joining networks constructed from both mitochondrial markers revealed strong geographic segregation of haplotypes, with most haplotypes restricted to specific regions and minimal sharing across localities. These patterns suggest limited gene flow among populations and support the presence of at least three distinct haplogroups, consistent with the phylogeographic structure previously described [17]. In particular, the cyt b haplotype network showed a clear division between a cluster composed of haplotypes from Yucatan and another formed by haplotypes from Oaxaca and Veracruz. The geographic separation observed here reflects similar structuring patterns described by Velásquez-Ortiz et al. [41] for Colombian populations and further supports the interpretation of the Isthmus of Tehuantepec as a significant geographic and ecological barrier [17]. Notably, one individual from Chenche (Yucatan) shared haplotype Hap_2 with specimens from Veracruz. Similar patterns have been reported by Pech-May et al. [17], who documented closely related haplotypes between samples from Yaxhá (Guatemala) and Palenque (Mexico) despite their geographic separation. Comparable cases of haplotype sharing among distant populations have also been observed in other vector species, such as *Lutzomyia longipalpis* (Diptera: Psychodidae) in South America [42].

In contrast, the ND4 network exhibited a more interconnected and homogeneous structure, with haplotypes shared across multiple regions and less defined geographic clustering. This discrepancy between markers may reflect differences in their mutation rates or lineage sorting histories [18,40]. The contrasting spatial resolution highlights the importance of using multiple mitochondrial markers in phylogeographic studies to obtain a more comprehensive understanding of population structure and evolutionary history.

### 4.3. Knockdown Resistance Mutations in the VGSC Para Gene

Studies on kdr-type mutations in Mexican triatomines remain scarce; however, previous reports have documented several substitutions in domain II of the para gene, including K964R and A943V in *T. mazzottii* and *T. longipennis*, respectively, as well as the L1014F/S mutations in *T. pallidipennis* and *T. picturata* [29,30]. The L1014F mutation has also been more broadly reported in *T. infestans* from the Gran Chaco, Argentina [24], but it was not detected in our *T. dimidiata* samples, nor were any of the mutations previously associated with resistance in species of the Phyllosoma complex.

In contrast, our study identified the presence of the L925I mutation in *T. dimidiata*, marking the first molecular evidence of a kdr-type mutation in this species in Mexico. This substitution was previously detected in *T. infestans* from the Gran Chaco [25], where it was associated with high levels of resistance to pyrethroids, with populations exhibiting resistance ratios (RRs) over 100-fold [43,44]. Notably, L925I has so far only been reported in hemipterans, such as *Bemisia tabaci* (Hemiptera: Aleyrodidae), *Cimex lectularius* (Hemiptera: Cimicidae), and *Trialeurodes vaporariorum* (Hemiptera: Aleyrodidae) [25,45,46], as well as in the mite *Varroa destructor* (Acari: Varroidae) [47].

Importantly, the functional relevance of the L925I mutation has been supported by electrophysiological characterization using in vitro expression systems. Specifically, when the mutation was introduced into the sodium channel of *Drosophila melanogaster* (Diptera: Drosophilidae) and expressed in *Xenopus laevis* (Anura: Pipidae) oocytes, it significantly decreased the potency of pyrethroids such as permethrin, deltamethrin, and fenfluthrin [48]. Further evidence from *T. infestans* links L925I to operational control failures [25], highlighting its value as a functionally confirmed marker of pyrethroid resistance.

In our study, the L925I mutation was detected in at least one individual from each of the four sampled localities: CH and CK (Yucatan), EC (Veracruz), and MR (Oaxaca). Its broad geographic distribution and low overall frequency (5.33%) suggest that the mutation may be in an early stage of selection. Considering that pyrethroids have been used extensively and continuously for vector control in Mexico, particularly against *Aedes aegypti* (Diptera: Culicidae) since the late 1990s [49,50], it is likely that this sustained chemical pressure is contributing to the emergence and maintenance of resistance alleles such as L925I. The continued use of these insecticides will likely promote the persistence and potential increase in the frequency of this mutation.

The limited distribution of L925I and its occurrence in three different haplotypes suggest either independent emergence events or early introgression, rather than fixation, as has been observed in other Mexican triatomines like *T. mazzottii* and *T. longipennis* [29,30]. Interestingly, a similar pattern was observed in *Anopheles stephensi* (Diptera: Culicidae) from eastern Ethiopia, where the L1014F mutation was present at low frequencies across various regions and was linked to a single downstream intron haplotype [51]. This suggests that knockdown resistance may initially emerge in a localized lineage and spread through migration or genetic introgression. Such parallels support the hypothesis that L925I in *T. dimidiata* may be under directional selection following historical mutation events rather than arising repeatedly.

Moreover, the detection of this mutation in genetically structured and geographically distant populations implies that resistance evolution in *T. dimidiata* could proceed in parallel, driven by region-specific selective pressures rather than the spread of a single resistant lineage [24]. While the L925I mutation was not associated here with phenotypic resistance levels, its presence, even at low frequencies, highlights the necessity of molecular surveillance, especially in populations where insecticides are extensively used. The association between the L925I mutation and pyrethroid resistance should be further investigated through susceptibility bioassays.

Understanding the current distribution and frequency of kdr resistance alleles is important for the development of effective resistance management strategies. Early detection can guide timely interventions before alleles become fixed, as has occurred in *T. infestans* populations from the Gran Chaco, where resistance to pyrethroids became widespread despite prior detection of kdr mutations [43,52].

While our study provides the first molecular evidence of the L925I mutation in *T. dimidiata* in Mexico, we recognize that the geographic scope was limited to four localities. Thus, our findings should be interpreted as an initial indication of potential resistance emergence rather than a comprehensive assessment. Broader surveillance across the species’ range is necessary to determine the full distribution and frequency of this mutation. Nonetheless, its detection in genetically distinct and geographically distant populations supports the hypothesis of early selection or multiple independent emergence events.

In this study, we specifically targeted the IIS4–IIS6 region of domain II of the para gene, as this fragment includes codons where all kdr-type mutations reported to date in triatomines have been identified, including L925I, L1014F/S, A943V, and K964R [24,25,29,30,43]. This region is functionally relevant, as it forms part of the pyrethroid binding site in the voltage-gated sodium channel and has been widely used for resistance monitoring in *T. infestans* from Argentina [24,25,43]; *T. pallidipennis*, *T. picturata*, *T. mazzottii*, and *T. longipennis* from Mexico [29,30]; and recently in *T. dimidiata* from Colombia, where transcriptomic analyses and sequencing of the same region were conducted, although no kdr-type mutations were detected [53].

### 4.4. Implications for Vector Control

The strong genetic structure observed among *T. dimidiata* populations in Mexico, as demonstrated in our study and in previous work [17], emphasizes the need for region-specific vector control strategies. Differences in haplotype composition, ecological preferences, and *T. cruzi* infection profiles among haplogroups are not solely of taxonomic relevance; they have direct implications for disease transmission dynamics and control efficacy.

The detection of kdr-type mutations like L925I in genetically divergent populations further highlights the importance of considering local resistance profiles when planning interventions. Uniform control measures may be ineffective given the genetic and phenotypic heterogeneity observed. The regional differences identified in our study, including exclusive haplotype clusters in Yucatan vs. Oaxaca and Veracruz and the localized presence of resistance-associated mutations, suggest that *T. dimidiata* populations may respond differently to insecticide-based control depending on their genetic background and historical exposure.

Additionally, the association between genetic structure and *T. cruzi* prevalence has been reported by Pech-May et al. [17], who identified differential infection patterns of *T. cruzi* discrete typing units (DTUs) among *T. dimidiata* haplogroups. Their findings suggest that certain haplotypes may be more competent vectors, reinforcing the importance of incorporating genetic and epidemiological data into vector control planning. This supports the need for surveillance strategies that not only track insecticide resistance but also consider the vectorial capacity of distinct lineages.

Previous studies have shown that reinfestation dynamics, driven by post-treatment survival and movement of vectors across sylvatic, peridomestic, and domestic ecotopes, can compromise the long-term success of control efforts [22,39]. These challenges are particularly relevant in settings with high anthropogenic disturbance, where ongoing selective pressures may further promote the development and spread of insecticide resistance.

In this context, integrating population genetics and resistance monitoring into vector control planning becomes essential. Targeted interventions that account for the genetic structure, ecological setting, and insecticide susceptibility of local populations are more likely to succeed in reducing vector populations and interrupting transmission. Further research is needed to assess the functional impact of resistance mutations such as L925I and their potential association with phenotypic resistance to inform evidence-based decision-making in Chagas disease control programs.

Insecticide resistance not only has operational implications but may also influence the persistence of *T. cruzi* transmission. Reduced efficacy of insecticide-based interventions in resistant populations can allow vector survival, reinfestation, and continued disease risk, particularly in endemic areas with inadequate surveillance [54,55]. This reinforces the need to integrate resistance monitoring into Chagas disease control programs, ensuring that vector control efforts are adapted to local resistance profiles to achieve lasting reductions in transmission.

Moreover, resistance can affect vector biology in ways that may influence transmission dynamics. Pleiotropic effects of resistance mechanisms have been associated with physiological and behavioral changes in *T. infestans*, such as altered defecation patterns, reduced dispersal capacity, and modified reproductive output [56,57]. While some of these traits may reduce vectorial capacity, others, like increased fecundity post-dispersal in resistant individuals, could offset control efforts [54]. Additionally, *T. cruzi* infection itself can interact with insecticide susceptibility, as shown in *T. infestans*, where infected susceptible insects exhibited higher mortality, while resistance in infected individuals remained unaffected [54]. These interactions highlight the complexity of resistance and infection dynamics, underlining the importance of integrated vector control strategies that consider both toxicological and epidemiological factors.

## 5. Conclusions

Our integrative analysis revealed significant genetic structure among *T. dimidiata* populations from Veracruz, Yucatan, and Oaxaca, supporting the existence of distinct haplogroups with potential epidemiological relevance. ND4 proved to be a more informative marker than cyt b for resolving population structure in this species. Importantly, we report for the first time in Mexico the presence of the L925I mutation in *T. dimidiata*, a kdr-type mutation previously associated with high resistance to pyrethroids in other triatomines. Although its frequency remains low, its presence in genetically divergent haplotypes and multiple geographic locations suggests early-stage selection and highlights the need for molecular surveillance. These findings emphasize the importance of designing vector control interventions based on local genetic backgrounds and resistance profiles. Ongoing surveillance of population structure and resistance mutations will be essential to support evidence-based and sustainable strategies for Chagas disease control in Mexico.

## Figures and Tables

**Table 1:** Genetic diversity indices of *T. dimidiata* populations from Mexico with the fragments cyt b and ND4.

Molecular Marker	Indices	Chenche	Conkal	Estacion Chavarrillo	Matias Romero	Overall
cyt b	N	17	20	18	20	75
	η	19	7	0	3	27
	S	19	7	0	3	27
	Su	0	1	0	0	1
	K	4.191	3.174	0	1.563	11.189
	Nh	2	5	1	2	8
	Hd ± SD	0.221 ± 0.121	0.732 ± 0.064	0	0.521 ± 0.042	0.799 ± 0.023
	π	0.016	0.013	0	0.006	0.044
	θ	0.022	0.008	-	0.003	0.022
	Fu’s Fs test	8.772 **	2.116	-	4.362	14.407 ****
	Tajima’s Test D	−1.002	2.000	-	2.266 *	3.196 **
ND4	N	19	20	18	20	77
	η	7	2	2	5	13
	S	7	2	2	5	13
	Su	0	0	0	0	0
	K	1.719	0.674	1.046	2.626	4.779
	Nh	3	4	2	4	10
	Hd ± SD	0.608 ± 0.070	0.505 ± 0.125	0.523 ± 0.047	0.784 ± 0.035	0.889 ± 0.012
	π	0.009	0.004	0.005	0.0137	0.025
	θ	0.010	0.003	0.003	0.007	0.014
	Fu’s Fs test	2.627	−0.899	2.953	2.686	2.782
	Tajima’s Test D	−0.473	0.457	1.949	2.642 **	2.258 *

CH: Chenche; CK: Conkal; EC: Estacion Chavarrillo; MR: Matias Romero; N: number of sequences; η: number of mutations; S: number of segregating sites; Su: number of unique sites; k: mean number of pairwise differences; Nh: number of haplotypes; Hd: haplotype diversity; π: nucleotide diversity; θ: nucleotide polymorphism index. SD: standard deviation. Neutrality tests: Fu’s Fs and Tajima’s D. Statistical significance: * *p* < 0.05 ** *p* < 0.01 *** *p* < 0.001 **** *p* < 0.0001.

**Table 2:** Pairwise F_ST_ genetic comparisons between *T. dimidiata* populations from Mexico. Below diagonal from the ND4 fragment, above diagonal from the cyt b fragment.

CytB/ND4	Chenche	Conkal	Estacion Chavarrillo	Matias Romero
Chenche		0.322 **	0.846 **	0.878 **
Conkal	0.326 **		0.894 **	0.922 **
Estacion Chavarrillo	0.753 **	0.797 **		0.694 **
Matias Romero	0.746 **	0.939 **	0.674 **	

Statistical significance: ** *p* < 0.01.

**Table 3:** Analysis of molecular variation (AMOVA) among populations of *T. dimidiata* from Mexico.

cyt b					
Source	df	Sum of Squares	Variance Components	Variation (%)	*p* Value
Among populations	31	6879.388	91.249	88.458	0.001
Within populations	71	845.359	11.906	11.542	
TOTAL	74	7724.747	103.155		
ND4					
Among populations	3	1061.155	13.667	82.331	0.001
Within populations	73	214.117	2.933	17.670	
TOTAL	76	1275.273	16.600		

## Data Availability

The mitochondrial gene sequences (cyt b and ND4) and the partial VGSC gene sequences are provided: File S1. Sequences of the ND4 fragment. File S2. Sequences of the cytb fragment. File S3. Sequences of the VGSC fragment.

## Supplementary Materials

The following supporting information can be downloaded at https://www.mdpi.com/article/10.3390/tropicalmed10070182/s1, Table S1: Primer sequences used for amplification of mitochondrial markers (cyt b and ND4) and the voltage-gated sodium channel (VGSC) gene in *T. dimidiata*; Table S2. Thermal cycling conditions used for the amplification of mitochondrial markers (cyt b, ND4) and the voltage-gated sodium channel (VGSC) gene in *T. dimidiata*; Table S3. Metadata of *T. dimidiata* individuals included in this study, with details by genetic marker (cyt b, ND4, and VGSC), including sample ID, locality, collection year, habitat type, haplotype or genotype designation, and population code. The data are organized in three separate spreadsheet tabs; File S1: Sequences of the ND4 fragment; File S2: Sequences of the cytb fragment; File S3: Sequences of the VGSC fragment.
