# Supplementary material for: First Report of the L925I kdr Mutation Associated with Pyrethroid Resistance in Genetically Distinct Triatoma dimidiata, Vector of Chagas Disease in Mexico
Source: Trop Med Infect Dis. 2025 Jun 27;10(7):182. doi: 10.3390/tropicalmed10070182 (PMC12299954; doi:10.3390/tropicalmed10070182)
Supplement: Supplementary file 1 [file tropicalmed-10-00182-s001.zip › File S1. Sequences_ND4_fragment.pdf]

>st41_TDCH_ND4_1_[organism=*Triatoma dimidiata] Triatoma dimidiata* _ NADH DEHYDROGENASE SUBUNIT 4 (ND4) GENE

TTGACTTCCAAGGGCTCATGTTGAGGCGCCTATTGCTGGTTCAATGATTTTAGCTGGT
GTTCTTTTAAAGTTGGGGGGTGGTCTTATACGTGTTTCTAATTTTATTGATTTGTTTAAGT
TTGGTGTTTTTATTGGTTTAAGACTTGGTGCTTTCTTGGTTGGGTTTTTATGTTTATACC
AGGTTGATATTAA

>st42_TDCH_ND4_2_[organism=*Triatoma dimidiata] Triatoma dimidiata* _ NADH DEHYDROGENASE SUBUNIT 4 (ND4) GENE

TTGACTTCCAAGGGCTCATGTTGAGGCGCCTATTGCTGGTTCAATGATTTTGGCTGGT
GTTCTTTTAAAGTTAGGGGGTGGTCTTATACGTGTTTCTAATTTTATTGGTTTGTTTAAGT
TTGGTGTTTTTATTGGTTTAAGACTTGGTGCTTTTTTGGTTGGGCTTTTATGTTTATATCA
GGTTGATATTAA

>st43_TDCH_ND4_3_[organism=*Triatoma dimidiata] Triatoma dimidiata* _ NADH DEHYDROGENASE SUBUNIT 4 (ND4) GENE

TTGACTTCCAAGGGCTCATGTTGAGGCGCCTATTGCTGGTTCAATGATTTTGGCTGGT
GTTCTTTTAAAGTTAGGGGGTGGTCTTATACGTGTTTCTAATTTTATTGGTTTGTTTAAGT
TTGGTGTTTTTATTGGTTTAAGACTTGGTGCTTTTTTGGTTGGGCTTTTATGTTTATATCA
GGTTGATATTAA

>st44_TDCH_ND4_4_[organism=*Triatoma dimidiata] Triatoma dimidiata* _ NADH DEHYDROGENASE SUBUNIT 4 (ND4) GENE

TTGACTTCCAAGGGCTCATGTTGAGGCGCCTATTGCTGGTTCAATGATTTTAGCTGGT
GTTCTTTTAAAGTTGGGGGGTGGTCTTATACGTGTTTCTAATTTTATTGATTTGTTTAAGT
TTGGTGTTTTTATTGGTTTAAGACTTGGTGCTTTCTTGGTTGGGTTTTTATGTTTATACC
AGGTTGATATTAA

>st45_TDCH_ND4_5_[organism=*Triatoma dimidiata] Triatoma dimidiata* _ NADH DEHYDROGENASE SUBUNIT 4 (ND4) GENE

TTGACTTCCAAGGGCTCATGTTGAGGCGCCTATTGCTGGTTCAATGATTTTAGCTGGT
GTTCTTTTAAAGTTGGGGGGTGGTCTTATACGTGTTTCTAATTTTATTGATTTGTTTAAGT
TTGGTGTTTTTATTGGTTTAAGACTTGGTGCTTTCTTGGTTGGGTTTTTATGTTTATACC
AGGTTGATATTAA

>st46_TDCH_ND4_6_[organism=*Triatoma dimidiata] Triatoma dimidiata* _ NADH DEHYDROGENASE SUBUNIT 4 (ND4) GENE

TTGACTTCCAAGGGCTCATGTTGAGGCGCCTATTGCTGGTTCAATGATTTTAGCTGGT
GTTCTTTTAAAGTTGGGGGGTGGTCTTATACGTGTTTCTAATTTTATTGATTTGTTTAAGT
TTGGTGTTTTTATTGGTTTAAGACTTGGTGCTTTCTTGGTTGGGTTTTTATGTTTATACC
AGGTTGATATTAA

>st47_TDCH_ND4_7_[organism=*Triatoma dimidiata] Triatoma dimidiata* _ NADH DEHYDROGENASE SUBUNIT 4 (ND4) GENE

TTGACTTCCAAGGGCTCATGTTGAGGCGCCTATTGCTGGTTCAATGATTTTAGCTGGT
GTTCTTTTAAAGTTGGGGGGTGGTCTTATACGTGTTTCTAATTTTATTGATTTGTTTAAGT

TTGGTGTTTTTATTGGTTTAAGACTTGGTGCTTTCTTGGTTGGGTTTTTATGTTTATACC
AGGTTGATATTAA

>st48_TDCH_ND4_8_[organism=*Triatoma dimidiata] Triatoma dimidiata* _ NADH DEHYDROGENASE SUBUNIT 4 (ND4) GENE

TTGACTTCCAAGGGCTCATGTTGAGGCGCCTATTGCTGGTTCAATGATTTTAGCTGGT
GTTCTTTTAAAGTTGGGGGGTGGTCTTATACGTGTTTCTAATTTTATTGATTTGTTTAAGT
TTGGTGTTTTTATTGGTTTAAGACTTGGTGCTTTCTTGGTTGGGTTTTTATGTTTATACC
AGGTTGATATTAA

>st49_TDCH_ND4_9_[organism=*Triatoma dimidiata] Triatoma dimidiata* _ NADH DEHYDROGENASE SUBUNIT 4 (ND4) GENE

TTGACTTCCAAGGGCTCATGTTGAGGCGCCTATTGCTGGTTCAATGATTTTAGCTGGT
GTTCTTTTAAAGTTGGGGGGTGGTCTTATACGTGTTTCTAATTTTATTGATTTGTTTAAGT
TTGGTGTTTTTATTGGTTTAAGACTTGGTGCTTTCTTGGTTGGGTTTTTATGTTTATACC
AGGTTGATATTAA

>st41_TDCH_ND4_11_[organism=*Triatoma dimidiata] Triatoma dimidiata* _ NADH DEHYDROGENASE SUBUNIT 4 (ND4) GENE

TTGACTTCCAAGGGCTCATGTTGAGGCGCCTATTGCTGGTTCAATGATTTTAGCTGGT
GTTCTTTTAAAGTTGGGGGGTGGTCTTATACGTGTTTCTAATTTTATTTATTTGTTTAAGT
TTGGTGTTTTTATTGGTTTAAGACTTGGTGCTTTCTTGGTTGGGTTTTTATGTTTATACC
AGGTTGATATTAA

>st42_TDCH_ND4_12_[organism=*Triatoma dimidiata] Triatoma dimidiata* _ NADH DEHYDROGENASE SUBUNIT 4 (ND4) GENE

TTGACTTCCAAGGGCTCATGTTGAGGCGCCTATTGCTGGTTCAATGATTTTAGCTGGT
GTTCTTTTAAAGTTGGGGGGTGGTCTTATACGTGTTTCTAATTTTATTTATTTGTTTAAGT
TTGGTGTTTTTATTGGTTTAAGACTTGGTGCTTTCTTGGTTGGGTTTTTATGTTTATACC
AGGTTGATATTAA

>st43_TDCH_ND4_13_[organism=*Triatoma dimidiata] Triatoma dimidiata* _ NADH DEHYDROGENASE SUBUNIT 4 (ND4) GENE

TTGACTTCCAAGGGCTCATGTTGAGGCGCCTATTGCTGGTTCAATGATTTTAGCTGGT
GTTCTTTTAAAGTTGGGGGGTGGTCTTATACGTGTTTCTAATTTTATTTATTTGTTTAAGT
TTGGTGTTTTTATTGGTTTAAGACTTGGTGCTTTCTTGGTTGGGTTTTTATGTTTATACC
AGGTTGATATTAA

>st44_TDCH_ND4_14_[organism=*Triatoma dimidiata] Triatoma dimidiata* _ NADH DEHYDROGENASE SUBUNIT 4 (ND4) GENE

TTGACTTCCAAGGGCTCATGTTGAGGCGCCTATTGCTGGTTCAATGATTTTAGCTGGT
GTTCTTTTAAAGTTGGGGGGTGGTCTTATACGTGTTTCTAATTTTATTTATTTGTTTAAGT
TTGGTGTTTTTATTGGTTTAAGACTTGGTGCTTTCTTGGTTGGGTTTTTATGTTTATACC
AGGTTGATATTAA

>st45_TDCH_ND4_15_[organism=*Triatoma dimidiata] Triatoma dimidiata* _ NADH DEHYDROGENASE SUBUNIT 4 (ND4) GENE

TTGACTTCCAAGGGCTCATGTTGAGGCGCCTATTGCTGGTTCAATGATTTTAGCTGGT
GTTCTTTTAAAGTTGGGGGGTGGTCTTATACGTGTTTCTAATTTTATTTATTTGTTTAAGT
TTGGTGTTTTTATTGGTTTAAGACTTGGTGCTTTCTTGGTTGGGTTTTTATGTTTATACC
AGGTTGATATTAA

>st46_TDCH_ND4_16_[organism=*Triatoma dimidiata] Triatoma dimidiata* _ NADH DEHYDROGENASE SUBUNIT 4 (ND4) GENE

TTGACTTCCAAGGGCTCATGTTGAGGCGCCTATTGCTGGTTCAATGATTTTAGCTGGT
GTTCTTTTAAAGTTGGGGGGTGGTCTTATACGTGTTTCTAATTTTATTTATTTGTTTAAGT
TTGGTGTTTTTATTGGTTTAAGACTTGGTGCTTTCTTGGTTGGGTTTTTATGTTTATACC
AGGTTGATATTAA

>st47_TDCH_ND4_17_[organism=*Triatoma dimidiata] Triatoma dimidiata* _ NADH DEHYDROGENASE SUBUNIT 4 (ND4) GENE

TTGACTTCCAAGGGCTCATGTTGAGGCGCCTATTGCTGGTTCAATGATTTTAGCTGGT
GTTCTTTTAAAGTTGGGGGGTGGTCTTATACGTGTTTCTAATTTTATTTATTTGTTTAAGT
TTGGTGTTTTTATTGGTTTAAGACTTGGTGCTTTCTTGGTTGGGTTTTTATGTTTATACC
AGGTTGATATTAA

>st48_TDCH_ND4_18_[organism=*Triatoma dimidiata] Triatoma dimidiata* _ NADH DEHYDROGENASE SUBUNIT 4 (ND4) GENE

TTGACTTCCAAGGGCTCATGTTGAGGCGCCTATTGCTGGTTCAATGATTTTAGCTGGT
GTTCTTTTAAAGTTGGGGGGTGGTCTTATACGTGTTTCTAATTTTATTTATTTGTTTAAGT
TTGGTGTTTTTATTGGTTTAAGACTTGGTGCTTTCTTGGTTGGGTTTTTATGTTTATACC
AGGTTGATATTAA

>st49_TDCH_ND4_19_[organism=*Triatoma dimidiata] Triatoma dimidiata* _ NADH DEHYDROGENASE SUBUNIT 4 (ND4) GENE

TTGACTTCCAAGGGCTCATGTTGAGGCGCCTATTGCTGGTTCAATGATTTTAGCTGGT
GTTCTTTTAAAGTTGGGGGGTGGTCTTATACGTGTTTCTAATTTTATTTATTTGTTTAAGT
TTGGTGTTTTTATTGGTTTAAGACTTGGTGCTTTCTTGGTTGGGTTTTTATGTTTATACC
AGGTTGATATTAA

>st50_TDCH_ND4_20_[organism=*Triatoma dimidiata] Triatoma dimidiata* _ NADH DEHYDROGENASE SUBUNIT 4 (ND4) GENE

TTGACTTCCAAGGGCTCATGTTGAGGCGCCTATTGCTGGTTCAATGATTTTAGCTGGT
GTTCTTTTAAAGTTGGGGGGTGGTCTTATACGTGTTTCTAATTTTATTTATTTGTTTAAGT
TTGGTGTTTTTATTGGTTTAAGACTTGGTGCTTTCTTGGTTGGGTTTTTATGTTTATACC
AGGTTGATATTAA

>st51_TDCK_ND4_1_[organism=*Triatoma dimidiata] Triatoma dimidiata* _ NADH DEHYDROGENASE SUBUNIT 4 (ND4) GENE

TTGACTTCCAAGGGCTCATGTTGAGGCGCCTATTGCTGGTTCAATGATTTTAGCTGGT
GTTCTTTTAAAGTTGGGGGGTGGTCTTATACGTGTTTCTAATTTTATTGATTTGTTTAAGT
TTGGTGTTTTTATTGGTTTAAGACTTGGTGCTTTCTTGGTTGGGTTTTTATGTTTATATCA
GGTTGATATTAA

>st52_TDCK_ND4_2_[organism=*Triatoma dimidiata] Triatoma dimidiata* _ NADH DEHYDROGENASE SUBUNIT 4 (ND4) GENE

TTGACTTCCAAGGGCTCATGTTGAGGCGCCTATTGCTGGTTCAATGATTTTAGCTGGT
GTTCTTTTAAAGTTGGGGGGTGGTCTTATACGTGTTTCTAATTTTATTGATTTGTTTAAGT
TTGGTGTTTTTATTGGTTTAAGACTTGGTGCTTTCTTGGTTGGGTTTTTATGTTTATATCA
GGTTGATATTAA

>st53_TDCK_ND4_3_[organism=*Triatoma dimidiata] Triatoma dimidiata* _ NADH DEHYDROGENASE SUBUNIT 4 (ND4) GENE

TTGACTTCCAAGGGCTCATGTTGAGGCGCCTATTGCTGGTTCAATGATTTTAGCTGGT
GTTCTTTTAAAGTTGGGGGGTGGTCTTATACGTGTTTCTAATTTTATTGATTTGTTTAAGT
TTGGTGTTTTTATTGGTTTAAGACTTGGTGCTTTCTTGGTTGGGTTTTTATGTTTATATCA
GGTTGATATTAA

>st54_TDCK_ND4_4_[organism=*Triatoma dimidiata] Triatoma dimidiata* _ NADH DEHYDROGENASE SUBUNIT 4 (ND4) GENE

TTGACTTCCAAGGGCTCATGTTGAGGCGCCTATTGCTGGTTCAATGATTTTAGCTGGT
GTTCTTTTAAAGTTGGGGGGTGGTCTTATACGTGTTTCTAATTTTATTGATTTGTTTAAGT
TTGGTGTTTTTATTGGTTTAAGACTTGGTGCTTTCTTGGTTGGGTTTTTATGTTTATATCA
GGTTGATATTAA

>st55_TDCK_ND4_5_[organism=*Triatoma dimidiata] Triatoma dimidiata* _ NADH DEHYDROGENASE SUBUNIT 4 (ND4) GENE

TTGACTTCCAAGGGCTCATGTTGAGGCGCCTATTGCTGGTTCAATGATTTTAGCTGGT
GTTCTTTTAAAGTTGGGGGGTGGTCTTATACGTGTTTCTAATTTTATTGATTTGTTTAAGT
TTGGTGTTTTTATTGGTTTAAGACTTGGTGCTTTCTTGGTTGGGTTTTTATGTTTATATCA
GGTTGATATTAA

>st56_TDCK_ND4_6_[organism=*Triatoma dimidiata] Triatoma dimidiata* _ NADH DEHYDROGENASE SUBUNIT 4 (ND4) GENE

TTGACTTCCAAGGGCTCATGTTGAGGCGCCTATTGCTGGTTCAATGATTTTAGCTGGT
GTTCTTTTAAAGTTGGGGGGTGGTCTTATACGTGTTTCTAATTTTATTGATTTGTTTAAGT
TTGGTGTTTTTATTGGTTTAAGACTTGGTGCTTTCTTGGTTGGGTTTTTATGTTTATATCA
GGTTGATATTAA

>st57_TDCK_ND4_7_[organism=*Triatoma dimidiata] Triatoma dimidiata* _ NADH DEHYDROGENASE SUBUNIT 4 (ND4) GENE

TTGACTTCCAAGGGCTCATGTTGAGGCGCCTATTGCTGGTTCAATGATTTTAGCTGGT
GTTCTTTTAAAGTTGGGGGGTGGTCTTATACGTGTTTCTAATTTTATTGATTTGTTTAAGT
TTGGTGTTTTTATTGGTTTAAGACTTGGTGCTTTCTTGGTTGGGTTTTTATGTTTATATCA
GGTTGATATTAA

>st58_TDCK_ND4_8_[organism=*Triatoma dimidiata] Triatoma dimidiata* _ NADH DEHYDROGENASE SUBUNIT 4 (ND4) GENE

TTGACTTCCAAGGGCTCATGTTGAGGCGCCTATTGCTGGTTCAATGATTTTAGCTGGT
GTTCTTTTAAAGTTGGGGGGTGGTCTTATACGTGTTTCTAATTTTATTGATTTGTTTAAGT

TTGGTGTTTTTATTGGTTTAAGACTTGGTGCTTTCTTGGTTGGGTTTTTATGTTTATATCA
GGTTGATATTAA

>st59_TDCK_ND4_9_[organism=*Triatoma dimidiata] Triatoma dimidiata* _ NADH DEHYDROGENASE SUBUNIT 4 (ND4) GENE

TTGACTTCCAAGGGCTCATGTTGAGGCGCCTATTGCTGGTTCAATGATTTTAGCTGGT
GTTCTTTTAAAGTTGGGGGGTGGTCTTATACGTGTTTCTAATTTTATTGATTTGTTTAAGT
TTGGTGTTTTTATTGGTTTAAGACTTGGTGCTTTCTTGGTTGGGTTTTTATGTTTATATCA
GGTTGATATTAA

>st60_TDCK_ND4_10_[organism=*Triatoma dimidiata] Triatoma dimidiata* _ NADH DEHYDROGENASE SUBUNIT 4 (ND4) GENE

TTGACTTCCAAGGGCTCATGTTGAGGCGCCTATTGCTGGTTCAATGATTTTAGCTGGT
GTTCTTTTAAAGTTGGGGGGTGGTCTTATACGTGTTTCTAATTTTATTTATTTGTTTAAGT
TTGGTGTTTTTATTGGTTTAAGACTTGGTGCTTTCTTGGTTGGGTTTTTATGTTTATATCA
GGTTGATATTAA

>st91_TDCK_ND4_11_[organism=*Triatoma dimidiata] Triatoma dimidiata* _ NADH DEHYDROGENASE SUBUNIT 4 (ND4) GENE

TTGACTTCCAAGGGCTCATGTTGAGGCGCCTATTGCTGGTTCAATGATTTTAGCTGGT
GTTCTTTTAAAGTTGGGGGGTGGTCTTATACGTGTTTCTAATTTTATTGATTTGTTTAAGT
TTGGTGTTTTTATTGGTTTAAGACTTGGTGCTTTCTTGGTTGGGTTTTTATGTTTATACC
AGGTTGATATTAA

>st92_TDCK_ND4_12_[organism=*Triatoma dimidiata] Triatoma dimidiata* _ NADH DEHYDROGENASE SUBUNIT 4 (ND4) GENE

TTGACTTCCAAGGGCTCATGTTGAGGCGCCTATTGCTGGTTCAATGATTTTAGCTGGT
GTTCTTTTAAAGTTGGGGGGTGGTCTTATACGTGTTTCTAATTTTATTTATTTGTTTAAGT
TTGGTGTTTTTATTGGTTTAAGACTTGGTGCTTTCTTGGTTGGGTTTTTATGTTTATATCA
GGTTGATATTAA

>st93_TDCK_ND4_13_[organism=*Triatoma dimidiata] Triatoma dimidiata* _ NADH DEHYDROGENASE SUBUNIT 4 (ND4) GENE

TTGACTTCCAAGGGCTCATGTTGAGGCGCCTATTGCTGGTTCAATGATTTTAGCTGGT
GTTCTTTTAAAGTTGGGGGGTGGTCTTATACGTGTTTCTAATTTTATTGATTTGTTTAAGT
TTGGTGTTTTTATTGGTTTAAGACTTGGTGCTTTCTTGGTTGGGTTTTTATGTTTATACC
AGGTTGATATTAA

>st94_TDCK_ND4_14_[organism=*Triatoma dimidiata] Triatoma dimidiata* _ NADH DEHYDROGENASE SUBUNIT 4 (ND4) GENE

TTGACTTCCAAGGGCTCATGTTGAGGCGCCTATTGCTGGTTCAATGATTTTAGCTGGT
GTTCTTTTAAAGTTGGGGGGTGGTCTTATACGTGTTTCTAATTTTATTGATTTGTTTAAGT
TTGGTGTTTTTATTGGTTTAAGACTTGGTGCTTTCTTGGTTGGGTTTTTATGTTTATATCA
GGTTGATATTAA

>st95_TDCK_ND4_15_[organism=*Triatoma dimidiata] Triatoma dimidiata* _ NADH DEHYDROGENASE SUBUNIT 4 (ND4) GENE

TTGACTTCCAAGGGCTCATGTTGAGGCGCCTATTGCTGGTTCAATGATTTTAGCTGGT
GTTCTTTTAAAGTTGGGGGGTGGTCTTATACGTGTTTCTAATTTTATTGATTTGTTTAAGT
TTGGTGTTTTTATTGGTTTAAGACTTGGTGCTTTCTTGGTTGGGTTTTTATGTTTATATCA
GGTTGATATTAA

>st96_TDCK_ND4_16_[organism=*Triatoma dimidiata] Triatoma dimidiata* _ NADH DEHYDROGENASE SUBUNIT 4 (ND4) GENE

TTGACTTCCAAGGGCTCATGTTGAGGCGCCTATTGCTGGTTCAATGATTTTAGCTGGT
GTTCTTTTAAAGTTGGGGGGTGGTCTTATACGTGTTTCTAATTTTATTGATTTGTTTAAGT
TTGGTGTTTTTATTGGTTTAAGACTTGGTGCTTTCTTGGTTGGGTTTTTATGTTTATATCA
GGTTGATATTAA

>st97_TDCK_ND4_17_[organism=*Triatoma dimidiata] Triatoma dimidiata* _ NADH DEHYDROGENASE SUBUNIT 4 (ND4) GENE

TTGACTTCCAAGGGCTCATGTTGAGGCGCCTATTGCTGGTTCAATGATTTTAGCTGGT
GTTCTTTTAAAGTTGGGGGGTGGTCTTATACGTGTTTCTAATTTTATTGATTTGTTTAAGT
TTGGTGTTTTTATTGGTTTAAGACTTGGTGCTTTCTTGGTTGGGTTTTTATGTTTATATCA
GGTTGATATTAA

>st98_TDCK_ND4_18_[organism=*Triatoma dimidiata] Triatoma dimidiata* _ NADH DEHYDROGENASE SUBUNIT 4 (ND4) GENE

TTGACTTCCAAGGGCTCATGTTGAGGCGCCTATTGCTGGTTCAATGATTTTAGCTGGT
GTTCTTTTAAAGTTGGGGGGTGGTCTTATACGTGTTTCTAATTTTATTTATTTGTTTAAGT
TTGGTGTTTTTATTGGTTTAAGACTTGGTGCTTTCTTGGTTGGGTTTTTATGTTTATACC
AGGTTGATATTAA

>st99_TDCK_ND4_19_[organism=*Triatoma dimidiata] Triatoma dimidiata* _ NADH DEHYDROGENASE SUBUNIT 4 (ND4) GENE

TTGACTTCCAAGGGCTCATGTTGAGGCGCCTATTGCTGGTTCAATGATTTTAGCTGGT
GTTCTTTTAAAGTTGGGGGGTGGTCTTATACGTGTTTCTAATTTTATTGATTTGTTTAAGT
TTGGTGTTTTTATTGGTTTAAGACTTGGTGCTTTCTTGGTTGGGTTTTTATGTTTATATCA
GGTTGATATTAA

>st100_TDCK_ND4_20_[organism=*Triatoma dimidiata] Triatoma dimidiata* _ NADH DEHYDROGENASE SUBUNIT 4 (ND4) GENE

TTGACTTCCAAGGGCTCATGTTGAGGCGCCTATTGCTGGTTCAATGATTTTAGCTGGT
GTTCTTTTAAAGTTGGGGGGTGGTCTTATACGTGTTTCTAATTTTATTTATTTGTTTAAGT
TTGGTGTTTTTATTGGTTTAAGACTTGGTGCTTTCTTGGTTGGGTTTTTATGTTTATACC
AGGTTGATATTAA

>st61_TDEC_ND4_1_[organism=*Triatoma dimidiata] Triatoma dimidiata* _ NADH DEHYDROGENASE SUBUNIT 4 (ND4) GENE

TTGACTTCCAAGGGCTCATGTTGAGGCGCCTATTGCTGGTTCAATGATTTTGGCTGGT
GTTCTTTTAAAGTTAGGGGGTGGTCTTATACGTGTTTCTAATTTTATTGGTTTGTTTAAGT
TTGGTGTTTTTATTGGTTTAAGACTTGGTGCTTTTTTGGTTGGGCTTTTATGTTTATATCA
GGTTGATATTAA

>st62_TDEC_ND4_2_[organism=*Triatoma dimidiata] Triatoma dimidiata* _ NADH DEHYDROGENASE SUBUNIT 4 (ND4) GENE

TTGACTTCCAAGGGCTCATGTTGAGGCGCCTATTGCTGGTTCAATGATTTTGGCTGGT
GTTCTTTTAAAGTTAGGGGGTGGTCTTATACGTGTTTCTAATTTTATTGGTTTGTTTAAGT
TTGGTGTTTTTATTGGTTTAAGACTTGGTGCTTTTTTGGTTGGGCTTTTATGTTTATATCA
GGTTGATATTAA

>st63_TDEC_ND4_3_[organism=*Triatoma dimidiata] Triatoma dimidiata* _ NADH DEHYDROGENASE SUBUNIT 4 (ND4) GENE

TTGACTTCCAAGGGCTCATGTTGAGGCGCCTATTGCTGGTTCAATGATTTTGGCTGGT
GTTCTTTTAAAGTTAGGGGGTGGTCTTATACGTGTTTCTAATTTTATTGGTTTGTTTAAGT
TTGGTGTTTTTATTGGTTTAAGACTTGGTGCTTTTTTGGTTGGGCTTTTATGTTTATATCA
GGTTGATATTAA

>st64_TDEC_ND4_4_[organism=*Triatoma dimidiata] Triatoma dimidiata* _ NADH DEHYDROGENASE SUBUNIT 4 (ND4) GENE

TTGACTTCCAAGGGCTCATGTTGAGGCGCCTATTGCTGGTTCAATGATTTTGGCTGGT
GTTCTTTTAAAGTTAGGGGGTGGTCTTATACGTGTTTCTAATTTTATTGGTTTGTTTAAGT
TTGGTGTTTTTATTGGTTTAAGACTTGGTGCTTTTTTGGTTGGGCTTTTATGTTTATATCA
GGTTGATATTAA

>st65_TDEC_ND4_5_[organism=*Triatoma dimidiata] Triatoma dimidiata* _ NADH DEHYDROGENASE SUBUNIT 4 (ND4) GENE

TTGACTTCCAAGGGCTCATGTTGAGGCGCCTATTGCTGGTTCAATGATTTTGGCTGGT
GTTCTTTTAAAGTTAGGGGGTGGTCTTATACGTGTTTCTAATTTTATTGGTTTGTTTAAGT
TTGGTGTTTTTATTGGTTTAAGACTTGGTGCTTTTTTGGTTGGGCTTTTATGTTTATATCA
GGTTGATATTAA

>st66_TDEC_ND4_6_[organism=*Triatoma dimidiata] Triatoma dimidiata* _ NADH DEHYDROGENASE SUBUNIT 4 (ND4) GENE

TTGACTTCCAAGGGCTCATGTTGAGGCGCCTATTGCTGGTTCAATGATTTTGGCTGGT
GTTCTTTTAAAGTTAGGGGGTGGTCTTATACGTGTTTCTAATTTTATTGGTTTGTTTAAGT
TTGGTGTTTTTATTGGTTTAAGACTTGGTGCTTTTTTGGTTGGGCTTTTATGTTTATATCA
GGTTGATATTAA

>st67_TDEC_ND4_7_[organism=*Triatoma dimidiata] Triatoma dimidiata* _ NADH DEHYDROGENASE SUBUNIT 4 (ND4) GENE

TTGACTTCCAAGGGCTCATGTTGAGGCGCCTATTGCTGGTTCAATGATTTTGGCTGGT
GTTCTTTTAAAGTTAGGGGGTGGTCTTATACGTGTTTCTAATTTTATTGGTTTGTTTAAGT
TTGGTGTTTTTATTGGTTTAAGACTTGGTGCTTTTTTGGTTGGGCTTTTATGTTTATATCA
GGTTGATATTAA

>st68_TDEC_ND4_8_[organism=*Triatoma dimidiata] Triatoma dimidiata* _ NADH DEHYDROGENASE SUBUNIT 4 (ND4) GENE

TTGACTTCCAAGGGCTCATGTTGAGGCGCCTATTGCTGGTTCAATGATTTTGGCTGGT
GTTCTTTTAAAGTTAGGGGGTGGTCTTATACGTGTTTCTAATTTTATTGGTTTGTTTAAGT

TTGGTGTTTTTATTGGTTTAAGACTTGGTGCTTTTTTGGTTGGGCTTTTATGTTTATATCA
GGTTGATATTAA

>st61_TDEC_ND4_11_[organism=*Triatoma dimidiata] Triatoma dimidiata* _ NADH DEHYDROGENASE SUBUNIT 4 (ND4) GENE

TTGACTTCCAAGGGCTCATGTTGAGGCGCCTATTGCTGGTTCAATGATTTTGGCTGGT
GTTCTTTTAAAGTTAGGGGGTGGTCTTATACGTGTTTCTAATTTTATTTATTTGTTTAAGT
TTGGTGTTTTTATTGGTTTAAGACTTGGTGCTTTTTTGGTTGGGCTTTTATGTTTATATCA
GGTTGATATTAA

>st62_TDEC_ND4_12_[organism=*Triatoma dimidiata] Triatoma dimidiata* _ NADH DEHYDROGENASE SUBUNIT 4 (ND4) GENE

TTGACTTCCAAGGGCTCATGTTGAGGCGCCTATTGCTGGTTCAATGATTTTGGCTGGT
GTTCTTTTAAAGTTAGGGGGTGGTCTTATACGTGTTTCTAATTTTATTTATTTGTTTAAGT
TTGGTGTTTTTATTGGTTTAAGACTTGGTGCTTTTTTGGTTGGGCTTTTATGTTTATATCA
GGTTGATATTAA

>st63_TDEC_ND4_13_[organism=*Triatoma dimidiata] Triatoma dimidiata* _ NADH DEHYDROGENASE SUBUNIT 4 (ND4) GENE

TTGACTTCCAAGGGCTCATGTTGAGGCGCCTATTGCTGGTTCAATGATTTTGGCTGGT
GTTCTTTTAAAGTTAGGGGGTGGTCTTATACGTGTTTCTAATTTTATTTATTTGTTTAAGT
TTGGTGTTTTTATTGGTTTAAGACTTGGTGCTTTTTTGGTTGGGCTTTTATGTTTATATCA
GGTTGATATTAA

>st64_TDEC_ND4_14_[organism=*Triatoma dimidiata] Triatoma dimidiata* _ NADH DEHYDROGENASE SUBUNIT 4 (ND4) GENE

TTGACTTCCAAGGGCTCATGTTGAGGCGCCTATTGCTGGTTCAATGATTTTGGCTGGT
GTTCTTTTAAAGTTAGGGGGTGGTCTTATACGTGTTTCTAATTTTATTTATTTGTTTAAGT
TTGGTGTTTTTATTGGTTTAAGACTTGGTGCTTTTTTGGTTGGGCTTTTATGTTTATATCA
GGTTGATATTAA

>st65_TDEC_ND4_15_[organism=*Triatoma dimidiata] Triatoma dimidiata* _ NADH DEHYDROGENASE SUBUNIT 4 (ND4) GENE

TTGACTTCCAAGGGCTCATGTTGAGGCGCCTATTGCTGGTTCAATGATTTTGGCTGGT
GTTCTTTTAAAGTTAGGGGGTGGTCTTATACGTGTTTCTAATTTTATTTATTTGTTTAAGT
TTGGTGTTTTTATTGGTTTAAGACTTGGTGCTTTTTTGGTTGGGCTTTTATGTTTATATCA
GGTTGATATTAA

>st66_TDEC_ND4_16_[organism=*Triatoma dimidiata] Triatoma dimidiata* _ NADH DEHYDROGENASE SUBUNIT 4 (ND4) GENE

TTGACTTCCAAGGGCTCATGTTGAGGCGCCTATTGCTGGTTCAATGATTTTGGCTGGT
GTTCTTTTAAAGTTAGGGGGTGGTCTTATACGTGTTTCTAATTTTATTTATTTGTTTAAGT
TTGGTGTTTTTATTGGTTTAAGACTTGGTGCTTTTTTGGTTGGGCTTTTATGTTTATATCA
GGTTGATATTAA

>st67_TDEC_ND4_17_[organism=*Triatoma dimidiata] Triatoma dimidiata* _ NADH DEHYDROGENASE SUBUNIT 4 (ND4) GENE

TTGACTTCCAAGGGCTCATGTTGAGGCGCCTATTGCTGGTTCAATGATTTTGGCTGGT
GTTCTTTTAAAGTTAGGGGGTGGTCTTATACGTGTTTCTAATTTTATTTATTTGTTTAAGT
TTGGTGTTTTTATTGGTTTAAGACTTGGTGCTTTTTTGGTTGGGCTTTTATGTTTATATCA
GGTTGATATTAA

>st68_TDEC_ND4_18_[organism=*Triatoma dimidiata] Triatoma dimidiata* _ NADH DEHYDROGENASE SUBUNIT 4 (ND4) GENE

TTGACTTCCAAGGGCTCATGTTGAGGCGCCTATTGCTGGTTCAATGATTTTGGCTGGT
GTTCTTTTAAAGTTAGGGGGTGGTCTTATACGTGTTTCTAATTTTATTTATTTGTTTAAGT
TTGGTGTTTTTATTGGTTTAAGACTTGGTGCTTTTTTGGTTGGGCTTTTATGTTTATATCA
GGTTGATATTAA

>st69_TDEC_ND4_19_[organism=*Triatoma dimidiata] Triatoma dimidiata* _ NADH DEHYDROGENASE SUBUNIT 4 (ND4) GENE

TTGACTTCCAAGGGCTCATGTTGAGGCGCCTATTGCTGGTTCAATGATTTTGGCTGGT
GTTCTTTTAAAGTTAGGGGGTGGTCTTATACGTGTTTCTAATTTTATTTATTTGTTTAAGT
TTGGTGTTTTTATTGGTTTAAGACTTGGTGCTTTTTTGGTTGGGCTTTTATGTTTATATCA
GGTTGATATTAA

>st70_TDEC_ND4_20_[organism=*Triatoma dimidiata] Triatoma dimidiata* _ NADH DEHYDROGENASE SUBUNIT 4 (ND4) GENE

TTGACTTCCAAGGGCTCATGTTGAGGCGCCTATTGCTGGTTCAATGATTTTGGCTGGT
GTTCTTTTAAAGTTAGGGGGTGGTCTTATACGTGTTTCTAATTTTATTTATTTGTTTAAGT
TTGGTGTTTTTATTGGTTTAAGACTTGGTGCTTTTTTGGTTGGGCTTTTATGTTTATATCA
GGTTGATATTAA

>st71_TDMR_ND4_1_[organism=*Triatoma dimidiata] Triatoma dimidiata* _ NADH DEHYDROGENASE SUBUNIT 4 (ND4) GENE

TTGACTTCCAAGGGCTCATGTTGAGGCGCCTATTGCTGGTTCAATGATTTTGGCTGGT
GTTCTTTTAAAGTTAGGGGGTGGTCTTATACGTGTTTCTAATTTTATTGGTTTGTTTAAGT
TTGGTGTTTTTATTGGTTTAAGACTTGGTGCTTTTTTAGTTGGGTTTTTATGTTTATATCA
GGTTGATATTAA

>st72_TDMR_ND4_2_[organism=*Triatoma dimidiata] Triatoma dimidiata* _ NADH DEHYDROGENASE SUBUNIT 4 (ND4) GENE

TTGACTTCCAAGGGCTCATGTTGAGGCGCCTATTGCTGGTTCAATGATTTTGGCTGGT
GTTCTTTTAAAGTTAGGGGGTGGTCTTATACGTGTTTCTAATTTTATTGGTTTGTTTAAGT
TTGGTGTTTTTATTGGTTTAAGACTTGGTGCTTTTTTAGTTGGGTTTTTATGTTTATATCA
GGTTGATATTAA

>st73_TDMR_ND4_3_[organism=*Triatoma dimidiata] Triatoma dimidiata* _ NADH DEHYDROGENASE SUBUNIT 4 (ND4) GENE

TTGACTTCCAAGGGCTCATGTTGAAGCGCCTATTGCTGGTTCAATGATTTTGGCTGGT
GTTCTTTTAAAGTTAGGGGGTGGTCTTATACGTGTTTCTAATTTTATTGGTTTGTTTAAGT
TTGGTGTTTTTATTGGTTTAAGACTTGGTGCTTTTTTAGTTGGGTTTTTATGTTTATATCA
GGTTGATATTAA

>st74_TDMR_ND4_4_[organism=*Triatoma dimidiata] Triatoma dimidiata* _ NADH DEHYDROGENASE SUBUNIT 4 (ND4) GENE

TTGACTTCCAAGGGCTCATGTTGAAGCGCCTATTGCTGGTTCAATGATTTTGGCTGGT
GTTCTTTTAAAGTTAGGGGGTGGTCTTATACGTGTTTCTAATTTTATTGGTTTGTTTAAGT
TTGGTGTTTTTATTGGTTTAAGACTTGGTGCTTTTTTAGTTGGGTTTTTATGTTTATATCA
GGTTGATATTAA

>st75_TDMR_ND4_5_[organism=*Triatoma dimidiata] Triatoma dimidiata* _ NADH DEHYDROGENASE SUBUNIT 4 (ND4) GENE

TTGACTTCCAAGGGCTCATGTTGAAGCGCCTATTGCTGGTTCAATGATTTTGGCTGGT
GTTCTTTTAAAGTTAGGGGGTGGTCTTATACGTGTTTCTAATTTTATTGGTTTGTTTAAGT
TTGGTGTTTTTATTGGTTTAAGACTTGGTGCTTTTTTAGTTGGGTTTTTATGTTTATATCA
GGTTGATATTAA

>st76_TDMR_ND4_6_[organism=*Triatoma dimidiata] Triatoma dimidiata* _ NADH DEHYDROGENASE SUBUNIT 4 (ND4) GENE

TTGACTTCCAAGGGCTCATGTTGAAGCGCCTATTGCTGGTTCAATGATTTTGGCTGGT
GTTCTTTTAAAGTTAGGGGGTGGTCTTATACGTGTTTCTAATTTTATTGGTTTGTTTAAGT
TTGGTGTTTTTATTGGTTTAAGACTTGGTGCTTTTTTAGTTGGGTTTTTATGTTTATATCA
GGTTGATATTAA

>st77_TDMR_ND4_7_[organism=*Triatoma dimidiata] Triatoma dimidiata* _ NADH DEHYDROGENASE SUBUNIT 4 (ND4) GENE

TTGACTTCCAAGGGCTCATGTTGAGGCGCCTATTGCTGGTTCAATGATTTTGGCTGGT
GTTCTTTTAAAGTTAGGGGGTGGTCTTATACGTGTTTCTAATTTTATTGGTTTGTTTAAGT
TTGGTGTTTTTATTGGTTTAAGACTTGGTGCTTTTTTAGTTGGGTTTTTATGTTTATATCA
GGTTGATATTAA

>st78_TDMR_ND4_8_[organism=*Triatoma dimidiata] Triatoma dimidiata* _ NADH DEHYDROGENASE SUBUNIT 4 (ND4) GENE

TTGACTTCCAAGGGCTCATGTTGAAGCGCCTATTGCTGGTTCAATGATTTTGGCTGGT
GTTCTTTTAAAGTTAGGGGGTGGTCTTATACGTGTTTCTAATTTTATTGGTTTGTTTAAGT
TTGGTGTTTTTATTGGTTTAAGACTTGGTGCTTTTTTAGTTGGGTTTTTATGTTTATATCA
GGTTGATATTAA

>st79_TDMR_ND4_9_[organism=*Triatoma dimidiata] Triatoma dimidiata* _ NADH DEHYDROGENASE SUBUNIT 4 (ND4) GENE

TTGACTTCCAAGGGCTCATGTTGAGGCGCCTATTGCTGGTTCAATGATTTTGGCTGGT
GTTCTTTTAAAGTTAGGGGGTGGTCTTATACGTGTTTCTAATTTTATTGGTTTGTTTAAGT
TTGGTGTTTTTATTGGTTTAAGACTTGGTGCTTTTTTAGTTGGGTTTTTATGTTTATATCA
GGTTGATATTAA

>st80_TDMR_ND4_10_[organism=*Triatoma dimidiata] Triatoma dimidiata* _ NADH DEHYDROGENASE SUBUNIT 4 (ND4) GENE

TTGACTTCCAAGGGCTCATGTTGAAGCGCCTATTGCTGGTTCAATGATTTTGGCTGGT
GTTCTTTTAAAGTTAGGGGGTGGTCTTATACGTGTTTCTAATTTTATTGGTTTGTTTAAGT

TTGGTGTTTTTATTGGTTTAAGACTTGGTGCTTTTTTAGTTGGGTTTTTATGTTTATATCA
GGTTGATATTAA

>st71_TDMR_ND4_11_[organism=*Triatoma dimidiata] Triatoma dimidiata* _ NADH DEHYDROGENASE SUBUNIT 4 (ND4) GENE

TTGACTTCCAAGGGCTCATGTTGAGGCGCCTATTGCTGGTTCAATGATTTTGGCTGGT
GTTCTTTTAAAGTTAGGGGGTGGTCTTATACGTGTTTCTAATATTTATGGTTTGTTTAAGT
TTGGTGTTTTTATTGGTTTAAGACATGGTGCTTTTTTAGTTGGGTTTTTATGTTTATATCA
GGTTGATATTAA

>st72_TDMR_ND4_12_[organism=*Triatoma dimidiata] Triatoma dimidiata* _ NADH DEHYDROGENASE SUBUNIT 4 (ND4) GENE

TTGACTTCCAAGGGCTCATGTTGAGGCGCCTATTGCTGGTTCAATGATTTTGGCTGGT
GTTCTTTTAAAGTTAGGGGGTGGTCTTATACGTGTTTCTAATATTTATGGTTTGTTTAAGT
TTGGTGTTTTTATTGGTTTAAGACATGGTGCTTTTTTAGTTGGGTTTTTATGTTTATATCA
GGTTGATATTAA

>st73_TDMR_ND4_13_[organism=*Triatoma dimidiata] Triatoma dimidiata* _ NADH DEHYDROGENASE SUBUNIT 4 (ND4) GENE

TTGACTTCCAAGGGCTCATGTTGAGGCGCCTATTGCTGGTTCAATGATTTTGGCTGGT
GTTCTTTTAAAGTTAGGGGGTGGTCTTATACGTGTTTCTAATATTTATGGTTTGTTTAAGT
TTGGTGTTTTTATTGGTTTAAGACATGGTGCTTTTTTAGTTGGGTTTTTATGTTTATATCA
GGTTGATATTAA

>st74_TDMR_ND4_14_[organism=*Triatoma dimidiata] Triatoma dimidiata* _ NADH DEHYDROGENASE SUBUNIT 4 (ND4) GENE

TTGACTTCCAAGGGCTCATGTTGAAGCGCCTATTGCTGGTTCAATGATTTTGGCTGGT
GTTCTTTTAAAGTTAGGGGGTGGTCTTATACGTGTTTCTAATATTTATGGTTTGTTTAAGT
TTGGTGTTTTTATTGGTTTAAGACATGGTGCTTTTTTAGTTGGGTTTTTATGTTTATATCA
GGTTGATATTAA

>st75_TDMR_ND4_15_[organism=*Triatoma dimidiata] Triatoma dimidiata* _ NADH DEHYDROGENASE SUBUNIT 4 (ND4) GENE

TTGACTTCCAAGGGCTCATGTTGAAGCGCCTATTGCTGGTTCAATGATTTTGGCTGGT
GTTCTTTTAAAGTTAGGGGGTGGTCTTATACGTGTTTCTAATATTTATGGTTTGTTTAAGT
TTGGTGTTTTTATTGGTTTAAGACATGGTGCTTTTTTAGTTGGGTTTTTATGTTTATATCA
GGTTGATATTAA

>st76_TDMR_ND4_16_[organism=*Triatoma dimidiata] Triatoma dimidiata* _ NADH DEHYDROGENASE SUBUNIT 4 (ND4) GENE

TTGACTTCCAAGGGCTCATGTTGAGGCGCCTATTGCTGGTTCAATGATTTTGGCTGGT
GTTCTTTTAAAGTTAGGGGGTGGTCTTATACGTGTTTCTAATATTTATGGTTTGTTTAAGT
TTGGTGTTTTTATTGGTTTAAGACATGGTGCTTTTTTAGTTGGGTTTTTATGTTTATATCA
GGTTGATATTAA

>st77_TDMR_ND4_17_[organism=*Triatoma dimidiata] Triatoma dimidiata* _ NADH DEHYDROGENASE SUBUNIT 4 (ND4) GENE

TTGACTTCCAAGGGCTCATGTTGAAGCGCCTATTGCTGGTTCAATGATTTTGGCTGGT
GTTCTTTTAAAGTTAGGGGGTGGTCTTATACGTGTTTCTAATATTTATGGTTTGTTTAAGT
TTGGTGTTTTTATTGGTTTAAGACATGGTGCTTTTTTAGTTGGGTTTTTATGTTTATATCA
GGTTGATATTAA

>st78_TDMR_ND4_18_[organism=*Triatoma dimidiata] Triatoma dimidiata* _ NADH DEHYDROGENASE SUBUNIT 4 (ND4) GENE

TTGACTTCCAAGGGCTCATGTTGAGGCGCCTATTGCTGGTTCAATGATTTTGGCTGGT
GTTCTTTTAAAGTTAGGGGGTGGTCTTATACGTGTTTCTAATATTTATGGTTTGTTTAAGT
TTGGTGTTTTTATTGGTTTAAGACATGGTGCTTTTTTAGTTGGGTTTTTATGTTTATATCA
GGTTGATATTAA

>st79_TDMR_ND4_19_[organism=*Triatoma dimidiata] Triatoma dimidiata* _ NADH DEHYDROGENASE SUBUNIT 4 (ND4) GENE

TTGACTTCCAAGGGCTCATGTTGAAGCGCCTATTGCTGGTTCAATGATTTTGGCTGGT
GTTCTTTTAAAGTTAGGGGGTGGTCTTATACGTGTTTCTAATATTTATGGTTTGTTTAAGT
TTGGTGTTTTTATTGGTTTAAGACATGGTGCTTTTTTAGTTGGGTTTTTATGTTTATATCA
GGTTGATATTAA

>st80_TDMR_ND4_20_[organism=*Triatoma dimidiata] Triatoma dimidiata* _ NADH DEHYDROGENASE SUBUNIT 4 (ND4) GENE

TTGACTTCCAAGGGCTCATGTTGAAGCGCCTATTGCTGGTTCAATGATTTTGGCTGGT
GTTCTTTTAAAGTTAGGGGGTGGTCTTATACGTGTTTCTAATATTTATGGTTTGTTTAAGT
TTGGTGTTTTTATTGGTTTAAGACATGGTGCTTTTTTAGTTGGGTTTTTATGTTTATATCA
GGTTGATATTAA
