# Supplementary material for: First Report of the L925I kdr Mutation Associated with Pyrethroid Resistance in Genetically Distinct Triatoma dimidiata, Vector of Chagas Disease in Mexico
Source: Trop Med Infect Dis. 2025 Jun 27;10(7):182. doi: 10.3390/tropicalmed10070182 (PMC12299954; doi:10.3390/tropicalmed10070182)
Supplement: Supplementary file 1 [file tropicalmed-10-00182-s001.zip › File S2. Sequences_cytb_fragment.pdf]

>st1_TDYCH_CytB_1_[organism=Triatoma dimidiata] Triatoma dimidiata cytochrome b (cytb) gene

GCTATAGTAATAATCCATCTCCTATTTTTACATCAAACAGGATCCAGAAATCCATTAGGG

TTAAACAGAAACTTTGACAAAATCCCATTCCACCCATATTTCTCCATTAAAGACCTGGTA

GGAGTATCCTTAACTCTAATATTCTTTATCCTCTTAAGTCTATGAGAAGCTCCAATTTTA

ATAGACCCAGAAAACTTTATCCCTGCAAACCCACTAGTTACACCAGTACACATTCAACC
A

GAATGATACTTCCTATT

>st2_TDYCH_CytB_2_[organism=Triatoma dimidiata] Triatoma dimidiata cytochrome b (cytb) gene

GCTATGGTAATAATCCACCTCCTATTTTTACATCAAACAGGATCTAGAAATCCGTTAGGA

TTAAACAGAAACTTTGACAAGATCCCATTCCATCCATATTTTTCAATTAAAGACCTAATA

GGAGTATCCTTAACCTTAATATTCTTTATCCTCCTAAGTCTATGGGAAGCCCCAATTTTA

ATAGACCCAGAAAACTTTATTCCCGCAAACCCACTAGTTACACCAGTACATATTCAACCA

GAATGATACTTCCTATT

>st3_TDYCH_CytB_3_[organism=Triatoma dimidiata] Triatoma dimidiata cytochrome b (cytb) gene

GCTATGGTAATAATCCACCTCCTATTTTTACATCAAACAGGATCTAGAAATCCGTTAGGA

TTAAACAGAAACTTTGACAAGATCCCATTCCATCCATATTTTTCAATTAAAGACCTAATA

GGAGTATCCTTAACCTTAATATTCTTTATCCTCCTAAGTCTATGGGAAGCCCCAATTTTA

ATAGACCCAGAAAACTTTATTCCCGCAAACCCACTAGTTACACCAGTACATATTCAACCA

GAATGATACTTCCTATT

>st4_TDYCH_CytB_4_[organism=Triatoma dimidiata] Triatoma dimidiata cytochrome b (cytb) gene

GCTATAGTAATAATCCATCTCCTATTTTTACATCAAACAGGATCCAGAAATCCATTAGGG

TTAAACAGAAACTTTGACAAAATCCCATTCCACCCATATTTCTCCATTAAAGACCTGGTA

GGAGTATCCTTAACTCTAATATTCTTTATCCTCTTAAGTCTATGAGAAGCTCCAATTTTA

ATAGACCCAGAAAACTTTATCCCTGCAAACCCACTAGTTACACCAGTACACATTCAACC
A

GAATGATACTTCCTATT

>st5_TDYCH_CytB_5_[organism=Triatoma dimidiata] Triatoma dimidiata cytochrome b (cytb) gene

GCTATAGTAATAATCCATCTCCTATTTTTACATCAAACAGGATCCAGAAATCCATTAGGG

TTAAACAGAAACTTTGACAAAATCCCATTCCACCCATATTTCTCCATTAAAGACCTGGTA

GGAGTATCCTTAACTCTAATATTCTTTATCCTCTTAAGTCTATGAGAAGCTCCAATTTTA

ATAGACCCAGAAAACTTTATCCCTGCAAACCCACTAGTTACACCAGTACACATTCAACC
A

GAATGATACTTCCTATT

>st6_TDYCH_CytB_6_[organism=Triatoma dimidiata] Triatoma dimidiata cytochrome b (cytb) gene

GCTATAGTAATAATCCATCTCCTATTTTTACATCAAACAGGATCCAGAAATCCATTAGGG

TTAAACAGAAACTTTGACAAAATCCCATTCCACCCATATTTCTCCATTAAAGACCTGGTA

GGAGTATCCTTAACTCTAATATTCTTTATCCTCTTAAGTCTATGAGAAGCTCCAATTTTA

ATAGACCCAGAAAACTTTATCCCTGCAAACCCACTAGTTACACCAGTACACATTCAACC
A

GAATGATACTTCCTATT

>st9_TDYCH_CytB_9_[organism=Triatoma dimidiata] Triatoma dimidiata cytochrome b (cytb) gene

GCTATAGTAATAATCCATCTCCTATTTTTACATCAAACAGGATCCAGAAATCCATTAGGG

TTAAACAGAAACTTTGACAAAATCCCATTCCACCCATATTTCTCCATTAAAGACCTGGTA

GGAGTATCCTTAACTCTAATATTCTTTATCCTCTTAAGTCTATGAGAAGCTCCAATTTTA

ATAGACCCAGAAAACTTTATCCCTGCAAACCCACTAGTTACACCAGTACACATTCAACC
A

GAATGATACTTCCTATT

>st1_TDYCH_CytB_11_[organism=Triatoma dimidiata] Triatoma dimidiata cytochrome b (cytb) gene

GCTATAGTAATAATCCATCTCCTATTTTTACATCAAACAGGATCCAGAAATCCATTAGGG

TTAAACAGAAACTTTGACAAAATCCCATTCCACCCATATTTCTCCATTAAAGACCTGGTA

GGAGTATCCTTAACTCTAATATTCTTTATCCTCTTAAGTCTATGAGAAGCTCCAATTTTA

ATAGACCCAGAAAACTTTATCCCTGCAAACCCACTAGTTACACCAGTACACATTCAACC
A

GAATGATACTTCCTATT

>st2_TDYCH_CytB_12_[organism=Triatoma dimidiata] Triatoma dimidiata cytochrome b (cytb) gene

GCTATAGTAATAATCCATCTCCTATTTTTACATCAAACAGGATCCAGAAATCCATTAGGG

TTAAACAGAAACTTTGACAAAATCCCATTCCACCCATATTTCTCCATTAAAGACCTGGTA

GGAGTATCCTTAACTCTAATATTCTTTATCCTCTTAAGTCTATGAGAAGCTCCAATTTTA

ATAGACCCAGAAAACTTTATCCCTGCAAACCCACTAGTTACACCAGTACACATTCAACC
A

GAATGATACTTCCTATT

>st3_TDYCH_CytB_13_[organism=Triatoma dimidiata] Triatoma dimidiata cytochrome b (cytb) gene

GCTATAGTAATAATCCATCTCCTATTTTTACATCAAACAGGATCCAGAAATCCATTAGGG

TTAAACAGAAACTTTGACAAAATCCCATTCCACCCATATTTCTCCATTAAAGACCTGGTA

GGAGTATCCTTAACTCTAATATTCTTTATCCTCTTAAGTCTATGAGAAGCTCCAATTTTA

ATAGACCCAGAAAACTTTATCCCTGCAAACCCACTAGTTACACCAGTACACATTCAACC
A

GAATGATACTTCCTATT

>st4_TDYCH_CytB_14_[organism=Triatoma dimidiata] Triatoma dimidiata cytochrome b (cytb) gene

GCTATAGTAATAATCCATCTCCTATTTTTACATCAAACAGGATCCAGAAATCCATTAGGG

TTAAACAGAAACTTTGACAAAATCCCATTCCACCCATATTTCTCCATTAAAGACCTGGTA

GGAGTATCCTTAACTCTAATATTCTTTATCCTCTTAAGTCTATGAGAAGCTCCAATTTTA

ATAGACCCAGAAAACTTTATCCCTGCAAACCCACTAGTTACACCAGTACACATTCAACC
A

GAATGATACTTCCTATT

>st5_TDYCH_CytB_15_[organism=Triatoma dimidiata] Triatoma dimidiata cytochrome b (cytb) gene

GCTATAGTAATAATCCATCTCCTATTTTTACATCAAACAGGATCCAGAAATCCATTAGGG

TTAAACAGAAACTTTGACAAAATCCCATTCCACCCATATTTCTCCATTAAAGACCTGGTA

GGAGTATCCTTAACTCTAATATTCTTTATCCTCTTAAGTCTATGAGAAGCTCCAATTTTA

ATAGACCCAGAAAACTTTATCCCTGCAAACCCACTAGTTACACCAGTACACATTCAACC
A

GAATGATACTTCCTATT

>st6_TDYCH_CytB_16_[organism=Triatoma dimidiata] Triatoma dimidiata cytochrome b (cytb) gene

GCTATAGTAATAATCCATCTCCTATTTTTACATCAAACAGGATCCAGAAATCCATTAGGG

TTAAACAGAAACTTTGACAAAATCCCATTCCACCCATATTTCTCCATTAAAGACCTGGTA

GGAGTATCCTTAACTCTAATATTCTTTATCCTCTTAAGTCTATGAGAAGCTCCAATTTTA

ATAGACCCAGAAAACTTTATCCCTGCAAACCCACTAGTTACACCAGTACACATTCAACC
A

GAATGATACTTCCTATT

>st7_TDYCH_CytB_17_[organism=Triatoma dimidiata] Triatoma dimidiata cytochrome b (cytb) gene

GCTATAGTAATAATCCATCTCCTATTTTTACATCAAACAGGATCCAGAAATCCATTAGGG

TTAAACAGAAACTTTGACAAAATCCCATTCCACCCATATTTCTCCATTAAAGACCTGGTA

GGAGTATCCTTAACTCTAATATTCTTTATCCTCTTAAGTCTATGAGAAGCTCCAATTTTA

ATAGACCCAGAAAACTTTATCCCTGCAAACCCACTAGTTACACCAGTACACATTCAACC
A

GAATGATACTTCCTATT

>st8_TDYCH_CytB_18_[organism=Triatoma dimidiata] Triatoma dimidiata cytochrome b (cytb) gene

GCTATAGTAATAATCCATCTCCTATTTTTACATCAAACAGGATCCAGAAATCCATTAGGG

TTAAACAGAAACTTTGACAAAATCCCATTCCACCCATATTTCTCCATTAAAGACCTGGTA

GGAGTATCCTTAACTCTAATATTCTTTATCCTCTTAAGTCTATGAGAAGCTCCAATTTTA

ATAGACCCAGAAAACTTTATCCCTGCAAACCCACTAGTTACACCAGTACACATTCAACC
A

GAATGATACTTCCTATT

>st9_TDYCH_CytB_19_[organism=Triatoma dimidiata] Triatoma dimidiata cytochrome b (cytb) gene

GCTATAGTAATAATCCATCTCCTATTTTTACATCAAACAGGATCCAGAAATCCATTAGGG

TTAAACAGAAACTTTGACAAAATCCCATTCCACCCATATTTCTCCATTAAAGACCTGGTA

GGAGTATCCTTAACTCTAATATTCTTTATCCTCTTAAGTCTATGAGAAGCTCCAATTTTA

ATAGACCCAGAAAACTTTATCCCTGCAAACCCACTAGTTACACCAGTACACATTCAACC
A

GAATGATACTTCCTATT

>st10_TDYCH_CytB_20_[organism=Triatoma dimidiata] Triatoma dimidiata cytochrome b (cytb) gene

GCTATAGTAATAATCCATCTCCTATTTTTACATCAAACAGGATCCAGAAATCCATTAGGG

TTAAACAGAAACTTTGACAAAATCCCATTCCACCCATATTTCTCCATTAAAGACCTGGTA

GGAGTATCCTTAACTCTAATATTCTTTATCCTCTTAAGTCTATGAGAAGCTCCAATTTTA

ATAGACCCAGAAAACTTTATCCCTGCAAACCCACTAGTTACACCAGTACACATTCAACC
A

GAATGATACTTCCTATT

>st11_TDYCK_CytB_1_[organism=Triatoma dimidiata] Triatoma dimidiata cytochrome b (cytb) gene

GCCATAGGAATAATCCATCTCCTATTTTTACATCAAACAGGATCCAGAAATCCATTAGGG

TTAAACAGAAACTTTGACAAAATCCCATTTCACCCATATTTCTCCATTAAAGACCTGATA

GGAGTATCTTTAACTCTAATATTCTTTATCCTCCTAAGTCTATGAGAAGCTCCAATTTTA

ATAGACCCGGAAAACTTTATCCCTGCAAACCCACTAGTTACACCAGTACACATTCAACC
A

GAATGATACTTCCTATT

>st12_TDYCK_CytB_2_[organism=Triatoma dimidiata] Triatoma dimidiata cytochrome b (cytb) gene

GCTATAGGAATAATCCATCTCCTATTTTTACATCAAACAGGATCCAGAAATCCATTAGGG

TTAAACAGAAACTTTGACAAAATCCCATTTCACCCATATTTCTCCATTAAAGACCTGATA

GGAGTATCTTTAACTCTAATATTCTTTATCCTCCTAAGTCTATGAGAAGCTCCAATTTTA

ATAGACCCGGAAAACTTTATCCCTGCAAACCCACTAGTTACACCAGTACACATTCAACC
A

GAATGATACTTCCTATT

>st13_TDYCK_CytB_3_[organism=Triatoma dimidiata] Triatoma dimidiata cytochrome b (cytb) gene

GCTATAGGAATAATCCATCTCCTATTTTTACATCAAACAGGATCCAGAAATCCATTAGGG

TTAAACAGAAACTTTGACAAAATCCCATTTCACCCATATTTCTCCATTAAAGACCTGATA

GGAGTATCTTTAACTCTAATATTCTTTATCCTCCTAAGTCTATGAGAAGCTCCAATTTTA

ATAGACCCGGAAAACTTTATCCCTGCAAACCCACTAGTTACACCAGTACACATTCAACC
A

GAATGATACTTCCTATT

>st14_TDYCK_CytB_4_[organism=Triatoma dimidiata] Triatoma dimidiata cytochrome b (cytb) gene

GCTATAGGAATAATCCATCTCCTATTTTTACATCAAACAGGATCCAGAAATCCATTAGGG

TTAAACAGAAACTTTGACAAAATCCCATTTCACCCATATTTCTCCATTAAAGACCTGATA

GGAGTATCTTTAACTCTAATATTCTTTATCCTCCTAAGTCTATGAGAAGCTCCAATTTTA

ATAGACCCGGAAAACTTTATCCCTGCAAACCCACTAGTTACACCAGTACACATTCAACC
A

GAATGATACTTCCTATT

>st15_TDYCK_CytB_5_[organism=Triatoma dimidiata] Triatoma dimidiata cytochrome b (cytb) gene

GCTATAGGAATAATCCATCTCCTATTTTTACATCAAACAGGATCCAGAAATCCATTAGGG

TTAAACAGAAACTTTGACAAAATCCCATTTCACCCATATTTCTCCATTAAAGACCTGATA

GGAGTATCTTTAACTCTAATATTCTTTATCCTCCTAAGTCTATGAGAAGCTCCAATTTTA

ATAGACCCGGAAAACTTTATCCCTGCAAACCCACTAGTTACACCAGTACACATTCAACC
A

GAATGATACTTCCTATT

>st16_TDYCK_CytB_6_[organism=Triatoma dimidiata] Triatoma dimidiata cytochrome b (cytb) gene

GCTATAGGAATAATCCATCTCCTATTTTTACATCAAACAGGATCCAGAAATCCATTAGGG

TTAAACAGAAACTTTGACAAAATCCCATTTCACCCATATTTCTCCATTAAAGACCTGATA

GGAGTATCTTTAACTCTAATATTCTTTATCCTCCTAAGTCTATGAGAAGCTCCAATTTTA

ATAGACCCGGAAAACTTTATCCCTGCAAACCCACTAGTTACACCAGTACACATTCAACC
A

GAATGATACTTCCTATT

>st17_TDYCK_CytB_7_[organism=Triatoma dimidiata] Triatoma dimidiata cytochrome b (cytb) gene

GCTATAGGAATAATCCATCTCCTATTTTTACATCAAACAGGATCCAGAAATCCATTAGGG

TTAAACAGAAACTTTGACAAAATCCCATTTCACCCATATTTCTCCATTAAAGACCTGATA

GGAGTATCTTTAACTCTAATATTCTTTATCCTCCTAAGTCTATGAGAAGCTCCAATTTTA

ATAGACCCGGAAAACTTTATCCCTGCAAACCCACTAGTTACACCAGTACACATTCAACC
A

GAATGATACTTCCTATT

>st18_TDYCK_CytB_8_[organism=Triatoma dimidiata] Triatoma dimidiata cytochrome b (cytb) gene

GCTATAGGAATAATCCATCTCCTATTTTTACATCAAACAGGATCCAGAAATCCATTAGGG

TTAAACAGAAACTTTGACAAAATCCCATTTCACCCATATTTCTCCATTAAAGACCTGATA

GGAGTATCTTTAACTCTAATATTCTTTATCCTCCTAAGTCTATGAGAAGCTCCAATTTTA

ATAGACCCGGAAAACTTTATCCCTGCAAACCCACTAGTTACACCAGTACACATTCAACC
A

GAATGATACTTCCTATT

>st19_TDYCK_CytB_9_[organism=Triatoma dimidiata] Triatoma dimidiata cytochrome b (cytb) gene

GCTATAGTAATAATCCATCTCCTATTTTTACATCAAACAGGATCCAGAAATCCATTAGGG

TTAAACAGAAACTTTGACAAAATCCCATTTCACCCATATTTCTCCATTAAAGACCTGATA

GGAGTATCTTTAACTCTAATATTCTTTATCCTCCTAAGTCTATGAGAAGCTCCAATTTTA

ATAGACCCGGAAAACTTTATCCCTGCAAACCCACTAGTTACACCAGTACACATTCAACC
A

GAATGATACTTCCTATT

>st20_TDYCK_CytB_10_[organism=Triatoma dimidiata] Triatoma dimidiata cytochrome b (cytb) gene

GCTATAGTAATAATCCATCTCCTATTTTTACATCAAACAGGATCCAGAAATCCATTAGGG

TTAAACAGAAACTTTGACAAAATCCCATTTCACCCATATTTCTCCATTAAAGACCTGATA

GGAGTATCTTTAACTCTAATATTCTTTATCCTCCTAAGTCTATGAGAAGCTCCAATTTTA

ATAGACCCGGAAAACTTTATCCCTGCAAACCCACTAGTTACACCAGTACACATTCAACC
A

GAATGATACTTCCTATT

>st11_TDYCK_CytB_11_[organism=Triatoma dimidiata] Triatoma dimidiata cytochrome b (cytb) gene

GCTATAGTAATAATCCATCTCCTATTTTTACATCAAACAGGATCCAGAAATCCATTAGGG

TTAAACAGAAACTTTGACAAAATCCCATTCCACCCATATTTCTCCATTAAAGACCTGGTA

GGAGTATCCTTAACTCTAATATTCTTTATCCTCTTAAGTCTATGAGAAGCTCCAATTTTA

ATAGACCCAGAAAACTTTATCCCTGCAAACCCACTAGTTACACCAGTACACATTCAACC
A

GAATGATACTTCCTATT

>st12_TDYCK_CytB_12_[organism=Triatoma dimidiata] Triatoma dimidiata cytochrome b (cytb) gene

GCTATAGTAATAATCCATCTCCTATTTTTACATCAAACAGGATCCAGAAATCCATTAGGG

TTAAACAGAAACTTTGACAAAATCCCATTCCACCCATATTTCTCCATTAAAGACCTGGTA

GGAGTATCCTTAACTCTAATATTCTTTATCCTCTTAAGTCTATGAGAAGCTCCAATTTTA

ATAGACCCAGAAAACTTTATCCCTGCAAACCCACTAGTTACACCAGTACACATTCAACC
A

GAATGATACTTCCTATT

>st13_TDYCK_CytB_13_[organism=Triatoma dimidiata] Triatoma dimidiata cytochrome b (cytb) gene

GCTATAGTAATAATCCATCTCCTATTTTTACATCAAACAGGATCCAGAAATCCATTAGGG

TTAAACAGAAACTTTGACAAAATCCCATTCCACCCATATTTCTCCATTAAAGACCTGGTA

GGAGTATCCTTAACTCTAATATTCTTTATCCTCTTAAGTCTATGAGAAGCTCCAATTTTA

ATAGACCCAGAAAACTTTATCCCTGCAAACCCACTAGTTACACCAGTACACATTCAACC
A

GAATGATACTTCCTATT

>st14_TDYCK_CytB_14_[organism=Triatoma dimidiata] Triatoma dimidiata cytochrome b (cytb) gene

GCTATAGTAATAATCCATCTCCTATTTTTACATCAAACAGGATCCAGAAATCCATTAGGG

TTAAACAGAAACTTTGACAAAATCCCATTCCACCCATATTTCTCCATTAAAGACCTGGTA

GGAGTATCCTTAACTCTAATATTCTTTATCCTCTTAAGTCTATGAGAAGCTCCAATTTTA

ATAGACCCAGAAAACTTTATCCCTGCAAACCCACTAGTTACACCAGTACACATTCAACC
A

GAATGATACTTCCTATT

>st15_TDYCK_CytB_15_[organism=Triatoma dimidiata] Triatoma dimidiata cytochrome b (cytb) gene

GCTATAGTAATAATCCATCTCCTATTTTTACATCAAACAGGATCCAGAAATCCATTAGGG

TTAAACAGAAACTTTGACAAAATCCCATTCCACCCATATTTCTCCATTAAAGACCTGGTA

GGAGTATCCTTAACTCTAATATTCTTTATCCTCTTAAGTCTATGAGAAGCTCCAATTTTA

ATAGACCCAGAAAACTTTATCCCTGCAAACCCACTAGTTACACCAGTACACATTCAACC
A

GAATGATACTTCCTATT

>st16_TDYCK_CytB_16_[organism=Triatoma dimidiata] Triatoma dimidiata cytochrome b (cytb) gene

GCTATAGTAATAATCCATCTCCTATTTTTACATCAAACAGGATCCAGAAATCCATTAGGG

TTAAACAGAAACTTTGACAAAATCCCATTCCACCCATATTTCTCCATTAAAGACCTGGTA

GGAGTATCCTTAACTCTAATATTCTTTATCCTCTTAAGTCTATGAGAAGCTCCAATTTTA

ATAGACCCAGAAAACTTTATCCCTGCAAACCCACTAGTTACACCAGTACACATTCAACC
A

GAATGATACTTCCTATT

>st17_TDYCK_CytB_17_[organism=Triatoma dimidiata] Triatoma dimidiata cytochrome b (cytb) gene

GCTATAGTAATAATCCATCTCCTATTTTTACATCAAACAGGATCCAGAAATCCATTAGGG

TTAAACAGAAACTTTGACAAAATCCCATTCCACCCATATTTCTCCATTAAAGACCTGGTA

GGAGTATCCTTAACTCTAATATTCTTTATCCTCTTAAGTCTATGAGAAGCTCCAATTTTA

ATAGACCCAGAAAACTTTATCCCTGCAAACCCACTAGTTACACCAGTACACATTCAACC
A

GAATGATACTTCCTATT

>st18_TDYCK_CytB_18_[organism=Triatoma dimidiata] Triatoma dimidiata cytochrome b (cytb) gene

GCTATAGGAATAATCCATCTCCTATTTTTACATCAAACAGGATCCAGAAATCCATTAGGG

TTAAACAGAAACTTTGACAAAATCCCATTTCACCCATATTTCTCCATTAAAGACCTGATA

GGAGTATCTTTAACTCTAATATTCTTTATCCTCTTAAGTCTATGAGAAGCTCCAATTTTA

ATAGACCCGGAAAACTTTATCCCTGCAAACCCACTAGTTACACCAGTACACATTCAACC
A

GAATGATACTTCCTATT

>st19_TDYCK_CytB_19_[organism=Triatoma dimidiata] Triatoma dimidiata cytochrome b (cytb) gene

GCTATAGTAATAATCCATCTCCTATTTTTACATCAAACAGGATCCAGAAATCCATTAGGG

TTAAACAGAAACTTTGACAAAATCCCATTCCACCCATATTTCTCCATTAAAGACCTGGTA

GGAGTATCCTTAACTCTAATATTCTTTATCCTCTTAAGTCTATGAGAAGCTCCAATTTTA

ATAGACCCAGAAAACTTTATCCCTGCAAACCCACTAGTTACACCAGTACACATTCAACC
A

GAATGATACTTCCTATT

>st20_TDYCK_CytB_20_[organism=Triatoma dimidiata] Triatoma dimidiata cytochrome b (cytb) gene

GCTATAGGAATAATCCATCTCCTATTTTTACATCAAACAGGATCCAGAAATCCATTAGGG

TTAAACAGAAACTTTGACAAAATCCCATTTCACCCATATTTCTCCATTAAAGACCTGATA

GGAGTATCTTTAACTCTAATATTCTTTATCCTCTTAAGTCTATGAGAAGCTCCAATTTTA

ATAGACCCGGAAAACTTTATCCCTGCAAACCCACTAGTTACACCAGTACACATTCAACC
A

GAATGATACTTCCTATT

>st21_TDEC_CytB_1_[organism=Triatoma dimidiata] Triatoma dimidiata cytochrome b (cytb) gene

GCTATGGTAATAATCCACCTCCTATTTTTACATCAAACAGGATCTAGAAATCCGTTAGGA

TTAAACAGAAACTTTGACAAGATCCCATTCCATCCATATTTTTCAATTAAAGACCTAATA

GGAGTATCCTTAACCTTAATATTCTTTATCCTCCTAAGTCTATGGGAAGCCCCAATTTTA

ATAGACCCAGAAAACTTTATTCCCGCAAACCCACTAGTTACACCAGTACATATTCAACCA

GAATGATACTTCCTATT

>st22_TDEC_CytB_2_[organism=Triatoma dimidiata] Triatoma dimidiata cytochrome b (cytb) gene

GCTATGGTAATAATCCACCTCCTATTTTTACATCAAACAGGATCTAGAAATCCGTTAGGA

TTAAACAGAAACTTTGACAAGATCCCATTCCATCCATATTTTTCAATTAAAGACCTAATA

GGAGTATCCTTAACCTTAATATTCTTTATCCTCCTAAGTCTATGGGAAGCCCCAATTTTA

ATAGACCCAGAAAACTTTATTCCCGCAAACCCACTAGTTACACCAGTACATATTCAACCA

GAATGATACTTCCTATT

>st23_TDEC_CytB_3_[organism=Triatoma dimidiata] Triatoma dimidiata cytochrome b (cytb) gene

GCTATGGTAATAATCCACCTCCTATTTTTACATCAAACAGGATCTAGAAATCCGTTAGGA

TTAAACAGAAACTTTGACAAGATCCCATTCCATCCATATTTTTCAATTAAAGACCTAATA

GGAGTATCCTTAACCTTAATATTCTTTATCCTCCTAAGTCTATGGGAAGCCCCAATTTTA

ATAGACCCAGAAAACTTTATTCCCGCAAACCCACTAGTTACACCAGTACATATTCAACCA

GAATGATACTTCCTATT

>st24_TDEC_CytB_4_[organism=Triatoma dimidiata] Triatoma dimidiata cytochrome b (cytb) gene

GCTATGGTAATAATCCACCTCCTATTTTTACATCAAACAGGATCTAGAAATCCGTTAGGA

TTAAACAGAAACTTTGACAAGATCCCATTCCATCCATATTTTTCAATTAAAGACCTAATA

GGAGTATCCTTAACCTTAATATTCTTTATCCTCCTAAGTCTATGGGAAGCCCCAATTTTA

ATAGACCCAGAAAACTTTATTCCCGCAAACCCACTAGTTACACCAGTACATATTCAACCA

GAATGATACTTCCTATT

>st25_TDEC_CytB_5_[organism=Triatoma dimidiata] Triatoma dimidiata cytochrome b (cytb) gene

GCTATGGTAATAATCCACCTCCTATTTTTACATCAAACAGGATCTAGAAATCCGTTAGGA

TTAAACAGAAACTTTGACAAGATCCCATTCCATCCATATTTTTCAATTAAAGACCTAATA

GGAGTATCCTTAACCTTAATATTCTTTATCCTCCTAAGTCTATGGGAAGCCCCAATTTTA

ATAGACCCAGAAAACTTTATTCCCGCAAACCCACTAGTTACACCAGTACATATTCAACCA

GAATGATACTTCCTATT

>st26_TDEC_CytB_6_[organism=Triatoma dimidiata] Triatoma dimidiata cytochrome b (cytb) gene

GCTATGGTAATAATCCACCTCCTATTTTTACATCAAACAGGATCTAGAAATCCGTTAGGA

TTAAACAGAAACTTTGACAAGATCCCATTCCATCCATATTTTTCAATTAAAGACCTAATA

GGAGTATCCTTAACCTTAATATTCTTTATCCTCCTAAGTCTATGGGAAGCCCCAATTTTA

ATAGACCCAGAAAACTTTATTCCCGCAAACCCACTAGTTACACCAGTACATATTCAACCA

GAATGATACTTCCTATT

>st27_TDEC_CytB_7_[organism=Triatoma dimidiata] Triatoma dimidiata cytochrome b (cytb) gene

GCTATGGTAATAATCCACCTCCTATTTTTACATCAAACAGGATCTAGAAATCCGTTAGGA

TTAAACAGAAACTTTGACAAGATCCCATTCCATCCATATTTTTCAATTAAAGACCTAATA

GGAGTATCCTTAACCTTAATATTCTTTATCCTCCTAAGTCTATGGGAAGCCCCAATTTTA

ATAGACCCAGAAAACTTTATTCCCGCAAACCCACTAGTTACACCAGTACATATTCAACCA

GAATGATACTTCCTATT

>st28_TDEC_CytB_8_[organism=Triatoma dimidiata] Triatoma dimidiata cytochrome b (cytb) gene

GCTATGGTAATAATCCACCTCCTATTTTTACATCAAACAGGATCTAGAAATCCGTTAGGA

TTAAACAGAAACTTTGACAAGATCCCATTCCATCCATATTTTTCAATTAAAGACCTAATA

GGAGTATCCTTAACCTTAATATTCTTTATCCTCCTAAGTCTATGGGAAGCCCCAATTTTA

ATAGACCCAGAAAACTTTATTCCCGCAAACCCACTAGTTACACCAGTACATATTCAACCA

GAATGATACTTCCTATT

>st21_TDEC_CytB_11_[organism=Triatoma dimidiata] Triatoma dimidiata cytochrome b (cytb) gene

GCTATGGTAATAATCCACCTCCTATTTTTACATCAAACAGGATCTAGAAATCCGTTAGGA

TTAAACAGAAACTTTGACAAGATCCCATTCCATCCATATTTTTCAATTAAAGACCTAATA

GGAGTATCCTTAACCTTAATATTCTTTATCCTCCTAAGTCTATGGGAAGCCCCAATTTTA

ATAGACCCAGAAAACTTTATTCCCGCAAACCCACTAGTTACACCAGTACATATTCAACCA

GAATGATACTTCCTATT

>st22_TDEC_CytB_12_[organism=Triatoma dimidiata] Triatoma dimidiata cytochrome b (cytb) gene

GCTATGGTAATAATCCACCTCCTATTTTTACATCAAACAGGATCTAGAAATCCGTTAGGA

TTAAACAGAAACTTTGACAAGATCCCATTCCATCCATATTTTTCAATTAAAGACCTAATA

GGAGTATCCTTAACCTTAATATTCTTTATCCTCCTAAGTCTATGGGAAGCCCCAATTTTA

ATAGACCCAGAAAACTTTATTCCCGCAAACCCACTAGTTACACCAGTACATATTCAACCA

GAATGATACTTCCTATT

>st23_TDEC_CytB_13_[organism=Triatoma dimidiata] Triatoma dimidiata cytochrome b (cytb) gene

GCTATGGTAATAATCCACCTCCTATTTTTACATCAAACAGGATCTAGAAATCCGTTAGGA

TTAAACAGAAACTTTGACAAGATCCCATTCCATCCATATTTTTCAATTAAAGACCTAATA

GGAGTATCCTTAACCTTAATATTCTTTATCCTCCTAAGTCTATGGGAAGCCCCAATTTTA

ATAGACCCAGAAAACTTTATTCCCGCAAACCCACTAGTTACACCAGTACATATTCAACCA

GAATGATACTTCCTATT

>st24_TDEC_CytB_14_[organism=Triatoma dimidiata] Triatoma dimidiata cytochrome b (cytb) gene

GCTATGGTAATAATCCACCTCCTATTTTTACATCAAACAGGATCTAGAAATCCGTTAGGA

TTAAACAGAAACTTTGACAAGATCCCATTCCATCCATATTTTTCAATTAAAGACCTAATA

GGAGTATCCTTAACCTTAATATTCTTTATCCTCCTAAGTCTATGGGAAGCCCCAATTTTA

ATAGACCCAGAAAACTTTATTCCCGCAAACCCACTAGTTACACCAGTACATATTCAACCA

GAATGATACTTCCTATT

>st25_TDEC_CytB_15_[organism=Triatoma dimidiata] Triatoma dimidiata cytochrome b (cytb) gene

GCTATGGTAATAATCCACCTCCTATTTTTACATCAAACAGGATCTAGAAATCCGTTAGGA

TTAAACAGAAACTTTGACAAGATCCCATTCCATCCATATTTTTCAATTAAAGACCTAATA

GGAGTATCCTTAACCTTAATATTCTTTATCCTCCTAAGTCTATGGGAAGCCCCAATTTTA

ATAGACCCAGAAAACTTTATTCCCGCAAACCCACTAGTTACACCAGTACATATTCAACCA

GAATGATACTTCCTATT

>st26_TDEC_CytB_16_[organism=Triatoma dimidiata] Triatoma dimidiata cytochrome b (cytb) gene

GCTATGGTAATAATCCACCTCCTATTTTTACATCAAACAGGATCTAGAAATCCGTTAGGA

TTAAACAGAAACTTTGACAAGATCCCATTCCATCCATATTTTTCAATTAAAGACCTAATA

GGAGTATCCTTAACCTTAATATTCTTTATCCTCCTAAGTCTATGGGAAGCCCCAATTTTA

ATAGACCCAGAAAACTTTATTCCCGCAAACCCACTAGTTACACCAGTACATATTCAACCA

GAATGATACTTCCTATT

>st27_TDEC_CytB_17_[organism=Triatoma dimidiata] Triatoma dimidiata cytochrome b (cytb) gene

GCTATGGTAATAATCCACCTCCTATTTTTACATCAAACAGGATCTAGAAATCCGTTAGGA

TTAAACAGAAACTTTGACAAGATCCCATTCCATCCATATTTTTCAATTAAAGACCTAATA

GGAGTATCCTTAACCTTAATATTCTTTATCCTCCTAAGTCTATGGGAAGCCCCAATTTTA

ATAGACCCAGAAAACTTTATTCCCGCAAACCCACTAGTTACACCAGTACATATTCAACCA

GAATGATACTTCCTATT

>st28_TDEC_CytB_18_[organism=Triatoma dimidiata] Triatoma dimidiata cytochrome b (cytb) gene

GCTATGGTAATAATCCACCTCCTATTTTTACATCAAACAGGATCTAGAAATCCGTTAGGA

TTAAACAGAAACTTTGACAAGATCCCATTCCATCCATATTTTTCAATTAAAGACCTAATA

GGAGTATCCTTAACCTTAATATTCTTTATCCTCCTAAGTCTATGGGAAGCCCCAATTTTA

ATAGACCCAGAAAACTTTATTCCCGCAAACCCACTAGTTACACCAGTACATATTCAACCA

GAATGATACTTCCTATT

>st29_TDEC_CytB_19_[organism=Triatoma dimidiata] Triatoma dimidiata cytochrome b (cytb) gene

GCTATGGTAATAATCCACCTCCTATTTTTACATCAAACAGGATCTAGAAATCCGTTAGGA

TTAAACAGAAACTTTGACAAGATCCCATTCCATCCATATTTTTCAATTAAAGACCTAATA

GGAGTATCCTTAACCTTAATATTCTTTATCCTCCTAAGTCTATGGGAAGCCCCAATTTTA

ATAGACCCAGAAAACTTTATTCCCGCAAACCCACTAGTTACACCAGTACATATTCAACCA

GAATGATACTTCCTATT

>st30_TDEC_CytB_20_[organism=Triatoma dimidiata] Triatoma dimidiata cytochrome b (cytb) gene

GCTATGGTAATAATCCACCTCCTATTTTTACATCAAACAGGATCTAGAAATCCGTTAGGA

TTAAACAGAAACTTTGACAAGATCCCATTCCATCCATATTTTTCAATTAAAGACCTAATA

GGAGTATCCTTAACCTTAATATTCTTTATCCTCCTAAGTCTATGGGAAGCCCCAATTTTA

ATAGACCCAGAAAACTTTATTCCCGCAAACCCACTAGTTACACCAGTACATATTCAACCA

GAATGATACTTCCTATT

>st31_TDMR_CytB_1_[organism=Triatoma dimidiata] Triatoma dimidiata cytochrome b (cytb) gene

GCTATGGTAATAATCCACCTCCTATTTTTACATCAAACAGGATCTAGAAATCCGTTAGGA

TTAAACAGAAACTTTGACAAGATCCCATTCCATCCATATTTTTCAATTAAAGACCTAATA

GGAGTATCCTTAACCTTAATATTCTTTATCCTCCTAAGTCTATGAGAAGCCCCAATTTTA

ATAGACCCAGAAAACTTTATTCCCGCAAACCCACTAGTTACACCAGTACATATTCAACCA

GAATGATACTTCCTATT

>st32_TDMR_CytB_2_[organism=Triatoma dimidiata] Triatoma dimidiata cytochrome b (cytb) gene

GCTATGGTAATAATCCACCTCCTATTTTTACATCAAACAGGATCTAGAAATCCGTTAGGA

TTAAACAGAAACTTTGACAAGATCCCATTCCATCCATATTTTTCAATTAAAGACCTAATA

GGAGTATCCTTAACCTTAATATTCTTTATCCTCCTAAGTCTATGAGAAGCCCCAATTTTA

ATAGACCCAGAAAACTTTATTCCCGCAAACCCACTAGTTACACCAGTACATATTCAACCA

GAATGATACTTCCTATT

>st33_TDMR_CytB_3_[organism=Triatoma dimidiata] Triatoma dimidiata cytochrome b (cytb) gene

GCTATGGTAATAGTCCACCTCCTATTTTTACATCAAACAGGATCTAGAAATCCGTTAGGA

TTAAACAGAAACTTTGACAAGATTCCATTCCATCCATATTTTTCAATTAAAGACCTAATA

GGAGTATCCTTAACCTTAATATTCTTTACCCTCCTAAGTCTATGAGAAGCCCCAATTTTA

ATAGACCCAGAAAACTTTATTCCCGCAAACCCACTAGTTACACCAGTACATATTCAACCA

GAATGATACTTCCTATT

>st34_TDMR_CytB_4_[organism=Triatoma dimidiata] Triatoma dimidiata cytochrome b (cytb) gene

GCTATGGTAATAGTCCACCTCCTATTTTTACATCAAACAGGATCTAGAAATCCGTTAGGA

TTAAACAGAAACTTTGACAAGATTCCATTCCATCCATATTTTTCAATTAAAGACCTAATA

GGAGTATCCTTAACCTTAATATTCTTTACCCTCCTAAGTCTATGAGAAGCCCCAATTTTA

ATAGACCCAGAAAACTTTATTCCCGCAAACCCACTAGTTACACCAGTACATATTCAACCA

GAATGATACTTCCTATT

>st35_TDMR_CytB_5_[organism=Triatoma dimidiata] Triatoma dimidiata cytochrome b (cytb) gene

GCTATGGTAATAGTCCACCTCCTATTTTTACATCAAACAGGATCTAGAAATCCGTTAGGA

TTAAACAGAAACTTTGACAAGATTCCATTCCATCCATATTTTTCAATTAAAGACCTAATA

GGAGTATCCTTAACCTTAATATTCTTTACCCTCCTAAGTCTATGAGAAGCCCCAATTTTA

ATAGACCCAGAAAACTTTATTCCCGCAAACCCACTAGTTACACCAGTACATATTCAACCA

GAATGATACTTCCTATT

>st36_TDMR_CytB_6_[organism=Triatoma dimidiata] Triatoma dimidiata cytochrome b (cytb) gene

GCTATGGTAATAGTCCACCTCCTATTTTTACATCAAACAGGATCTAGAAATCCGTTAGGA

TTAAACAGAAACTTTGACAAGATTCCATTCCATCCATATTTTTCAATTAAAGACCTAATA

GGAGTATCCTTAACCTTAATATTCTTTACCCTCCTAAGTCTATGAGAAGCCCCAATTTTA

ATAGACCCAGAAAACTTTATTCCCGCAAACCCACTAGTTACACCAGTACATATTCAACCA

GAATGATACTTCCTATT

>st37_TDMR_CytB_7_[organism=Triatoma dimidiata] Triatoma dimidiata cytochrome b (cytb) gene

GCTATGGTAATAATCCACCTCCTATTTTTACATCAAACAGGATCTAGAAATCCGTTAGGA

TTAAACAGAAACTTTGACAAGATCCCATTCCATCCATATTTTTCAATTAAAGACCTAATA

GGAGTATCCTTAACCTTAATATTCTTTATCCTCCTAAGTCTATGAGAAGCCCCAATTTTA

ATAGACCCAGAAAACTTTATTCCCGCAAACCCACTAGTTACACCAGTACATATTCAACCA

GAATGATACTTCCTATT

>st38_TDMR_CytB_8_[organism=Triatoma dimidiata] Triatoma dimidiata cytochrome b (cytb) gene

GCTATGGTAATAGTCCACCTCCTATTTTTACATCAAACAGGATCTAGAAATCCGTTAGGA

TTAAACAGAAACTTTGACAAGATTCCATTCCATCCATATTTTTCAATTAAAGACCTAATA

GGAGTATCCTTAACCTTAATATTCTTTACCCTCCTAAGTCTATGAGAAGCCCCAATTTTA

ATAGACCCAGAAAACTTTATTCCCGCAAACCCACTAGTTACACCAGTACATATTCAACCA

GAATGATACTTCCTATT

>st39_TDMR_CytB_9_[organism=Triatoma dimidiata] Triatoma dimidiata cytochrome b (cytb) gene

GCTATGGTAATAATCCACCTCCTATTTTTACATCAAACAGGATCTAGAAATCCGTTAGGA

TTAAACAGAAACTTTGACAAGATCCCATTCCATCCATATTTTTCAATTAAAGACCTAATA

GGAGTATCCTTAACCTTAATATTCTTTATCCTCCTAAGTCTATGAGAAGCCCCAATTTTA

ATAGACCCAGAAAACTTTATTCCCGCAAACCCACTAGTTACACCAGTACATATTCAACCA

GAATGATACTTCCTATT

>st40_TDMR_CytB_10_[organism=Triatoma dimidiata] Triatoma dimidiata cytochrome b (cytb) gene

GCTATGGTAATAGTCCACCTCCTATTTTTACATCAAACAGGATCTAGAAATCCGTTAGGA

TTAAACAGAAACTTTGACAAGATTCCATTCCATCCATATTTTTCAATTAAAGACCTAATA

GGAGTATCCTTAACCTTAATATTCTTTACCCTCCTAAGTCTATGAGAAGCCCCAATTTTA

ATAGACCCAGAAAACTTTATTCCCGCAAACCCACTAGTTACACCAGTACATATTCAACCA

GAATGATACTTCCTATT

>st31_TDMR_CytB_11_[organism=Triatoma dimidiata] Triatoma dimidiata cytochrome b (cytb) gene

GCTATGGTAATAATCCACCTCCTATTTTTACATCAAACAGGATCTAGAAATCCGTTAGGA

TTAAACAGAAACTTTGACAAGATCCCATTCCATCCATATTTTTCAATTAAAGACCTAATA

GGAGTATCCTTAACCTTAATATTCTTTATCCTCCTAAGTCTATGAGAAGCCCCAATTTTA

ATAGACCCAGAAAACTTTATTCCCGCAAACCCACTAGTTACACCAGTACATATTCAACCA

GAATGATACTTCCTATT

>st32_TDMR_CytB_12_[organism=Triatoma dimidiata] Triatoma dimidiata cytochrome b (cytb) gene

GCTATGGTAATAATCCACCTCCTATTTTTACATCAAACAGGATCTAGAAATCCGTTAGGA

TTAAACAGAAACTTTGACAAGATCCCATTCCATCCATATTTTTCAATTAAAGACCTAATA

GGAGTATCCTTAACCTTAATATTCTTTATCCTCCTAAGTCTATGAGAAGCCCCAATTTTA

ATAGACCCAGAAAACTTTATTCCCGCAAACCCACTAGTTACACCAGTACATATTCAACCA

GAATGATACTTCCTATT

>st33_TDMR_CytB_13_[organism=Triatoma dimidiata] Triatoma dimidiata cytochrome b (cytb) gene

GCTATGGTAATAGTCCACCTCCTATTTTTACATCAAACAGGATCTAGAAATCCGTTAGGA

TTAAACAGAAACTTTGACAAGATTCCATTCCATCCATATTTTTCAATTAAAGACCTAATA

GGAGTATCCTTAACCTTAATATTCTTTACCCTCCTAAGTCTATGAGAAGCCCCAATTTTA

ATAGACCCAGAAAACTTTATTCCCGCAAACCCACTAGTTACACCAGTACATATTCAACCA

GAATGATACTTCCTATT

>st34_TDMR_CytB_14_[organism=Triatoma dimidiata] Triatoma dimidiata cytochrome b (cytb) gene

GCTATGGTAATAATCCACCTCCTATTTTTACATCAAACAGGATCTAGAAATCCGTTAGGA

TTAAACAGAAACTTTGACAAGATCCCATTCCATCCATATTTTTCAATTAAAGACCTAATA

GGAGTATCCTTAACCTTAATATTCTTTATCCTCCTAAGTCTATGAGAAGCCCCAATTTTA

ATAGACCCAGAAAACTTTATTCCCGCAAACCCACTAGTTACACCAGTACATATTCAACCA

GAATGATACTTCCTATT

>st35_TDMR_CytB_15_[organism=Triatoma dimidiata] Triatoma dimidiata cytochrome b (cytb) gene

GCTATGGTAATAATCCACCTCCTATTTTTACATCAAACAGGATCTAGAAATCCGTTAGGA

TTAAACAGAAACTTTGACAAGATCCCATTCCATCCATATTTTTCAATTAAAGACCTAATA

GGAGTATCCTTAACCTTAATATTCTTTATCCTCCTAAGTCTATGAGAAGCCCCAATTTTA

ATAGACCCAGAAAACTTTATTCCCGCAAACCCACTAGTTACACCAGTACATATTCAACCA

GAATGATACTTCCTATT

>st36_TDMR_CytB_16_[organism=Triatoma dimidiata] Triatoma dimidiata cytochrome b (cytb) gene

GCTATGGTAATAGTCCACCTCCTATTTTTACATCAAACAGGATCTAGAAATCCGTTAGGA

TTAAACAGAAACTTTGACAAGATTCCATTCCATCCATATTTTTCAATTAAAGACCTAATA

GGAGTATCCTTAACCTTAATATTCTTTACCCTCCTAAGTCTATGAGAAGCCCCAATTTTA

ATAGACCCAGAAAACTTTATTCCCGCAAACCCACTAGTTACACCAGTACATATTCAACCA

GAATGATACTTCCTATT

>st37_TDMR_CytB_17_[organism=Triatoma dimidiata] Triatoma dimidiata cytochrome b (cytb) gene

GCTATGGTAATAGTCCACCTCCTATTTTTACATCAAACAGGATCTAGAAATCCGTTAGGA

TTAAACAGAAACTTTGACAAGATTCCATTCCATCCATATTTTTCAATTAAAGACCTAATA

GGAGTATCCTTAACCTTAATATTCTTTACCCTCCTAAGTCTATGAGAAGCCCCAATTTTA

ATAGACCCAGAAAACTTTATTCCCGCAAACCCACTAGTTACACCAGTACATATTCAACCA

GAATGATACTTCCTATT

>st38_TDMR_CytB_18_[organism=Triatoma dimidiata] Triatoma dimidiata cytochrome b (cytb) gene

GCTATGGTAATAATCCACCTCCTATTTTTACATCAAACAGGATCTAGAAATCCGTTAGGA

TTAAACAGAAACTTTGACAAGATCCCATTCCATCCATATTTTTCAATTAAAGACCTAATA

GGAGTATCCTTAACCTTAATATTCTTTATCCTCCTAAGTCTATGAGAAGCCCCAATTTTA

ATAGACCCAGAAAACTTTATTCCCGCAAACCCACTAGTTACACCAGTACATATTCAACCA

GAATGATACTTCCTATT

>st39_TDMR_CytB_19_[organism=Triatoma dimidiata] Triatoma dimidiata cytochrome b (cytb) gene

GCTATGGTAATAGTCCACCTCCTATTTTTACATCAAACAGGATCTAGAAATCCGTTAGGA

TTAAACAGAAACTTTGACAAGATTCCATTCCATCCATATTTTTCAATTAAAGACCTAATA

GGAGTATCCTTAACCTTAATATTCTTTACCCTCCTAAGTCTATGAGAAGCCCCAATTTTA

ATAGACCCAGAAAACTTTATTCCCGCAAACCCACTAGTTACACCAGTACATATTCAACCA

GAATGATACTTCCTATT

>st40_TDMR_CytB_20_[organism=Triatoma dimidiata] Triatoma dimidiata cytochrome b (cytb) gene

GCTATGGTAATAGTCCACCTCCTATTTTTACATCAAACAGGATCTAGAAATCCGTTAGGA

TTAAACAGAAACTTTGACAAGATTCCATTCCATCCATATTTTTCAATTAAAGACCTAATA

GGAGTATCCTTAACCTTAATATTCTTTACCCTCCTAAGTCTATGAGAAGCCCCAATTTTA

ATAGACCCAGAAAACTTTATTCCCGCAAACCCACTAGTTACACCAGTACATATTCAACCA

GAATGATACTTCCTATT
