# Supplementary material for: First Report of the L925I kdr Mutation Associated with Pyrethroid Resistance in Genetically Distinct Triatoma dimidiata, Vector of Chagas Disease in Mexico
Source: Trop Med Infect Dis. 2025 Jun 27;10(7):182. doi: 10.3390/tropicalmed10070182 (PMC12299954; doi:10.3390/tropicalmed10070182)
Supplement: Supplementary file 1 [file tropicalmed-10-00182-s001.zip › File S3. Sequences_VGSC_fragment.pdf]

>st81_TDCH_CNA_1_[ORGANISM=*Triatoma dimidiata*] *Triatoma dimidiata* SODIUM VOLTAGE-GATED CHANNEL (VGSC)

CTTGGCAATTTGACATTTGTCTTATGTATTATCATCTTTATATTTGCTGTAATGGGCATGC
AATTGTTTGGCAAGAATTATACAGGTCAGTTTAATATTTTTCTTAACATTAAATACGTAAAA
ACAATTTTAATCTAATTTAATAAATATAAAATTACTTTTTTCTTTTTTTTTCTTATCTGTTCTA
TCTTTTATTTCTCAAACAAATCTCTGTTTCTACACTTTCTTTAAACAGATAATGTGGAAAA
TTTTCCTGGTGGAGAACTACCTAGATGGAATTTTACAGATTTCATGCATTCCTTTATGAT
TGTTTTCCGTGTATTATGTGGAGAATGGATAGAATCAATGTGGGACTGCATGCACGTA

>st82_TDCH_CNA_2_[ORGANISM=*Triatoma dimidiata*] *Triatoma dimidiata* SODIUM VOLTAGE-GATED CHANNEL (VGSC)

CTTGGCAATTTGACATTTGTCTTATGTATTATCATCTTTATATTTGCTGTAATGGGCATGC
AATTGTTTGGCAAGAATTATACAGGTCAGTTTAATATTTTTCTTAACATTAAATACGTAAAA
ACAATTTTAATCTAATTTAATAAATATAAAATTACTTTTTTCTTTTTTTTTCTCATCTACTCTA
TCTTTTATTTCTCAAACAAATCTCTGTTTCTACACTTTCTTTAAACAAATAATGGGAAAAA
TTTTCCTGGGGGAAAACTACCTAAATGGAATTTTACAAATTTCATGCTTTCCTTTATGATT
GTTTCCCGTGTATTATGGGGAAAATGGATAAAATCAATGGGGGACTGCATGCACGAA

>st83_TDCH_CNA_3_[ORGANISM=*Triatoma dimidiata*] *Triatoma dimidiata* SODIUM VOLTAGE-GATED CHANNEL (VGSC)

CTTGGCAATTTGACATTTGTCTTATGTATTATCATCTTTATATTTGCTGTAATGGGCATGC
AATTGTTTGGCAAGAATTATACAGGTCAGTTTAATATTTTTCTTAACATTAAATACGTAAAA
ACAATTTTAATCTAATTTAATAAATATAAAATTACTTTTTTCTTTTTTTTTCTTATCTGTTCTA
TCTTTTATTTCTCAAACAAATCTCTGTTTCTACACTTTCTTTAAACAGATAATGTGGAAAA
TTTTCCTGGTGGAGAACTACCTAGATGGAATTTTACAGATTTCATGCATTCCTTTATGAT
TGTTTTCCGTGTATTATGTGGAGAATGGATAGAATCAATGTGGGACTGCATGCACGTA

>st84_TDCH_CNA_4_[ORGANISM=*Triatoma dimidiata*] *Triatoma dimidiata* SODIUM VOLTAGE-GATED CHANNEL (VGSC)

CTTGGCAATTTGACATTTGTCTTATGTATTATCATCTTTATATTTGCTGTAATGGGCATGC
AATTGTTTGGCAAGAATTATACAGGTCAGTTTAATATTTTTCTTAACATTAAATACGTAAAA
ACAATTTTAATCTAATTTAATAAATATAAAATTACTTTTTTCTTTTTTTTTCTCATCTACTCTA
TCTTTTATTTCTCAAACAAATCTCTGTTTCTACACTTTCTTTAAACAAATAATGGGAAAAA
TTTTCCTGGGGGAAAACTACCTAAATGGAATTTTACAAATTTCATGCTTTCCTTTATGATT
GTTTCCCGTGTATTATGGGGAAAATGGATAAAATCAATGGGGGACTGCATGCACGAA

>st85_TDCH_CNA_5_[ORGANISM=*Triatoma dimidiata*] *Triatoma dimidiata* SODIUM VOLTAGE-GATED CHANNEL (VGSC)

CTTGGCAATTTGACATTTGTCTTATGTATTATCATCTTTATATTTGCTGTAATGGGCATGC
AATTGTTTGGCAAGAATTATACAGGTCAGTTTAATATTTTTCTTAACATTAAATACGTAAAA
ACAATTTTAATCTAATTTAATAAATATAAAATTACTTTTTTCTTTTTTTTTCTCATCTACTCTA
TCTTTTTTTTCTCAAACAAATCTCTGTTTCTACACTTTCTTTAAACAGATAATGGGAAAAA
TTTTCCTGGGGAAGAACTACCTAAATGGAATTTTACAGATTTCATGCATTCCTTTATGAT
TGTTTCCCGTGTATTATGGGAAGAATGGATAGAATCAAGGGGGGACTGCATGCACGTA

>st86_TDCH_CNA_6_[ORGANISM=*Triatoma dimidiata*] *Triatoma dimidiata* SODIUM VOLTAGE-GATED CHANNEL (VGSC)

CTTGGCAATTTGACATTTGTCTTATGTATTATCATCTTTATATTTGCTGTAATGGGCATGC
AATTGTTTGGCAAGAATTATACAGGTCAGTTTAATATTTTTCTTAACATTAAATACGTAAAA
ACAATTTTAATCTAATTTAATAAATATAAAATTACTTTTTTCTTTTTTTTTCTTATCTGTTCTA
TCTTTTATTTCTCAAACAAATCTCTGTTTCTACACTTTCTTTAAACAGATAATGTGGAAAA
TTTTCCTGGTGGAGAACTACCTAGATGGAATTTTACAGATTTCATGCATTCCTTTATGAT
TGTTTTCCGTGTATTATGTGGAGAATGGATAGAATCAATGTGGGACTGCATGCACGTA

>st87_TDCH_CNA_7_[ORGANISM=*Triatoma dimidiata*] *Triatoma dimidiata* SODIUM VOLTAGE-GATED CHANNEL (VGSC)

CTTGGCAATTTGACATTTGTCTTATGTATTATCATCTTTATATTTGCTGTAATGGGCATGC
AATTGTTTGGCAAGAATTATACAGGTCAGTTTAATATTTTTCTTAACATTAAATACGTAAAA
ACAATTTTAATCTAATTTAATAAATATAAAATTACTTTTTTCTTTTTTTTTCTTATCTACTCTA
TCTTTTATTTCTCAAACAAATCTCTGTTTCTACACTTTCTTTAAACAGATAATGGGGAAAA
TTTTCCTGGGGGAAAACTACCTAGATGGAATTTTACAGATTTCATGCATTCCTTTATGAT
TGTTTTCCGTGTATTATGGGGAGAATGGATAGAATCAATGGGGGACTGCATGCACGAA

>st88_TDCH_CNA_8_[ORGANISM=*Triatoma dimidiata*] *Triatoma dimidiata* SODIUM VOLTAGE-GATED CHANNEL (VGSC)

CTTGGCAATTTGACATTTGTCTTATGTATTATCATCTTTATATTTGCTGTAATGGGCATGC
AATTGTTTGGCAAGAATTATACAGGTCAGTTTAATATTTGTCTTAACATTAAATACGTAAA
AACAATTTTAATCTAATTTAATAAATATAAAATTACTTTTTTCTTTTTTTTTCTAATCTACTCT
ATCTTTTATTTCTCAAACAAATCTCTGTTTCTACACTTTCTTTAAACAAATAATGGGAAAA
ATTTTCCGGGGGAAGAACTACCTAGATGGAATTTTACAGATTTCATGCTTTCCTTTATGA
TTGTTTTCCGTGTATTATGTGGAAAATGGATAGAATCAATGGGGGACTGCATGCACAAA

>st89_TDCH_CNA_9_[ORGANISM=*Triatoma dimidiata*] *Triatoma dimidiata* SODIUM VOLTAGE-GATED CHANNEL (VGSC)

CTTGGCAATTTGACATTTGTCTTATGTATTATCATCTTTATATTTGCTGTAATGGGCATGC
AATTGTTTGGCAAGAATTATACAGGTCAGTTTAATATTTTTCTTAACATTAAATACGTAAAA
ACAATTTTAATCTAATTTAATAAATATAAAATTACTTTTTTCTTTTTTTTTCTTATCTACTCTA
TCTTTTATTTCTCAAACAAATCTCTGTTTCTACACTTTCTTTAAACAGATAATGTGGAAAA
TTTTCCTGGGGGAGAACTACCTAGATGGAATTTTACAGATTTCATGCATTCCTTTATGAT
TGTTTTCCGTGTATTATGTGGAGAATGGATAGAATCAATGTGGGACTGCATGCACGTA

>st81_TDCH_CNA_11_[ORGANISM=*Triatoma dimidiata*] *Triatoma dimidiata* SODIUM VOLTAGE-GATED CHANNEL (VGSC)

ATTGGCAATTTGACATTTGGCTTATGTATTATCATCTTTATATTTGCTGTAATGGGCATGC
AATTGTTTGGCAAGAATTATACAGGTCAGTTTAATATTTTTCTTAACATTAAATACGTAAAA
ACAATTTTAATCTAATTTAATAAATATAAAATTACTTTTTTCTTTTTTTTTCTTATCTGTTCTA
CCTTTTATTTCTCAAACAAATCTCTGTTTCTACACTTTCTTTAAACAGATAATGGGGAAAA
TTTTCCTGGGGGAGAACTACCTAGATGGAATTTTACAGATTTCATGCATTCCTTTATGAT
TGTTTTCCGTGTATTATGTGGAGAATGGATAGAATCAATGTGGGACTGCATGCACGTA

>st90_TDCH_CNA_10_[ORGANISM=*Triatoma dimidiata*] *Triatoma dimidiata* SODIUM VOLTAGE-GATED CHANNEL (VGSC)

CTTGGCAATTTGACATTTGTCTTATGTATTATCATCTTTATATTTGCTGTAATGGGCATGC
AATTGTTTGGCAAGAATTATACAGGTCAGTTTAATATTTTTCTTAACATTAAATACGTAAAA
ACAATTTTAATCTAATTTAATAAATATAAAATTACTTTTTTCTTTTTTTTTCTCATCTGCTCT
ATCTTTTATTTCTCAAACAAATCTCTGTTTCTACACTTTCTTTAAACAGATAATGGGAAAA
ATTTTCCGGGGGAAGAACTACCTAGATGGAATTTTACAGATTTCATGCTTTCCTTTATGA
TTGTTTTCCGTGTATTATGTGGAAAATGGATAGAATCAATGGGGGACTGCATGCACAAA

>st82_TDCH_CNA_12_[ORGANISM=*Triatoma dimidiata*] *Triatoma dimidiata* SODIUM VOLTAGE-GATED CHANNEL (VGSC)

CTTGGCAATTTGACATTTGTCTTATGTATTATCATCTTTATATTTGCTGTAATGGGCATGC
AATTGTTTGGCAAGAATTATACAGGTCAGTTTAATATTTTTCTTAACATTAAATACGTAAAA
ACAATTTTAATCTAATTTAATAAATATAAAATTACTTTTTTCTTTTTTTTTCTAATCGGTCCA
ACCTTTTATTTCCCAAAAAAATCTCTGTTTCTACCCTTTCTTTAAACAAAAAAGGGGGAA
AAATTTCCCGGGGGAAAACTACCCAAAAGGAATTTTACCAAATTCCTGGCTTCCTTTAA
GAATGGTTTCCCGGGATTAAGGGGAAAAAGGAAAAAAACCATGGGGGAATGGCTGGC
CGTA

>st83_TDCH_CNA_13_[ORGANISM=*Triatoma dimidiata*] *Triatoma dimidiata* SODIUM VOLTAGE-GATED CHANNEL (VGSC)

CTTGGCAATTTGACATTTGTCTTATGTATTATCATCTTTATATTTGCTGTAATGGGCATGC
AATTGTTTGGCAAGAATTATACAGGTCAGTTTAATATTTTTCTTAACATTAATTACGTAAAA
ACAATTTTAATCTAATTTAATAAATATAAAATTACTTTTTTCTTTTTTTTTCTTATCTGTTCCA
TCTTTTATTTCTCAAACAAATCTCTGTTTCTACACTTTCTTTAAACAGATAATGTGGAAAA
TTTTCCTGGTGGAGAACTACCTAGATGGAATTTTACAGATTTCATGCATTCCTTTATGAT
TGTTTTCCGTGTATTATGTGGAGAATGGATAGAATCAATGTGGGACTGCATGCACGTA

>st84_TDCH_CNA_14_[ORGANISM=*Triatoma dimidiata*] *Triatoma dimidiata* SODIUM VOLTAGE-GATED CHANNEL (VGSC)

CTTGGCAATTTGACATTTGTCTTATGTATTATCATCTTTATATTTGCTGTAATGGGCATGC
AATTGTTTGGCAAGAATTATACAGGTCAGTTTAATATTTTTCTTAACATTAAATACGTAAAA
ACAATTTTAATCTAATTTAATAAATATAAAATTACTTTTTTCTTTTTTTTTCTTATCTGTCCC
CCCTTTTATTTCTCAAAAAAATCTCTGTTTCTACACTTTCTTTAAAAAAAAAAAGGGGAA
AAATTTCCTGGGGGGGAACTACCCAAATGGAATTTTACAGATTTCTTGCGTTCCTTTTT
GATTGTTTTCCCCGTATTATGTGGAGAAAGGATAGAAACAATGGGGGACTGCGTGCGC
GTA

>st85_TDCH_CNA_15_[ORGANISM=*Triatoma dimidiata*] *Triatoma dimidiata* SODIUM VOLTAGE-GATED CHANNEL (VGSC)

CTTGGCAATTTGACATTTGTCTTATGTATTATCATCTTTATATTTGCTGTAATGGGCATGC
AATTGTTTGGCAAGAATTATACAGGTCAGTTTAATATTTTTCTTAACATTAAATACGTAAAA
ACAATTTTAATCTAATTTAATAAATATAAAATTACTTTTTTCTTTTTTTTTCTTATCTGTCCC
CCCTTTTATTTCTCAAAAAAATCTCTGTTTCTACACTTTCTTTAAAAAAAAAAAGGGGAA
AAATTTCCTGGGGGGGAACTACCCAAATGGAATTTTACAGATTTCTTGCGTTCCTTTTT

GATTGTTTTCCCCGTATTATGTGGAGAAAGGATAGAAACAATGGGGGACTGCGTGCGC
GTA

>st86_TDCH_CNA_16_[ORGANISM=*Triatoma dimidiata*] *Triatoma dimidiata* SODIUM VOLTAGE-GATED CHANNEL (VGSC)

CTTGGCAATTTGACATTTGTCTTATGTATTATCATCTTTATATTTGCTGTAATGGGCATGC
AATTGTTTGGCAAGAATTATACAGGTCCCTTTAATATTTTTCTTAACATTAATTACATAAAA
ACAATTTTAATCTAATTTAATACCTATAAAATTACTTTTTTCTTTTTTTTTCTCCTCTGTTCC
ATCTTTTATTTCTCAAACAAATCTCTGTTTCTACACTTTCTTTAAACAGATAATGTGGAAA
ATTTTCCTGGTGGAGAACTACCTAGATGGAATTTTACAGATTTCATGCATTCCTTTATGA
TTGTTTTCCGTGTATTATGTGGAGAATGGATAGAATCAATGTGGGACTGCATGCACGTA

>st87_TDCH_CNA_17_[ORGANISM=*Triatoma dimidiata*] *Triatoma dimidiata* SODIUM VOLTAGE-GATED CHANNEL (VGSC)

CTTGGCAATTTGACATTTGTCTTATGTATTATCATCTTTATATTTGCTGTAATGGGCATGC
AATTGTTTGGCAAGAATTATACAGGTCAGTTTAATATTTTTCTTAACATTAAATACGTAAAA
ACAATTTTAATCTAATTTAATAAATATAAAATTACTTTTTTCTTTTTTTTTCTTATCTGTCCC
CCCTTTTATTTCTCAAAAAAAATCTCTGTTTCTACACTTTCTTTAAAAAAAAAAAGGGGAA
AAATTTCCTGGGGGGGAACTACCCAAATGGAATTTTACAGATTTCTTGCGTTCCTTTTT
GATTGTTTTCCCCGTATTATGTGGAGAAAGGATAGAAACAATGGGGGACTGCGTGCGC
GTA

>st88_TDCH_CNA_18_[ORGANISM=*Triatoma dimidiata*] *Triatoma dimidiata* SODIUM VOLTAGE-GATED CHANNEL (VGSC)

CTTGGCAATTTGACATTTGTCTTATGTATTATCATCTTTATATTTGCTGTAATGGGCATGC
AATTGTTTGGCAAGAATTATACAGGTCAGTTTAATATTTTTCTTAACATTAAATACGTAAAA
ACAATTTTAATCTAATTTAATAAATATAAAATTACTTTTTTCTTTTTTTTTCTAACCGGTCCA
CCCTTTTATTTCCCAAAACAATCCCCGGTTCCACCCTTTCTTTAAACAAAAAAGGGGGA
AAAATTTCCCGGGGGAAAAACACCCAAAAGGAATTTTACCAAATTCCTGGCTTCCCTTA
TGAATGGTTTCCCGGGATTAAGGGGAAAAAGGAAAAAAACCATGGGGGAATGGCTGC
CCGTA

>st89_TDCH_CNA_19_[ORGANISM=*Triatoma dimidiata*] *Triatoma dimidiata* SODIUM VOLTAGE-GATED CHANNEL (VGSC)

CTTGGCAATTTGACATTTGTCTTATGTATTATCATCTTTATATTTGCTGTAATGGGCATGC
AATTGTTTGGCAAGAATTATACAGGTCAGTTTAATATTTTTCTTAACATTAAATACGTAAAA
ACAATTTTAATCTAATTTAATAAATATAAAATTACTTTTTTCTTTTTTTTTCTAATCGGTCCA
ACCTTTTATTTCCCAAAAAAATCTCTGTTTCTACCCTTTCTTTAAACAAAAAAGGGGGAA
AAATTTCCCGGGGGAAAACTACCCAAAAGGAATTTTACCAAATTCCTGGCTTCCTTTAA
GAATGGTTTCCCGGGATTAAGGGGAAAAAGGAAAAAAACCATGGGGGAATGGCTGGC
CGTA

>st90_TDCH_CNA_20_[ORGANISM=*Triatoma dimidiata*] *Triatoma dimidiata* SODIUM VOLTAGE-GATED CHANNEL (VGSC)

CTTGGCAATTTGACATTTGTCTTATGTATTATCATCTTTATATTTGCTGTAATGGGCATGC
AATTGTTTGGCAAGAATTATACAGGTCAGTTTAATATTTTTCTTAACATTAATTACGTAAAA

ACAATTTTAATCTAATTTAATAAATATAAAATTACTTTTTTCTTTTTTTTTCTTATCTGTTCCA
TCTTTTATTTCTCAAACAAATCTCTGTTTCTACACTTTCTTTAAACAGATAATGTGGAAAA
TTTTCCTGGTGGAGAACTACCTAGATGGAATTTTACAGATTTCATGCATTCCTTTATGAT
TGTTTTCCGTGTATTATGTGGAGAATGGATAGAATCAATGTGGGACTGCATGCACGTA

>st91_TDCK_CNA_1_[ORGANISM=*Triatoma dimidiata*] *Triatoma dimidiata* SODIUM VOLTAGE-GATED CHANNEL (VGSC)

CTTGGCAATTTGACATTTGTCTTATGTATTATCATCTTTATATTTGCTGTAATGGGCATGC
AATTGTTTGGCAAGAATTATACAGGTCAGTTTAATATTTTTCTTAACATTAAATACGTAAAA
ACAATTTTAATCTAATTTAATAAATATAAAATTACTTTTTTCTTTTTTTTTCTTATCTGTTCTA
TCTTTTATTTCTCAAACAAATCTCTGTTTCTACACTTTCTTTAAACAGATAATGTGGAAAA
TTTTCCTGGTGGAGAACTACCTAGATGGAATTTTACAGATTTCATGCATTCCTTTATGAT
TGTTTTCCGTGTATTATGTGGAGAATGGATAGAATCAATGTGGGACTGCATGCACGTA

>st92_TDCK_CNA_2_[ORGANISM=*Triatoma dimidiata*] *Triatoma dimidiata* SODIUM VOLTAGE-GATED CHANNEL (VGSC)

CTTGGCAATTTGACATTTGTCTTATGTATTATCATCTTTATATTTGCTGTAATGGGCATGC
AATTGTTTGGCAAGAATTATACAGGTCAGTTTAATATTTTTCTTAACATTAAATACGTAAAA
ACAATTTTAATCTAATTTAATAAATATAAAATTACTTTTTTCTTTTTTTTTCTTATCTGTTCTA
TCTTTTATTTCTCAAACAAATCTCTGTTTCTACACTTTCTTTAAACAGATAATGTGGAAAA
TTTTCCTGGTGGAGAACTACCTAGATGGAATTTTACAGATTTCATGCATTCCTTTATGAT
TGTTTTCCGTGTATTATGTGGAGAATGGATAGAATCAATGTGGGACTGCATGCACGTA

>st93_TDCK_CNA_3_[ORGANISM=*Triatoma dimidiata*] *Triatoma dimidiata* SODIUM VOLTAGE-GATED CHANNEL (VGSC)

CTTGGCAATTTGACATTTGTCTTATGTATTATCATCTTTATATTTGCTGTAATGGGCATGC
AATTGTTTGGCAAGAATTATACAGGTCAGTTTAATATTTTTCTTAACATTAAATACGTAAAA
ACAATTTTAATCTAATTTAATAAATATAAAATTACTTTTTTCTTTTTTTTTCTTATCTGTTCTA
TCTTTTATTTCTCAAACAAATCTCTGTTTCTACACTTTCTTTAAACAGATAATGTGGAAAA
TTTTCCTGGTGGAGAACTACCTAGATGGAATTTTACAGATTTCATGCATTCCTTTATGAT
TGTTTTCCGTGTATTATGTGGAGAATGGATAGAATCAATGTGGGACTGCATGCACGTA

>st94_TDCK_CNA_4_[ORGANISM=*Triatoma dimidiata*] *Triatoma dimidiata* SODIUM VOLTAGE-GATED CHANNEL (VGSC)

CTTGGCAAT

TTGACATTTGTCTTATGTATTATCATCTTTATATTTGCTGTAATGGGCATGCAATTGTTTG
GCAAGAATTATACAGGTCAGTTTAATATTTTTCTTAACATTAAATACGTAAAAACAATTTTA
ATCTAATTTAATAAATATAAAATTACTTTTTTCTTTTTTTTTCTTATCTGTTCTATCTTTTATT
TCTCAAACAAATCTCTGTTTCTACACTTTCTTTAAACAGATAATGTGGAAAATTTTCCTG
GTGGAGAACTACCTAGATGGAATTTTACAGATTTCATGCATTCCTTTATGATTGTTTTCC
GTGTATTATGTGGAGAATGGATAGAATCAATGTGGGACTGCATGCACGTA

>st95_TDCK_CNA_5_[ORGANISM=*Triatoma dimidiata*] *Triatoma dimidiata* SODIUM VOLTAGE-GATED CHANNEL (VGSC)

CTTGGCAATTTGACATTTGTCTTATGTATTATCATCTTTATATTTGCTGTAATGGGCATGC
AATTGTTTGGCAAGAATTATACAGGTCAGTTTAATATTTTTCTTAACATTAAATACGTAAAA

ACAATTTTAATCTAATTTAATAAATATAAAATTACTTTTTTCTTTTTTTTTCTTATCTGTTCTA
TCTTTTATTTCTCAAACAAATCTCTGTTTCTACACTTTCTTTAAACAGATAATGTGGAAAA
TTTTCCTGGTGGAGAACTACCTAGATGGAATTTTACAGATTTCATGCATTCCTTTATGAT
TGTTTTCCGTGTATTATGTGGAGAATGGATAGAATCAATGTGGGACTGCATGCACGTA

>st96_TDCK_CNA_6_[ORGANISM=*Triatoma dimidiata*] *Triatoma dimidiata* SODIUM VOLTAGE-GATED CHANNEL (VGSC)

CTTGGCAATTTGACATTTGTCTTATGTATTATCATCTTTATATTTGCTGTAATGGGCATGC
AATTGTTTGGCAAGAATTATACAGGTCAGTTTAATATTTTTCTTAACATTAAATACGTAAAA
ACAATTTTAATCTAATTTAATAAATATAAAATTACTTTTTTCTTTTTTTTTCTTATCTGTTCTA
TCTTTTATTTCTCAAACAAATCTCTGTTTCTACACTTTCTTTAAACAGATAATGTGGAAAA
TTTTCCTGGTGGAGAACTACCTAGATGGAATTTTACAGATTTCATGCATTCCTTTATGAT
TGTTTTCCGTGTATTATGTGGAGAATGGATAGAATCAATGTGGGACTGCATGCACGTA

>st97_TDCK_CNA_7_[ORGANISM=*Triatoma dimidiata*] *Triatoma dimidiata* SODIUM VOLTAGE-GATED CHANNEL (VGSC)

CTTGGCAATTTGACATTTGTCTTATGTATTATCATCTTTATATTTGCTGTAATGGGCATGC
AATTGTTTGGCAAGAATTATACAGGTCAGTTTAATATTTTTCTTAACATTAAATACGTAAAA
ACAATTTTAATCTAATTTAATAAATATAAAATTACTTTTTTCTTTTTTTTTCTTATCTGTTCTA
TCTTTTATTTCTCAAACAAATCTCTGTTTCTACACTTTCTTTAAACAGATAATGTGGAAAA
TTTTCCTGGTGGAGAACTACCTAGATGGAATTTTACAGATTTCATGCATTCCTTTATGAT
TGTTTTCCGTGTATTATGTGGAGAATGGATAGAATCAATGTGGGACTGCATGCACGTA

>st98_TDCK_CNA_8_[ORGANISM=*Triatoma dimidiata*] *Triatoma dimidiata* SODIUM VOLTAGE-GATED CHANNEL (VGSC)

CTTGGCAATTTGACATTTGTCTTATGTATTATCATCTTTATATTTGCTGTAATGGGCATGC
AATTGTTTGGCAAGAATTATACAGGTCAGTTTAATATTTTTCTTAACATTAAATACGTAAAA
ACAATTTTAATCTAATTTAATAAATATAAAATTACTTTTTTCTTTTTTTTTCTTATCTGTTCTA
TCTTTTATTTCTCAAACAAATCTCTGTTTCTACACTTTCTTTAAACAGATAATGTGGAAAA
TTTTCCTGGTGGAGAACTACCTAGATGGAATTTTACAGATTTCATGCATTCCTTTATGAT
TGTTTTCCGTGTATTATGTGGAGAATGGATAGAATCAATGTGGGACTGCATGCACGTA

>st91_TDCK_CNA_11_[ORGANISM=*Triatoma dimidiata*] *Triatoma dimidiata* SODIUM VOLTAGE-GATED CHANNEL (VGSC)

ATTGGCAATTTGACATTTGTCTTATGTATTATCATCTTTATATTTGCTGTAATGGGCATGC
AATTGTTTGGCAAGAATTATACAGGTCAGTTTAATATTTTTCTTAACATTAAATACGTAAAA
ACAATTTTAATCTAATTTAATAAATATAAAATTACTTTTTTCTTTTTTTTTCTTATCTGCTCC
ATCTTTTATTTCTCAAACAAATCTCTGTTTCTACACTTTCTTTAAACAGATAATGTGGAAA
ATTTTCCTGGTGGAGAACTACCTAGGTGGAATTTTACAGATTTCATGCATTCCTTTATGA
TTGTTTTCCGTGTATTATGTGGAGAATGGATAGAATCAATGTGGGACTGCATGCACGTA

>st92_TDCK_CNA_12_[ORGANISM=*Triatoma dimidiata*] *Triatoma dimidiata* SODIUM VOLTAGE-GATED CHANNEL (VGSC)

CTTGGCAATTTGACATTTGTCTTATGTATTATCATCTTTATATTTGCTGTAATGGGCATGC
AATTGTTTGGCAAGAATTATACAGGTCAGTTTAACATTTTTCTTAACATTAAATACGTAAA
AACAATTTTAATCTAATTTAATAAATATAAAATTACTTTTTTCTTTTTTTTTCTTATCTGTTCT

ATCTTTTATTTCTCAAACAAATCTCTGTTTCTACACTTTCTTTAAACAGATAATGTGGAAA
ATTTTCCTGGTGGAAAACTACCTAGATGGAATTTTACAGATTTCATGCATTCCTTTATGAT
TGTTTTCCGTGTATTATGTGGAGAATGGATAGAATCAATGTGGGACTGCATGCACGTA

>st93_TDCK_CNA_13_[ORGANISM=*Triatoma dimidiata*] *Triatoma dimidiata* SODIUM VOLTAGE-GATED CHANNEL (VGSC)

CTTGGCAATTTGACATTTGTCTTATGTATTATCATCTTTATATTTGCTGTAATGGGCATGC
AATTGTTTGGCAAGAATTATACAGGTCAGTTTAATATTTTTCTTAACATTAAATACGTAAAA
ACAATTTTAATCTAATTTAATAAATATAAAATTACTTTTTTCTTTTTTTTTCTTATCTGTTCTA
TCTTTTATTTCTCAAACAAATCTCTGTTTCTACACTTTCTTTAAACAGATAATGTGGAAAA
TTTTCCTGGTGGAGAACTACCTAGATGGAATTTTACAGATTTCATGCATTCCTTTATGAT
TGTTTTCCGTGTATTATGTGGAGAATGGATAGAATCAATGTGGGACTGCATGCACGTA

>st94_TDCK_CNA_14_[ORGANISM=*Triatoma dimidiata*] *Triatoma dimidiata* SODIUM VOLTAGE-GATED CHANNEL (VGSC)

CTTGGCAATTTGACATTTGTCTTATGTATTATCATCTTTATATTTGCTGTAATGGGCATGC
AATTGTTTGGCAAGAATTATACAGGTCAGTTTAATATTTTTCTTAACATTAAATACGTAAAA
ACAATTTTAATCTAATTTAATAAATATAAAATTACTTTTTTCTTTTTTTTTCTTATCTGTTCTA
TCTTTTATTTCTCAAACAAATCTCTGTTTCTACACTTTCTTTAAACAGATAATGTGGAAAA
TTTTCCTGGTGGAGAACTACCTAGATGGAATTTTACAGATTTCATGCATTCCTTTATGAT
TGTTTTCCGTGTATTATGTGGAGAATGGATAGAATCAATGTGGGACTGCATGCACGTA

>st95_TDCK_CNA_15_[ORGANISM=*Triatoma dimidiata*] *Triatoma dimidiata* SODIUM VOLTAGE-GATED CHANNEL (VGSC)

CTTGGCAATTTGACATTTGTCTTATGTATTATCATCTTTATATTTGCTGTAATGGGCATGC
AATTGTTTGGCAAGAATTATACAGGTCAGTTTAATATTTTTCTTAACATTAAATACGTAAAA
ACAATTTTAATCTAATTTAATAAATATAAAATTACTTTTTTCTTTTTTTTTCTTATCTGTTCTA
TCTTTTATTTCTCAAACAAATCTCTGTTTCTACACTTTCTTTAAACAGATAATGTGGAAAA
TTTTCCTGGTGGAGAACTACCTAGATGGAATTTTACAGATTTCATGCATTCCTTTATGAT
TGTTTTCCGTGTATTATGTGGAGAATGGATAGAATCAATGTGGGACTGCATGCACGTA

>st96_TDCK_CNA_16_[ORGANISM=*Triatoma dimidiata*] *Triatoma dimidiata* SODIUM VOLTAGE-GATED CHANNEL (VGSC)

CTTGGCAATTTGACATTTGTCTTATGTATTATCATCTTTATATTTGCTGTAATGGGCATGC
AATTGTTTGGCAAGAATTATACAGGTCAGTTTAATATTTTTCTTAACATTAAATACGTAAAA
ACAATTTTAATCTAATTTAATAAATATAAAATTACTTTTTTCTTTTTTTTTCTTATCTGTTCTA
TCTTTTATTTCTCAAACAAATCTCTGTTTCTACACTTTCTTTAAACAGATAATGTGGAAAA
TTTTCCTGGTGGAGAACTACCTAGATGGAATTTTACAGATTTCATGCATTCCTTTATGAT
TGTTTTCCGTGTATTATGTGGAGAATGGATAGAATCAATGTGGGACTGCATGCACGTA

>st97_TDCK_CNA_17_[ORGANISM=*Triatoma dimidiata*] *Triatoma dimidiata* SODIUM VOLTAGE-GATED CHANNEL (VGSC)

CTTGGCAATTTGACATTTGTCTTATGTATTATCATCTTTATATTTGCTGTAATGGGCATGC
AATTGTTTGGCAAGAATTATACAGGTCAGTTTAATATTTTTCTTAACATTAAATACGTAAAA
ACAATTTTAATCTAATTTAATAAATATAAAATTACTTTTTTCTTTTTTTTTCTTATCTGTTCTA
TCTTTTATTTCTCAAACAAATCTCTGTTTCTACACTTTCTTTAAACAGATAATGTGGAAAA

TTTTCCTGGTGGAGAACTACCTAGATGGAATTTTACAGATTTCATGCATTCCTTTATGAT
TGTTTTCCGTGTATTATGTGGAGAATGGATAGAATCAATGTGGGACTGCATGCACGTA

>st98_TDCK_CNA_18_[ORGANISM=*Triatoma dimidiata*] *Triatoma dimidiata* SODIUM VOLTAGE-GATED CHANNEL (VGSC)

CTTGGCAATTTGACATTTGTCTTATGTATTATCATCTTTATATTTGCTGTAATGGGCATGC
AATTGTTTGGCAAGAATTATACAGGTCAGTTTAATATTTTTCTTAACATTAAATACGTAAAA
ACAATTTTAATCTAATTTAATAAATATAAAATTACTTTTTTCTTTTTTTTTCTTATCTGTTCTA
TCTTTTATTTCTCAAACAAATCTCTGTTTCTACACTTTCTTTAAACAGATAATGTGGAAAA
TTTTCCTGGTGGAGAACTACCTAGATGGAATTTTACAGATTTCATGCATTCCTTTATGAT
TGTTTTCCGTGTATTATGTGGAGAATGGATAGAATCAATGTGGGACTGCATGCACGTA

>st99_TDCK_CNA_19_[ORGANISM=*Triatoma dimidiata*] *Triatoma dimidiata* SODIUM VOLTAGE-GATED CHANNEL (VGSC)

CTTGGCAATTTGACATTTGTCTTATGTATTATCATCTTTATATTTGCTGTAATGGGCATGC
AATTGTTTGGCAAGAATTATACAGGTCAGTTTAATATTTTTCTTAACATTAAATACGTAAAA
ACAATTTTAATCTAATTTAATAAATATAAAATTACTTTTTTCTTTTTTTTTCTTATCTGTTCTA
TCTTTTATTTCTCAAACAAATCTCTGTTTCTACACTTTCTTTAAACAGATAATGTGGAAAA
TTTTCCTGGTGGAGAACTACCTAGATGGAATTTTACAGATTTCATGCATTCCTTTATGAT
TGTTTTCCGTGTATTATGTGGAGAATGGATAGAATCAATGTGGGACTGCATGCACGTA

>st100_TDCK_CNA_20_[ORGANISM=*Triatoma dimidiata*] *Triatoma dimidiata* SODIUM VOLTAGE-GATED CHANNEL (VGSC)

CTTGGCAATTTGACATTTGTCTTATGTATTATCATCTTTATATTTGCTGTAATGGGCATGC
AATTGTTTGGCAAGAATTATACAGGTCAGTTTAATATTTTTCTTAACATTAAATACGTAAAA
ACAATTTTAATCTAATTTAATAAATATAAAATTACTTTTTTCTTTTTTTTTCTTATCTGTTCTA
TCTTTTATTTCTCAAACAAATCTCTGTTTCTACACTTTCTTTAAACAGATAATGTGGAAAA
TTTTCCTGGTGGAGAACTACCTAGATGGAATTTTACAGATTTCATGCATTCCTTTATGAT
TGTTTTCCGTGTATTATGTGGAGAATGGATAGAATCAATGTGGGACTGCATGCACGTA

>st100_TDCK_CNA_10_[ORGANISM=*Triatoma dimidiata*] *Triatoma dimidiata* SODIUM VOLTAGE-GATED CHANNEL (VGSC)

CTTGGCAATTTGACATTTGTCTTATGTATTATCATCTTTATATTTGCTGTAATGGGCATGC
AATTGTTTGGCAAGAATTATACAGGTCAGTTTAATATTTTTCTTAACATTAAATACGTAAAA
ACAATTTTAATCTAATTTAATAAATATAAAATTACTTTTTTCTTTTTTTTTCTTATCTGTTCTA
TCTTTTATTTCTCAAACAAATCTCTGTTTCTACACTTTCTTTAAACAGATAATGTGGAAAA
TTTTCCTGGTGGAGAACTACCTAGATGGAATTTTACAGATTTCATGCATTCCTTTATGAT
TGTTTTCCGTGTATTATGTGGAGAATGGATAGAATCAATGTGGGACTGCATGCACGTA

>st101_TDEC_CNA_1_[ORGANISM=*Triatoma dimidiata*] *Triatoma dimidiata* SODIUM VOLTAGE-GATED CHANNEL (VGSC)

CTTGGCAATTTGACATTTGTCTTATGTATTATCATCTTTATATTTGCTGTAATGGGCATGC
AATTGTTTGGAAAGAATTATACAGGTCAGTTTAATATTTTTCTTAACATTAAATACGTAAAA
ACAATTTTAATCTAATTTAATAAATATAAAATTACTTTTTTCTTTTTTTTTCTTATCTACTCTA
TCTTTTATTTCTCAAACAAATCTCTGTTTCTACCCTTTCTTTAAACAAAAAATGGGGAAAA

TTTTCCTGGGGGAAAACTACCTAAATGGAATTTTACAAATTTCATGCATTCCTTTATGATT
GTTTTCCGTGTATTATGGGGAGAATGGATAAAATCAATGGGGGACTGCATGCACAAA

>st102_TDEC_CNA_2_[ORGANISM=*Triatoma dimidiata*] *Triatoma dimidiata* SODIUM VOLTAGE-GATED CHANNEL (VGSC)

CTTGGCAATTTGACATTTGTCTTATGTATTATCATCTTTATATTTGCTGTAATGGGCATGC
AATTGTTTGGAAAGAATTATACAGGTCAGTTTAATATTTGTCTTAACATTAAATACGTAAA
AACAATTTTAATCTAATTTAATAAATATAAAATTACTTTTTTCTTTTTTTTTCTTATCTACTCT
ATCTTTTATTTCTCAAACAAATCTCTGTTTCTACACTTTCTTTAAACAGATAATGTGGAAA
ATTTTCCTGGGGGAAAACTACCTAAATGGAATTTTACAGATTTCATGCATTCCTTTATGAT
TGTTTTCCGTGTATTATGGGGAGAATGGATAGAATCAATGGGGGACTGCATGCACAAA

>st103_TDEC_CNA_3_[ORGANISM=*Triatoma dimidiata*] *Triatoma dimidiata* SODIUM VOLTAGE-GATED CHANNEL (VGSC)

CTTGGCAATTTGACATTTGTCTTATGTATTATCATCTTTATATTTGCTGTAATGGGCATGC
AATTGTTTGGCAAGAATTATACAGGTCAGTTTAATATTTTTCTTAACATTAAATACGTAAAA
ACAATTTTAATCTAATTTAATAAATATAAAATTACTTTTTTCTTTTTTTTTCTAATCTACTCTA
TCTTTTATTTCTCAAACAAATCTCTGTTTCTACCCTTTCTTTAAACAAATAATGGGGAAAA
TTTTCCTGGGGGAAAACTACCTAAATGGAATTTTACAAATTTCATGCATTCCTTTATGATT
GTTTTCCGTGTATTATGGGGAGAATGGATAAAATCAATGGGGGACTGCATGCACAAA

>st104_TDEC_CNA_4_[ORGANISM=*Triatoma dimidiata*] *Triatoma dimidiata* SODIUM VOLTAGE-GATED CHANNEL (VGSC)

CTTGGCAATTTGACATTTGTCTTATGTATTATCATCTTTATATTTGCTGTAATGGGCATGC
AATTGTTTGGAAAGAATTATACAGGTCAGTTTAATATTTGTCTTAACATTAAATACGTAAA
AACAATTTTAATCTAATTTAATAAATATAAAATTACTTTTTTCTTTTTTTTTCTTATCTACTCT
ATCTTTTATTTCTCAAACAAATCTCTGTTTCTACCCTTTCTTTAAACAAATAATGTGGAAA
ATTTTCCTGGGGGAAAACTACCTAAATGGAATTTTACAGATTTCATGCATTCCTTTATGAT
TGTTTTCCGTGTATTATGGGGAGAATGGATAGAATCAATGGGGGACTGCATGCACAAA

>st105_TDEC_CNA_5_[ORGANISM=*Triatoma dimidiata*] *Triatoma dimidiata* SODIUM VOLTAGE-GATED CHANNEL (VGSC)

CTTGGCAATTTGACATTTGTCTTATGTATTATCATCTTTATATTTGCTGTAATGGGCATGC
AATTGTTTGGCAAGAATTATACAGGTCAGTTTAATATTTGTCTTAACATTAAATACGTAAA
AACAATTTTAATCTAATTTAATAAATATAAAATTACTTTTTTCTTTTTTTTTCTTATCTACTCT
ATCTTTTATTTCTCAAACAAATCTCTGTTTCTACACTTTCTTTAAACAGATAATGTGGAAA
ATTTTCCTGGGGGAAAACTACCTAAATGGAATTTTACAGATTTCATGCATTCCTTTATGAT
TGTTTTCCGTGTATTATGGGGAGAATGGATAGAATCAATGGGGGACTGCATGCACAAA

>st106_TDEC_CNA_6_[ORGANISM=*Triatoma dimidiata*] *Triatoma dimidiata* SODIUM VOLTAGE-GATED CHANNEL (VGSC)

CTTGGCAATTTGACATTTGTCTTATGTATTATCATCTTTATATTTGCTGTAATGGGCATGC
AATTGTTTGGCAAGAATTATACAGGTCAGTTTAATATTTGTCTTAACATTAAATACGTAAA
AACAATTTTAATCTAATTTAATAAATATAAAATTACTTTTTTCTTTTTTTTTCTTATCTACTCT
ATCTTTTATTTCTCAAACAAATCTCTGTTTCTACACTTTCTTTAAACAGATAATGTGGAAA

ATTTTCCTGGGGGAAAACTACCTAAATGGAATTTTACAGATTTCATGCATTCCTTTATGAT
TGTTTTCCGTGTATTATGGGGAGAATGGATAGAATCAATGGGGGACTGCATGCACAAA

>st107_TDEC_CNA_7_[ORGANISM=*Triatoma dimidiata*] *Triatoma dimidiata* SODIUM VOLTAGE-GATED CHANNEL (VGSC)

CTTGGCAATTTGACATTTGTCTTATGTATTATCATCTTTATATTTGCTGTAATGGGCATGC
AATTGTTTGGAAAGAATTATACAGGTCAGTTTAATATTTGTCTTAACATTAAATACGTAAA
AACAATTTTAATCTAATTTAATAAATATAAAATTACTTTTTTCTTTTTTTTTCTAATCTACTCT
ATCTTTTATTTCTCAAACAAATCTCTGTTTCTACCCTTTCTTTAAACAAATAATGGGGAAA
ATTTTCCTGGGGGAAAACTACCTAAATGGAATTTTACAAATTTCATGCATTCCTTTATGAT
TGTTTTCCGTGTATTATGGGGAGAATGGATAAAATCAATGGGGGACTGCATGCACAAA

>st108_TDEC_CNA_8_[ORGANISM=*Triatoma dimidiata*] *Triatoma dimidiata* SODIUM VOLTAGE-GATED CHANNEL (VGSC)

CTTGGCAATTTGACATTTGTCTTATGTATTATCATCTTTATATTTGCTGTAATGGGCATGC
AATTGTTTGGCAAGAATTATACAGGTCAGTTTAATATTTGTCTTAACATTAAATACGTAAA
AACAATTTTAATCTAATTTAATAAATATAAAATTACTTTTTTCTTTTTTTTTCTTATCTACTCT
ATCTTTTATTTCTCAAACAAATCTCTGTTTCTACCCTTTCTTTAAACAAATAATGTGGAAA
ATTTTCCTGGGGGAAAACTACCTAAATGGAATTTTACAGATTTCATGCATTCCTTTATGAT
TGTTTTCCGTGTATTATGGGGAGAATGGATAGAATCAATGGGGGACTGCATGCACAAA

>st109_TDEC_CNA_9_[ORGANISM=*Triatoma dimidiata*] *Triatoma dimidiata* SODIUM VOLTAGE-GATED CHANNEL (VGSC)

CTTGGCAATTTGACATTTGTCTTATGTATTATCATCTTTATATTTGCTGTAATGGGCATGC
AATTGTTTGGAAAGAATTATACAGGTCAGTTTAATATTTGTCTTAACATTAAATACGTAAA
AACAATTTTAATCTAATTTAATAAATATAAAATTACTTTTTTCTTTTTTTTTCTAATCTACTCT
ATCTTTTATTTCTCAAACAAATCTCTGTTTCTACCCTTTCTTTAAACAAATAATGGGGAAA
ATTTTCCTGGGGGAAAACTACCTAAATGGAATTTTACAAATTTCATGCATTCCTTTATGAT
TGTTTTCCGTGTATTATGGGGAGAATGGATAAAATCAATGGGGGACTGCATGCACAAA

>st110_TDEC_CNA_10_[ORGANISM=*Triatoma dimidiata*] *Triatoma dimidiata* SODIUM VOLTAGE-GATED CHANNEL (VGSC)

CTTGGCAATTTGACATTTGTCTTATGTATTATCATCTTTATATTTGCTGTAATGGGCATGC
AATTGTTTGGAAAGAATTATACAGGTCAGTTTAATATTTGTCTTAACATTAAATACGTAAA
AACAATTTTAATCTAATTTAATAAATATAAAATTACTTTTTTCTTTTTTTTTCTTATCTACTCT
ATCTTTTATTTCTCAAACAAATCTCTGTTTCTACACTTTCTTTAAACAGATAATGTGGAAA
ATTTTCCTGGGGGAAAACTACCTAAATGGAATTTTACAGATTTCATGCATTCCTTTATGAT
TGTTTTCCGTGTATTATGGGGAGAATGGATAGAATCAATGGGGGACTGCATGCACAAA

>st101_TDEC_CNA_11_[ORGANISM=*Triatoma dimidiata*] *Triatoma dimidiata* SODIUM VOLTAGE-GATED CHANNEL (VGSC)

CTTGGCAATTTGACATTTGTCTTATGTATTATCATCTTTATATTTGCTGTAATGGGCATGC
AAGTGTTTGGAAAGAATTATACAGGTCATTTTAATATTTGTCTTAACATTAAATACGTAAA
AACAATTTTAATCTAATTTAATAAATATAAAATTACTTTTTTCTTTTTTTTTCTTATCTACTCT
ATCTTTTATTTCTCAAACAAATCTCTGTTTCCTCACTTTCTTTAAAAAAATAATGGGGAAA

ATTTTCCTGGGGGAAAACTACCTAAATGGAATTTTACAGATTTCATGCATTCCTTTATGAT
TGTTTTCCGTGTATTATGAGGAGAATGGAGAAAAGCAATGGGGGACTGCATGCACAAA

>st102_TDEC_CNA_12_[ORGANISM=*Triatoma dimidiata*] *Triatoma dimidiata* SODIUM VOLTAGE-GATED CHANNEL (VGSC)

CTTGGCAATTTGACATTTGTCTTATGTATTATCATCTTTATATTTGCTGTAATGGGCATGC
AAGTGTTTGGAAAGAATTATACAGGTCATTTTAATATTTGTCTTAACATTAAATACGTAAA
AACAATTTTAATCTAATTTAATAAATATAAAATTACTTTTTTCTTTTTTTTTCTTATCTACTCT
ATCTTTTATTTCTCAAACAAATCTCTGTTTCCTCACTTTCTTTAAAAAAATAATGGGGAAA
ATTTTCCTGGGGGAAAACTACCTAAATGGAATTTTACAGATTTCATGCATTCCTTTATGAT
TGTTTTCCGTGTATTATGAGGAGAATGGAGAAAAGCAATGGGGGACTGCATGCACAAA

>st103_TDEC_CNA_13_[ORGANISM=*Triatoma dimidiata*] *Triatoma dimidiata* SODIUM VOLTAGE-GATED CHANNEL (VGSC)

ATTGGCAATTTGACATTTGTCTTATGTATTATCATCTTTATATTTGCTGTAATGGGCATGC
AATTGTTTGGCAAGAATTATACAGGTCAGTTTAATATTTGTCTTAACATTAAATACGTAAA
AACAATTTTAATCTAATTTAATAAATATAAAATTACTTTTTTCTTTTTTTTTCCAATCCACCC
CAACTTTTATTTCCCAAAAAAAACCCTGGTTCCACCCTTTCTTTAAACAAAAAAGGGGG
AAAATTTTCCGGGGGGAAAAACACCCAAATGGGATTTTAACAAATTCATGGCTTCCTTT
ATGAATGGTTTCCGGGGATTAAGGGGAAAAAGGAAAAAAACCATGGGGGGCTGGATG
GCCGTA

>st104_TDEC_CNA_14_[ORGANISM=*Triatoma dimidiata*] *Triatoma dimidiata* SODIUM VOLTAGE-GATED CHANNEL (VGSC)

CTTGGCAATTTGACATTTGTCTTATGTATTATCATCTTTATATTTGCTGTAATGGGCATGC
AATTGTTTGGCAAGAATTATACAGGTCAGTTTAATATTTTTCTTAACATTAAATACGTAAAA
ACAATTTTAATCTAATTTAATAAATATAAAATTACTTTTTTCTTTTTTTTTCTAATCTACTCTA
TCTTTTATTTCCCAAAAAAATCTCTGTTTCAACCTTTTTTTAAAACAAAAAAGGGGAAAA
ATTTTCCGGGGGAAAAACTACCTAAAGGGATTTTTACAAATTTCATGCTTTCTTTTATGA
TTGTTTTCCGGGTATTAGGGGAAAAAGGGAAAAAATCAAGGGGGGACGGCGGGCAAA
AA

>st105_TDEC_CNA_15_[ORGANISM=*Triatoma dimidiata*] *Triatoma dimidiata* SODIUM VOLTAGE-GATED CHANNEL (VGSC)

CTTGGCAATTTGACATTTGTCTTATGTATTATCATCTTTATATTTGCTGTAATGGGCATGC
AATTGTTTGGCAAGAATTATACAGGTCAGTTTAATATTTGTCTTAACATTAAATACGTAAA
AACAATTTTAATCTAATTTAATAAATATAAAATTACTTTTTTCTTTTTTTTTCTTATCTACTCT
ATCTTTTATTTCTCAAACGAATCTCTGTTTCTACACTTTCTTTAAACAAATAATGTGGAAA
ATTTTCCTGGTGGAGAACTACCTAAATGGAATTTTACAAATTTCATGCTTTCCTTTATGAT
TGTTTTCCGTGTATTATGTGGAGAATGGATAGAATCAATGGGGGACTGCATGCACGTA

>st106_TDEC_CNA_16_[ORGANISM=*Triatoma dimidiata*] *Triatoma dimidiata* SODIUM VOLTAGE-GATED CHANNEL (VGSC)

CTTGGCAATTTGACATTTGTCTTATGTATTATCATCTTTATATTTGCTGTAATGGGCATGC
AATTGTTTGGAAAGAATTATACAGGTCAGTTTAATATTTGTCTTAACATTAAATACGTAAA
AACAATTTTAATCTAATTTAATAAATATAAAATTACTTTTTTCTTTTTTTTTCTTATCTACTCT

ATCTTTTATTTCTCAAACAAATCTCTGTTTCTACACTTTCTTTAAACAAATAATGTGGAAA
ATTTTCCTGGTGGAAAACTACCTAAATGGAATTTTACAAATTTCATGCATTCCTTTATGAT
TGTTTTCCGTGTATTATGTGGAAAATGGATAAAATCAATGTGGGACTGCATGCACGTA

>st107_TDEC_CNA_17_[ORGANISM=*Triatoma dimidiata*] *Triatoma dimidiata* SODIUM VOLTAGE-GATED CHANNEL (VGSC)

CTTGGCAATTTGACATTTGTCTTATGTATTATCATCTTTATATTTGCTGTAATGGGCATGC
AATTGTTTGGCAAGAATTATACAGGTCAGTTTAATATTTGTCTTAACATTAAATACGTAAA
AACAATTTTAATCTAATTTAATAAATATAAAATTACTTTTTTCTTTTTTTTTCTTATCTACTCT
ATCTTTTATTTCTCAAACAAATCTCTGTTTCTACACTTTCTTTAAACAAATAATGTGGAAA
ATTTTCCTGGTGGAAAACTACCTAAATGGAATTTTACAAATTTCATGCATTCCTTTATGAT
TGTTTTCCGTGTATTATGTGGAAAATGGATAAAATCAATGTGGGACTGCATGCACGAA

>st108_TDEC_CNA_18_[ORGANISM=*Triatoma dimidiata*] *Triatoma dimidiata* SODIUM VOLTAGE-GATED CHANNEL (VGSC)

CTTGGCAATTTGACATTTGTCTTATGTATTATCATCTTTATATTTGCTGTAATGGGCATGC
AATTGTTTGGCAAGAATTATACAGGTCAGTTTAATATTTGTCTTAACATTAAATACGTAAA
AACAATTTTAATCTAATTTAATAAATATAAAATTACTTTTTTCTTTTTTTTTCTTATCNCCCC
TATCTTTTTTTTTCAAAAAAAATTCTTTTTTTCCCCTTTTTTTTTAAAAAAAAAAGGGGAAA
AATTTTCCGGGGGAAAAACCACCAAGAGGAATTTTTAAAGATTTCGGGCCTCCCTTTAT
GATTGTTCCCCGTGTATTGGGGGAAAAAGGAAAAAAATCGGGGGGGGACTGCCTGCA
AAAA

>st109_TDEC_CNA_19_[ORGANISM=*Triatoma dimidiata*] *Triatoma dimidiata* SODIUM VOLTAGE-GATED CHANNEL (VGSC)

CTTGGCAATTTGACATTTGTCTTATGTATTATCATCTTTATATTTGCTGTAATGGGCATGC
AATTGTTTGGCAAGAATTATACAGGTCAGTTTAATATTTGTCTTAACATTAAATACCCAAA
AACAATTTTAATCTAATTTAATAAATATAAAATTACTTTTTTCTTTTTTTTTCCTTTCAACCG
AAATTTTTATGCCTAAAAATGGGTCATTTGTCCACATTTTCTTTGGAAATCCAGTGGGTA
AACTTCATGTGGGAGAAAACGACCTGAAGCGGATTTTTTAGGCTCTTTGATTTCTTTTT
TCCTTGTTTTTCGAGTACATCGGGAAGTACAGAGAACGTCTATGTTGGGCAGCAGGCA
CAAA

>st110_TDEC_CNA_20_[ORGANISM=*Triatoma dimidiata*] *Triatoma dimidiata* SODIUM VOLTAGE-GATED CHANNEL (VGSC)

CTTGGCAATTTGACATTTGTCTTATGTATTATCATCTTTATATTTGCTGTAATGGGCATGC
AATTGTTTGGCAAGAATTATACAGGTCAGTTTAATATTTGTCTTAACATTAAATACGTAAA
AACAATTTTAATCTAATTTAATAAATATAAAATTACTTTTTTCTTTTTTTTTCTTATCCCCTCT
ATTTTTTTTTTCAAAAAAAAATCTGTGTTTCCACTTTTTCTTTAAAAAAAAAAGGGGGAA
AATTTTCCGGGGGGAAAACCCCCAAGGGGGATTTTTAAATATTTGGTGTCCTCTTTTAT
GATTGTTTCCGGTGTATTGGGGGGAAAAGGGAAAAAAACGGGGGGGGAGTGGACGC
AAAAA

>st111_TDMR_CNA_1_[ORGANISM=*Triatoma dimidiata*] *Triatoma dimidiata* SODIUM VOLTAGE-GATED CHANNEL (VGSC)

CTTGGCAATTTGACATTTGTCTTATGTATTATCATCTTTATATTTGCTGTAATGGGCATGC
AATTGTTTGGCAAGAATTATACAGGTCAGTTTAATATTTGTCTTAACATTAAATACGTAAA
AACAATTTTAATCTAATTTAATAAATATAAAATTACTTTTTCTTTTTTTTTCTTATCTATTCT
ATCTTTTATTTCTCAAACAAATCTCTGTTTCTACCCTTTCTTTAAACAAATAATGGAAAA
ATTTTCCGGGGGGAAAACTCCCTAAAGGAAATTTTACAAATTTCATGCTTTCCTTTATGA
TTGTTTCCCGTGTATTATGGGAAAAAGGGATAAAATCAATGGGGGACTGCATGCACAAA

>st112_TDMR_CNA_2_[ORGANISM=*Triatoma dimidiata*] *Triatoma dimidiata* SODIUM VOLTAGE-GATED CHANNEL (VGSC)

CTTGGCAATTTGACATTTGTCTTATGTATTATCATCTTTATATTTGCTGTAATGGGCATGC
AATTGTTTGGCAAGAATTATACAGGTCAGTTTAATATTTGTCTTAACATTAAATACGTAAA
AACAATTTTAATCTAATTTAATAAATATAAAATTACTTTTTCTTTTTTTTTTTTATCTATTCT
ATCTTTTTTTTCCCAAACAAATCTCTGTTTCTACCCTTTCTTTAAACAAAAAATGGGAAA
AATTTTCCGGGGGGAAAACTCCCTAAAGGAAATTTTACAAATTTCATGCTTTCCTTTATG
ATTGTTTCCCGTGTATTATGGGAAAAAGGGATAAAATCAATGGGGGACTGCATGCACAA
A

>st113_TDMR_CNA_3_[ORGANISM=*Triatoma dimidiata*] *Triatoma dimidiata* SODIUM VOLTAGE-GATED CHANNEL (VGSC)

CTTGGCAATTTGACATTTGTCTTATGTATTATCATCTTTATATTTGCTGTAATGGGCATGC
AATTGTTTGGCAAGAATTATACAGGTCAGTTTAATATTTGTCTTAACATTAAATACGTAAA
AACAATTTTAATCTAATTTAATAAATATAAAATTACTTTTTCTTTTTTTTTCTTATCTATTCT
ATCTTTTATTTCTCAAACAAATCTCTGTTTCTACCCTTTCTTTAAACAAATAATGGGGAAA
ATTTTCCTGGGGGAAAACTACCTAAATGGAATTTTACAGATTTCATGCTTTCCTTTATGA
TTGTTTTCCGTGTATTATGGGAAAAAGGGATAAAATCAATGGGGGACTGCATGCACAAA

>st114_TDMR_CNA_4_[ORGANISM=*Triatoma dimidiata*] *Triatoma dimidiata* SODIUM VOLTAGE-GATED CHANNEL (VGSC)

CTTGGCAATTTGACATTTGTCTTATGTATTATCATCTTTATATTTGCTGTAATGGGCATGC
AATTGTTTGGCAAGAATTATACAGGTCAGTTTAATATTTGTCTTAACATTAAATACGTAAA
AACAATTTTAATCTAATTTAATAAATATAAAATTACTTTTTCTTTTTTTTTTTTATCTATTCT
ATCTTTTTTTTCCCAAACAAATCTCTGTTTCTACCCTTTCTTTAAACAAAAAATGGGAAA
AATTTTCCGGGGGGAAAACTCCCTAAAGGAAATTTTACAAATTTCATGCTTTCCTTTATG
ATTGTTTCCCGTGTATTATGGGAAAAAGGGATAAAATCAATGGGGGACTGCATGCACAA
A

>st115_TDMR_CNA_5_[ORGANISM=*Triatoma dimidiata*] *Triatoma dimidiata* SODIUM VOLTAGE-GATED CHANNEL (VGSC)

CTTGGCAATTTGACATTTGTCTTATGTATTATCATCTTTATATTTGCTGTAATGGGCATGC
AATTGTTTGGCAAGAATTATACAGGTCAGTTTAATATTTGTCTTAACATTAAATACGTAAA
AACAATTTTAATCTAATTTAATAAATATAAAATTACTTTTTCTTTTTTTTTCTTATCTATTCT
ATCTTTTATTTCTCAAACAAATCTCTGTTTCTACCCTTTCTTTAAACAAATAATGGGAAAA
ATTTTCCGGGGGGAAAACTCCCTAAAGGAAATTTTACAAATTTCATGCTTTCCTTTATGA
TTGTTTCCCGTGTATTATGGGAAAAAGGGATAAAATCAATGGGGGACTGCATGCACAAA

>st116_TDMR_CNA_6_[ORGANISM=*Triatoma dimidiata*] *Triatoma dimidiata* SODIUM VOLTAGE-GATED CHANNEL (VGSC)

CTTGGCAATTTGACATTTGTCTTATGTATTATCATCTTTATATTTGCTGTAATGGGCATGC
AATTGTTTGGCAAGAATTATACAGGTCAGTTTAATATTTGTCTTAACATTAAATACGTAAA
AACAATTTTAATCTAATTTAATAAATATAAAATTACTTTTTTCTTTTTTTTTCTTATCTATTCT
ATCTTTTATTTCTCAAACAAATCTCTGTTTCTACCCTTTCTTTAAACAAATAATGGGGAAA
ATTTTCCTGGGGGAAAACTACCTAAATGGAATTTTACAGATTTCATGCTTTCCTTTATGA
TTGTTTTCCGTGTATTATGGGAAAAAGGGATAAAATCAATGGGGGACTGCATGCACAAA

>st117_TDMR_CNA_7_[ORGANISM=*Triatoma dimidiata*] *Triatoma dimidiata* SODIUM VOLTAGE-GATED CHANNEL (VGSC)

CTTGGCAATTTGACATTTGTCTTATGTATTATCATCTTTATATTTGCTGTAATGGGCATGC
AATTGTTTGGCAAGAATTATACAGGTCAGTTTAATATTTGTCTTAACATTAAATACGTAAA
AACAATTTTAATCTAATTTAATAAATATAAAATTACTTTTTTCTTTTTTTTTCTTATCTATTCT
ATCTTTTATTTCTCAAACAAATCTCTGTTTCTACCCTTTCTTTAAACAAATAATGGGAAAA
ATTTTCCGGGGGGAAAACTCCCTAAAGGAAATTTTACAAATTTCATGCTTTCCTTTATGA
TTGTTTCCCGTGTATTATGGGAAAAAGGGATAAAATCAATGGGGGACTGCATGCACAAA

>st118_TDMR_CNA_8_[ORGANISM=*Triatoma dimidiata*] *Triatoma dimidiata* SODIUM VOLTAGE-GATED CHANNEL (VGSC)

CTTGGCAATTTGACATTTGTCTTATGTATTATCATCTTTATATTTGCTGTAATGGGCATGC
AATTGTTTGGCAAGAATTATACAGGTCAGTTTAATATTTGTCTTAACATTAAATACGTAAA
AACAATTTTAATCTAATTTAATAAATATAAAATTACTTTTTTCTTTTTTTTTCTTATCTATTCT
ATCTTTTATTTCTCAAACAAATCTCTGTTTCTACCCTTTCTTTAAACAAATAATGGGAAAA
ATTTTCCGGGGGGAAAACTCCCTAAAGGAAATTTTACAAATTTCATGCTTTCCTTTATGA
TTGTTTCCCGTGTATTATGGGAAAAAGGGATAAAATCAATGGGGGACTGCATGCACAAA

>st119_TDMR_CNA_9_[ORGANISM=*Triatoma dimidiata*] *Triatoma dimidiata* SODIUM VOLTAGE-GATED CHANNEL (VGSC)

CTTGGCAATTTGACATTTGTCTTATGTATTATCATCTTTATATTTGCTGTAATGGGCATGC
AATTGTTTGGCAAGAATTATACAGGTCAGTTTAATATTTGTCTTAACATTAAATACGTAAA
AACAATTTTAATCTAATTTAATAAATATAAAATTACTTTTTTCTTTTTTTTTCTTATCTATTCT
ATCTTTTATTTCTCAAACAAATCTCTGTTTCTACCCTTTCTTTAAACAAATAATGGGGAAA
ATTTTCCTGGGGGAAAACTACCTAAATGGAATTTTACAGATTTCATGCTTTCCTTTATGA
TTGTTTTCCGTGTATTATGGGAAAAAGGGATAAAATCAATGGGGGACTGCATGCACAAA

>st120_TDMR_CNA_10_[ORGANISM=*Triatoma dimidiata*] *Triatoma dimidiata* SODIUM VOLTAGE-GATED CHANNEL (VGSC)

CTTGGCAATTTGACATTTGTCTTATGTATTATCATCTTTATATTTGCTGTAATGGGCATGC
AATTGTTTGGCAAGAATTATACAGGTCAGTTTAATATTTGTCTTAACATTAAATACGTAAA
AACAATTTTAATCTAATTTAATAAATATAAAATTACTTTTTTTTTTTTTTTTCTTATCTATTCT
ATCTTTTTTTTCCCAAACAAATCTCTGTTTCTACCCTTTCTTTAAACAAAAAATGGGAAA
AATTTTCCGGGGGGAAAACTCCCTAAAGGAAATTTTACAAATTTCATGCTTTCCTTTATG
ATTGTTTCCCGTGTATTATGGGAAAAAGGGATAAAATCAATGGGGGACTGCATGCACAA
A

>st111_TDMR_CNA_11_[ORGANISM=*Triatoma dimidiata*] *Triatoma dimidiata* SODIUM VOLTAGE-GATED CHANNEL (VGSC)

ATTGGCAATTTGACATTTGTCTTATGTATTATCATCTTTATATTTGCCGCAATGGGCATGC
CATTGTTTGGCAAGAATTATACAGGGCCGTTTAATATTTGTCTTAACATTAAATACGTAAA
AACAATTTTAATCCAATTTAATAAATATAAAATTACTTTTTTCTTTTTTTTTTTTATCTCTCCA
ATCTTTTTTTTCGCAAACAAATCTTTGTTTCACCCTTTTTTTCAAACAAAAAAGGGGGAA
AATTTCCCGGGGGGAAAAACCCCTAAATGAAATTTTACAAATTTCATGCTTTCCTTTACC
CCGGTTTTCGGGGCCCTCTGGGGAAAAGGAAGAAAAAAAAGGGGGAAGGGTAAGAA
CAAA

>st112_TDMR_CNA_12_[ORGANISM=*Triatoma dimidiata*] *Triatoma dimidiata* SODIUM VOLTAGE-GATED CHANNEL (VGSC)

CTTGGCAATTTGACATTTGTCTTATGTATTATCATCTTTATATTTGCTGTAATGGGCATGC
AATTGTTTGGCAAGAATTATACAGGTCAGTTTAATATTTGTCTTAACATTAAATACGTAAA
AACAATTTTAATCTAATTTAATAAATATAAAATTACTTTTTTCTTTTTTTTTCTTAACCACCC
CACCTTTTATTCCCCAAAAAAACCCCTTTTTCACCCCTTTCTTAAAACAAAAAAGGGGA
AAAATTTCCCGGGGGAAAAACTCCCTAAAGGAATTTTTACAAATTTCAGGCTTCCCTTT
AAAATTGTTTCCCGGGAATAAGGGGAAAAAGGAAAAAAACCAAGGGGGGACGGCAGG
CAAAAA

>st113_TDMR_CNA_13_[ORGANISM=*Triatoma dimidiata*] *Triatoma dimidiata* SODIUM VOLTAGE-GATED CHANNEL (VGSC)

CTTGGCAATTTGACATTTGTCTTATGTATTATCATCTTTATATTTGCTGTAATGGGCATGC
AATTGTTTGGCAAGAATTATACAGGTCAGTTTAATATTTGTCTTAACATTAAATACGTAAA
AACAATTTTAATCTAATTTAATAAATATAAAATTACTTTTTTTTTTTTTTTTCTTACCCCCCC
TACCTTTTATTCCCCAAAAAAATCTCGGTTTCCCCCCTTTTTTTAAAAAAAAAAGGGGAA
AAATTTCCCGGGGGGAAAACACCCAAAAGGGAATTTTACAAATTTCAGGCTTCCCTTTA
AAATGGTTTCCCGGGTATTAGGGGGAAAAGGGAAAAAATCAAGGGGGGACGGGAGG
CAGAAA

>st114_TDMR_CNA_14_[ORGANISM=*Triatoma dimidiata*] *Triatoma dimidiata* SODIUM VOLTAGE-GATED CHANNEL (VGSC)

CTTGGCAATTTGACATTTGTCTTATGTATTATCATCTTTATATTTGCTGTAATGGGCATGC
AATTGTTTGGCAAGAATTATACAGGTCAGTTTAATATTTGTCTTAACATTAAATACGTAAA
AACAATTTTAATCTAATTTAATAAATATAAAATTACTTTTTTCTTTTTTTTTCTTACCCCCCC
TATCTTTTTTTTCAAAAAAAAATCTCTTTTTCCACTTTTTTTTAAAAAAAAAAAGGGAAAA
AATTTTCGGGGGGAAAAACCCCCAAGAGGAATTTTTAAATATTTCGTGCTTTCTTTTAAG
ATTGTTCCCCGTGTATTAGGGGAAAAAGGAAAAAAAACAGGGGGGGACTGCGCGCAA
AAA

>st115_TDMR_CNA_15_[ORGANISM=*Triatoma dimidiata*] *Triatoma dimidiata* SODIUM VOLTAGE-GATED CHANNEL (VGSC)

CTTGGCAATTTGACATTTGTCTTATGTATTATCATCTTTATATTTGCTGTAATGGGCATGC
AATTGTTTGGCAAGAATTATACAGGTCAGTTTAATATTTTTCTTAACATTTAATACGTAAAA
ACAATTTTAATCTAATTTAATAAATATAAAATTACTTTTTTCTTTTTTTTTCTTATCTATTCTA
TCTTTTATTTCCCAAACAAATCTCTGTTTCTACACTTTCTTTAAACAAAAAATGGGGAAA
ATTTTCCTGGGGGAAAACTACCTAAATGGAATTTTACAAATTTCATGCATTCCTTTATGAT
TGTTTTCCGTGTATTATGGGGAAAATGGATAAAATCAATGGGGGACTGCATGCCAAAA

>st116_TDMR_CNA_16_[ORGANISM=*Triatoma dimidiata*] *Triatoma dimidiata* SODIUM VOLTAGE-GATED CHANNEL (VGSC)

CTTGGCAATTTGACATTTGTCTTATGTATTATCATCTTTATATTTGCTGTAATGGGCATGC
AATTGTTTGGCAAGAATTATACAGGTCAGTTTAATATTTGTCTTAACATTAAATACGTAAA
AACAATTTTAATCTAATTTAATAAATATAAAATTACTTTTTTTTTTTTTTTTCTTACCCCCCC
CATCTTTTTTTTCCCAAAAAAAACTCTGTTTTTCCCCTTTTTTTAAAAAAAAAAAGGGGAA
AAATTTCCCGGGGGGAAAAACCCCAAAGGGAAATTTTAAAAATTTCGGGGTCCCCTTT
ATAATTTTTTTCCCGGGGTATTGGGGGAAAAGGAAAAAAAAAAGGGGGGGGGGGGG
CCCAAA

>st117_TDMR_CNA_17_[ORGANISM=*Triatoma dimidiata*] *Triatoma dimidiata* SODIUM VOLTAGE-GATED CHANNEL (VGSC)

CTTGGCAATTTGACATTTGTCTTATGTATTATCATCTTTATATTTGCCGTAATGGGCATGC
AATTGTTTGGCAAGAATTATACAGGTCAGTTTAATATTTGTCTTAACATTAAATACGTAAA
AACAATTTTAATCTAATTTAATAAATATAAAATTACTTTTTTCTTTTTTTTTTCTTATCTATCCC
ATCTTTTATTTCCCAAAAAAATCTCTGTTTCCCCACTTTTTTTAAACAAAAAATGGGGAA
AATTTTCCTGGGGGAAAACTACCTAAATGGAATTTTACAGATTTCAGGGTTTCCTTTATG
ATTGGTTTCCGGGGATTATGGGGAAAATGGATAAAATCCAGGGGGGGCTGGCTGCAC
GAA

>st118_TDMR_CNA_18_[ORGANISM=*Triatoma dimidiata*] *Triatoma dimidiata* SODIUM VOLTAGE-GATED CHANNEL (VGSC)

CTTGGCAATTTGACATTTGTCTTATGTATTATCATCTTTATATTTGCTGTAATGGGCATGC
AATTGTTTGGCAAGAATTATACAGGTCAGTTTAATATTTTTCTTAACATTTAATACGTAAAA
ACAATTTTAATCTAATTTAATAAATATAAAATTACTTTTTTCTTTTTTTTTTCTTATCCATCCTA
TCTTTTATTTCCCAAAAAAATCTCTGTTTCTACATTTTCTTAAAACAAAAAAGGGGAAAAA
TTTCCCGGGGGAAAAACTCCCAAAAGGGATTTTTACAAATTTCGGGCTTCCCTTTATGA
TTGTTTCCCGGGTATTATGGGAAAAAGGAAAAAAACCAAGGGGGGACGGCAGGCACA
AA

>st119_TDMR_CNA_19_[ORGANISM=*Triatoma dimidiata*] *Triatoma dimidiata* SODIUM VOLTAGE-GATED CHANNEL (VGSC)

CTTGGCAATTTGACATTTGTCTTATGTATTATCATCTTTATATTTGCTGTAATGGGCATGC
AATTGTTTGGCAAGAATTATACAGGTCAGTTTAATATTTGTCTTAACATTAAATACGTAAA
AACAATTTTAATCTAATTTAATAAATATAAAATTACTTTTTTTTTTTTTTTTCTTACCCCCCC
TACCTTTTATTTCCCAAAAAAACCCCTGTTTCCCCCCTTTCTTAAAAAAAAAAAAGGGGA
AAAATTTCCCGGGGGAAAAACTCCCTAAAGGAATTTTTACAAATTTCTGGCTTTCTTTTA
AAATTGTTTCCCGGGTATTAGGGGAAAAAGGAAAAAAACCAAGGGGGGACGGCATGC
ACAAA

>st120_TDMR_CNA_20_[ORGANISM=*Triatoma dimidiata*] *Triatoma dimidiata* SODIUM VOLTAGE-GATED CHANNEL (VGSC)

CTTGGCAATTTGACATTTGTCTTATGTATTATCATCTTTATATTTGCTGTAATGGGCATGC
AATTGTTTGGCAAGAATTATACAGGTCAGTTTAATATTTTTCTTAACATTTAATACGTAAAA
ACAATTTTAATCTAATTTAATAAATATAAAATTACTTTTTTTTTTTTTTTTTCTTATCTATTCTAT
CTTTTATTTCCAAAAAAAAATCTCTGTTTCAACCCTTTTTTTAAAACAAAAAAGGGGAAAAA

TTTCCGGGGGGGAAAACCCCCAAAAGGAAATTTTACAAATTTCATGCTTCCCTTTATAA
TTGTTTCCCGGGTATTATGGGAAAAATGGAAAAAACCAAGGGGGGACGGCGGGCACA
AA
