# Supplementary material for: First Report of the L925I kdr Mutation Associated with Pyrethroid Resistance in Genetically Distinct Triatoma dimidiata, Vector of Chagas Disease in Mexico
Source: Trop Med Infect Dis. 2025 Jun 27;10(7):182. doi: 10.3390/tropicalmed10070182 (PMC12299954; doi:10.3390/tropicalmed10070182)
Supplement: Supplementary file 1 [file tropicalmed-10-00182-s001.zip › Table S1. Primer_sequences_ND4_cytb.pdf]

Table S1. Primer sequences used for amplification of mitochondrial markers (cyt b and ND4) and the voltage-gated sodium channel (VGSC) gene in *Triatoma dimidiata*

Primer	5'-3' Sequence
CytB	
mtCytBF01	5'-CGAATTAGTTAAATGATTRTGRGG-3'
mtCytBR02	5'-TATGCRAATAGGAARTATCATTC-3'
ND4	
ND4BF	5'-CACAGCCCACAAAAACCA-3'
ND4BR	5'-TGACTTCCAAGGGCTCATGT-3'
VGSC	
JD5	5′-AAATCCTGGCCAACATTGAA-3′
DG2	5′-ACRTGCATRCARTCCCACAT-3'
