# Supplementary material for: First Report of the L925I kdr Mutation Associated with Pyrethroid Resistance in Genetically Distinct Triatoma dimidiata, Vector of Chagas Disease in Mexico
Source: Trop Med Infect Dis. 2025 Jun 27;10(7):182. doi: 10.3390/tropicalmed10070182 (PMC12299954; doi:10.3390/tropicalmed10070182)
Supplement: Supplementary file 1 [file tropicalmed-10-00182-s001.zip › Table S2. Thermal_cycling_conditions.pdf]

Table S2. Thermal cycling conditions used for the amplification of mitochondrial markers (cyt b, ND4) and the voltage-gated sodium channel (VGSC) gene in *Triatoma dimidiata*.

Cyt b		
Phase	Temperature/Time	Number of cycles
Initial denaturation	94° C (1 minute)	1
Denaturation	94° C (1 minute)	35
Annealing	55° C (1 minute)	
Extension	72° C (1 minute)	
Final extension	72° C (10 minutes)	1
ND4		
Initial denaturation	94° C (5 minutes)	1
Denaturation	94° C (30 seconds)	40
Annealing	50° C (45 seconds)	
Extension	72° C (45 seconds)	
Final extension	72° C (10 minutes)	1
VGSC		
Initial denaturation	94° C (4 minutes)	1
Denaturation	94° C (1 minute)	42
Annealing	60° C (45 seconds)	
Extension	72° C (1 minute)	
Final extension	70° C (10 minutes)	1
